# The Global Burden of Leukemia and Its Attributable Factors in 204 Countries and Territories: Findings from the Global Burden of Disease 2019 Study and Projections to 2030

**DOI:** 10.1155/2022/1612702

**Published:** 2022-04-25

**Authors:** Mengbao Du, Weiwei Chen, Ke Liu, Limengmeng Wang, Yihan Hu, Yingying Mao, Xiaohui Sun, Yi Luo, Jimin Shi, Keding Shao, He Huang, Ding Ye

**Affiliations:** ^1^Bone Marrow Transplantation Center, The First Affiliated Hospital, Zhejiang University School of Medicine, Hangzhou, China; ^2^Department of Epidemiology, Zhejiang Chinese Medical University School of Public Health, Hangzhou, China; ^3^Institute of Hematology, Zhejiang University, Hangzhou, China; ^4^Zhejiang Province Engineering Laboratory for Stem Cell and Immunity Therapy, Hangzhou, China; ^5^Liangzhu Laboratory, Zhejiang University Medical Center, Hangzhou, China; ^6^Institute of Hematology, Zhejiang Chinese Medical University School of First Clinical Medicine, Hangzhou, China

## Abstract

**Background:**

Leukemia is a common malignancy that has four main subtypes and is a threat to human health. Understanding the epidemiological status of leukemia and its four main subtypes globally is important for allocating appropriate resources, guiding clinical practice, and furthering scientific research.

**Methods:**

Average annual percentage changes (AAPCs) were calculated to estimate the change trends of age-standardized rates (ASRs) from 1990 to 2019 in 204 countries and territories. The risk factors for leukemia death and disability-adjusted life-year (DALY) were also analyzed. In addition, the future trends in ASRs were projected through 2030.

**Results:**

The total number of incident cases, deaths, and DALYs from leukemia in 2019 was 0.64, 0.33, and 11.66 million, respectively. Decreasing trends in age-standardized incidence rate (ASIR), the age-standardized death rate (ASDR), and age-standardized DALY rate were detected on a global level while increasing trends in ASIR were detected in the high-sociodemographic index (SDI) regions. The leukemia burden was heavier in males than in females. By cause, acute myeloid leukemia (AML), chronic myeloid leukemia (CML), and chronic lymphocytic leukemia (CLL) were more likely to impose a burden on the elderly, while acute lymphoblastic leukemia (ALL) showed a greater impact in the younger population. A significant positive correlation was observed between SDI and AAPC in ASIR, while SDI was negatively correlated with AAPCs in both ASDR and age-standardized DALY rate. Smoking remained the most significant risk factor associated with leukemia-related death and DALY, especially in males. Similar deaths and DALYs were caused by smoking and high body mass index (BMI) in females. Future projections through 2030 estimated that ASIR and ASDR will continue to increase, while the DALY rate is predicted to decline.

**Conclusions:**

Patterns and trends of leukemia burden are correlated with SDI. The estimated contributions to leukemia deaths indicate that timely measures are needed to reduce smoking and obesity.

## 1. Introduction

Leukemia is a common malignancy that presents with increased numbers of leucocytes in the blood and/or bone marrow [1]. According to the Fourth Edition of the WHO Classification of Tumors of Hematopoietic and Lymphoid Tissues, leukemia can be broadly classified into myeloid or lymphoid lineages. The dominantly presenting leukemia cells may be mature, such as in chronic lymphocytic leukemia (CLL), precursor cells of various lineages, such as in acute leukemias, or both precursor and mature cells, as in chronic myeloid leukemia (CML). Overall, there are four main subtypes of leukemia: acute myeloid leukemia (AML), acute lymphoblastic leukemia (ALL), CML, and CLL [2].

In the past few decades, progress in hematopoietic malignancy treatment has been especially rapid due to improvements in treatment protocols, including the development of targeted therapies such as tyrosine kinase inhibitors (TKIs) [3–6]. However, the current outlook for leukemia is not optimistic, and it is still a major threat to human health. In 2020, leukemia was estimated to be the 15^th^ and 11^th^ most frequent cause of cancer incidence and cancer-related mortality worldwide, respectively, accounting for 474,519 incident cases and 311,594 deaths [7]. In addition, leukemia is the most common cancer in children younger than five years of age and accounts for the highest percentage of deaths, creating a significant burden on individuals, families, and countries [8].

The exact cause of leukemia is still not well understood. It is a multifactorial disease resulting from the interaction of both genetic and environmental factors. CML is a clonal hemopoietic stem cell disorder characterized by a reciprocal translocation between the long arms of chromosomes 9 (chr 9) and 22 (chr 22) [9]. CLL has one of the strongest inherited predispositions of hematological malignancies. Approximately 10% of individuals who develop CLL have a family history of the disease [10]. Environmental factors also play an important role in the occurrence and development of leukemia. ALL and CLL are especially influenced by factors such as pesticide exposure, ionizing radiation, and infections [10–12]. Similarly, in approximately 10% of patients with AML, the development of the disease is preceded by exposure to cytotoxic chemotherapy (particularly alkylating agents or topoisomerase inhibitors) or ionizing radiation, usually applied as a treatment for a primary malignancy [13, 14]. However, in most cases, it appears as a de novo malignancy in previously healthy individuals and originates from the oncogenic transformation of a hemopoietic stem cell or its progenitors [13, 15].

Given the rapid changes and possible regional and population distribution differences in leukemia-related status, epidemiological investigations of leukemia are valuable to policymakers for determining how to allocate medical resources. In 2015, four main subtypes of leukemia were added to the Global Burden of Disease (GBD) cause list, making it possible to better understand the current situation and trends of leukemia [16]. In this report, we used evidence from the GBD 2019 study to examine in detail the global patterns and trends of leukemia from 1990 to 2019, identify the attributable risk factors for establishing targeted intervention programs, and project the trends until 2030 using joinpoint regression model.

## 2. Methods

### 2.1. Data Acquisition and Download

The GBD 2019 database contains incidence, mortality, and disability-adjusted life-year (DALY) statistics of diseases and injuries in 204 countries or territories across years, allowing for consistent comparison over time from 1990 to 2019. DALY is a quantitative comprehensive index that can be used to quantitatively calculate the loss of healthy life years caused by premature death and disability due to various diseases. Detailed descriptions of the methodologies used for gathering, processing, and producing those data have been described in previous studies [17–19]. In addition, the GBD study estimates the levels and trends of 87 attributable risk factors associated with disease burden based on the comparative risk assessment framework. The present study focused on the global burden of leukemia and its four main subtypes. The annual data, including the number of incident cases, deaths, and DALYs, and the corresponding age-standardized rates (ASRs) were downloaded from an online data source tool, the Global Health Data Exchange (GHDx) website (http://ghdx.healthdata.org/gbd-results-tool). ASR is a reliable indicator computed with weights from the age distribution, which eliminates interference from changes in age structure and population size [20]. The social demographic index (SDI), which was measured using the total fertility rate, average educational attainment in the population aged over 15 years, and a composite average of the rankings of per capita income, was also downloaded for the following correlation analysis. The SDI is a scale of overall social development in a country, with values ranging between zero and one; the 204 countries and territories were divided into SDI quintiles (high, high-middle, middle, low-middle, low) accordingly.

### 2.2. Statistical Analysis

Annual incident cases, deaths, DALYs, and corresponding ASRs were used to describe the burden of leukemia along with its four subtypes. Moreover, annual percentage changes (APCs) and average annual percentage changes (AAPCs) based on ASRs per 10^5^ persons, including age-standardized incidence rate (ASIR), age-standardized death rate (ASDR), and age-standardized DALY rate, were employed to quantify the change trends of leukemia burden. Temporal trends from 1990 to 2019 were examined by fitting joinpoint models (version 4.7.0.0; National Cancer Institute), and a maximum number of five joinpoints were specified as options in the analysis. In the formula, *y* = *α* + *βx* + *ε*, *y* means the ln (ASR) value, *x* refers to the calendar year, and *ε* represents the error term. APC values were calculated based on the formula APC = 100∗(*e*^*β*^ − 1). The AAPC is a geometrically weighted average of the different APCs from the joinpoint trend analysis, for which weights are equal to the length of each period during the specified time interval [21]. The 95% confidence interval (CI) was obtained from the linear regression model. For the AAPC value and 95% CI above zero, the corresponding ASR showed an upward trend and vice versa. If the 95% CI of the AAPC included zero, the ASR was deemed to be stable over time. Pearson's correlation coefficient between AAPCs and SDI values in 2019 was calculated to identify the correlation between ASR change trends and social development degrees.

Based on the GBD study and previous GBD reports [22–25], we used a 5-year interval to explore the age distribution of the four leukemia subtypes and assess age-specific potential risk factors contributing to leukemia-related deaths and DALYs.

To predict the global burden of leukemia through 2030, we first computed age-specific rates for five-year age groups (0–4 to >85 years) from 1990 to 2019. Then, the joinpoint regression model was used to detect the year of significant changes in the trends of ASRs by the age group [26]. For age groups without a statistically significant log-linear trend, we used the average rate of 30 years as a predictive value to forecast the future trend. For age groups showing a statistically significant trend with monotonicity, we substituted the predicted year into the model to calculate the predictive rate. If the trend changed with multiple segments, we selected the model parameter of the last segment for prediction [27]. Furthermore, we multiplied the predicted age-specific rates and world population prospects in each age group (https://population.un.org/wpp/Download/Standard/Population/) to obtain the number of cases [28]. Finally, we calculated direct ASRs by dividing the total cases and total populations for all ages. A *P* value <0.05 was considered statistically significant, and all tests were two-sided.

### 2.3. Data Visualization

All of the data analysis was conducted using the open-source software *R* (Version 3.6.2, R core team, Vienna, Austria). Data visualization was performed with packages including maps, ggplot2, statnet, circlize, and RcolorBrewer. Data cleaning was conducted with the dplyr package. Maps were adopted for the visual presentation of leukemia AAPCs in 204 countries and territories. Scatter plots and regression curves were used to analyze the correlation between AAPC and SDI values. Radar charts were used to present the distribution of four leukemia subtypes by age. Chord diagrams were used to demonstrate the relationships between the four leukemia subtypes and contributable risk factors.

## 3. Results

### 3.1. The Burden of Leukemia at the Global and Regional Level

As shown in Supplementary Table 1, the total number of incident cases, deaths, and DALYs from leukemia in 2019 was 0.64 (95% uncertainty interval [UI] 0.59 to 0.70), 0.33 (95% UI 0.31 to 0.36), and 11.66 (95% UI 10.53 to 12.7) million, respectively. Regarding the cause of death, 28.11% were from AML and 8.95% from CML, which correspondingly represented the most and least lethal subtypes of leukemia. In addition, 14.24% and 13.33% of deaths were from ALL and CLL, respectively. Based on regional comparisons of SDI quintiles, it is noteworthy that the high-SDI regions had the highest ASIR (11.99/10^5^ persons, 95% UI 10.87 to 13.21) and ASDR (4.61/10^5^ persons, 95% UI 4.27 to 4.82), while the middle-SDI regions had the highest age-standardized DALY rate (152.72/10^5^ persons, 95% UI 134.81 to 170.25). Among the 21 regions, the highest ASIR was observed in Western Europe (16.87/10^5^ persons, 95% UI 14.68 to 19.38), followed by high-income North America (10.69/10^5^ persons, 95% UI 9.38 to 12.13) and Australasia (10.46/10^5^ persons, 95% UI 8.44 to 12.78). However, the lowest ASIR value was detected in South Asia (3.81/10^5^ persons, 95% UI 3.32 to 4.45). Regarding health loss attributed to leukemia, high-income North America ranked first in terms of ASDR (5.65/10^5^ persons, 95% UI 5.28 to 5.92), and Andean Latin America had the highest age-standardized DALY rate (208.33/10^5^ persons, 95% UI 151.88 to 266.81). Specific information at the national and territorial levels is presented in Supplementary Table 2.

### 3.2. AAPCs of Leukemia at the Global and Regional Level

**Figure 1:**
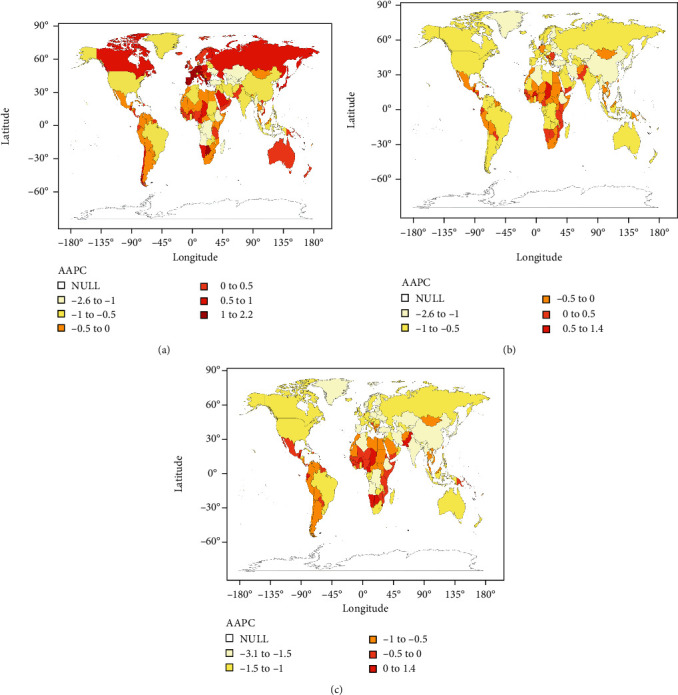
The AAPC in ASRs (per 10^5^ population) for leukemia incidence (a), death (b), and DALY (c) from 1990 to 2019 worldwide.

The AAPCs of ASIR and ASDR, as well as the age-standardized DALY rate of leukemia according to gender, SDI quintile, and region from 1990 to 2019, are presented in Supplementary Table 1; the specific results of 204 countries and territories were visualized into maps shown in Figure 1. The AAPC of the ASIR for males and females was -0.3 (95% CI -0.5 to -0.2) and -0.8 (95% CI -0.9 to -0.7), respectively. For regions with different development levels, the decreasing trend in ASIR was most pronounced in low-middle- (*AAPC* = −1.0, 95% CI -1.1 to -0.9) and middle-SDI regions (AAPC = −1.0, 95% CI -1.2 to -0.9), followed by low-SDI regions (AAPC = −0.9, 95% CI -1.0 to -0.8), and was moderate in high-middle-SDI regions (AAPC = −0.2, 95% CI -0.3 to 0.0). However, an increasing trend was detected in the high-SDI regions (AAPC = 0.2, 95% CI 0.1 to 0.4). Based on the 21 regions, the ASIR showed minor changes in most regions over time, with the most significant increase observed in Western Europe (*AAPC* = 0.9, 95% CI 0.7 to 1.1), and the most significant decrease shown in Central Asia (AAPC = −1.3, 95% CI -1.4 to -1.1).

After analyzing the four subtypes separately, it was obvious that ASRs presented with a different temporal trend, with AAPCs in ASIRs of AML, ALL, CML, and CLL of 0.40 (95% CI 0.30 to 0.50), 1.60 (95% CI 1.50 to 1.80), -0.50 (95% CI -0.80 to -0.20), and 0.60 (95% CI 0.30 to 0.80), respectively (Supplementary Table 3). Notably, CML was the only leukemia subtype that showed a decreasing trend at the global level. In addition, CML also had a relatively large decrease in ASDR (-2.2, 95% CI -2.3 to -2.0) and age-standardized DALY rate (-2.4, 95% CI -2.5 to -2.2) when compared with the other three subtypes of leukemia, suggesting that the circumstances of this disease are improving. The largest AAPC value among the four subtypes differed across geographical regions; the detailed data are presented in Supplementary Table 3 and Supplementary Table 4.

### 3.3. The Burden of Four Subtypes of Leukemia in Different Sex and SDI Quintile Groups

**Figure 2:**
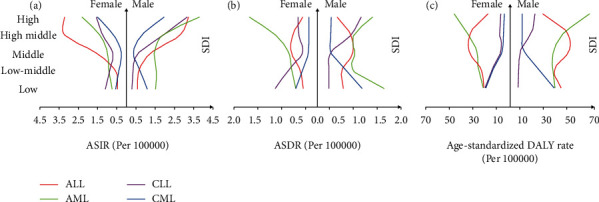
The ASIR (a), ASDR (b), and age-standardized DALY rate (c) of leukemia by gender and SDI quintiles in 2019.

Leukemia was more common in males (350,582.35 incident cases, 95% UI: 307,569.54 to 389,659.92) than in females (292,996.66 incident cases, 95% UI: 263,380.31 to 322,329.81) (Supplementary Table 1). Similar patterns were observed in the number of deaths (188,468.55 in men vs. 146,123.71 in women) and DALYs (6,670,586.07 in men vs. 4,986,960.62 in women) (Supplementary Table 1). When examining the expected relationship between the SDI and ASIR of CLL, a monotonic reduction in rate with decreasing SDI was found in males (Figure 2(a)). The same trend was observed for ASDR and age-standardized DALY rate (Figures 2(b) and 2(c)). For ALL, ASIR peaked in the high-middle quintile in females and the high quintile in males after stratifying by SDI quintiles (Figure 2(a)), while ASDR in males and females both peaked in the middle SDI quintiles (Figure 2(b)). Age-standardized DALY rate also peaked in the middle SDI quintiles and achieved the lowest level in the high quintiles in males and the high-middle quintiles in females (Figure 2(c)). The ASIR of the three subtypes of leukemia other than ALL peaked in the high-SDI regions (Figure 2(a)). Interestingly, high ASIR and age-standardized DALY rates of CLL occurred in females (Figures 2(b) and 2(c)) in regions with a relatively low SDI. In addition, a faster growth trend of AML in age-standardized DALY rate was observed in males compared with females (Figure 2(c)).

### 3.4. Burden of Four Subtypes of Leukemia in Different Age Groups

**Figure 3:**
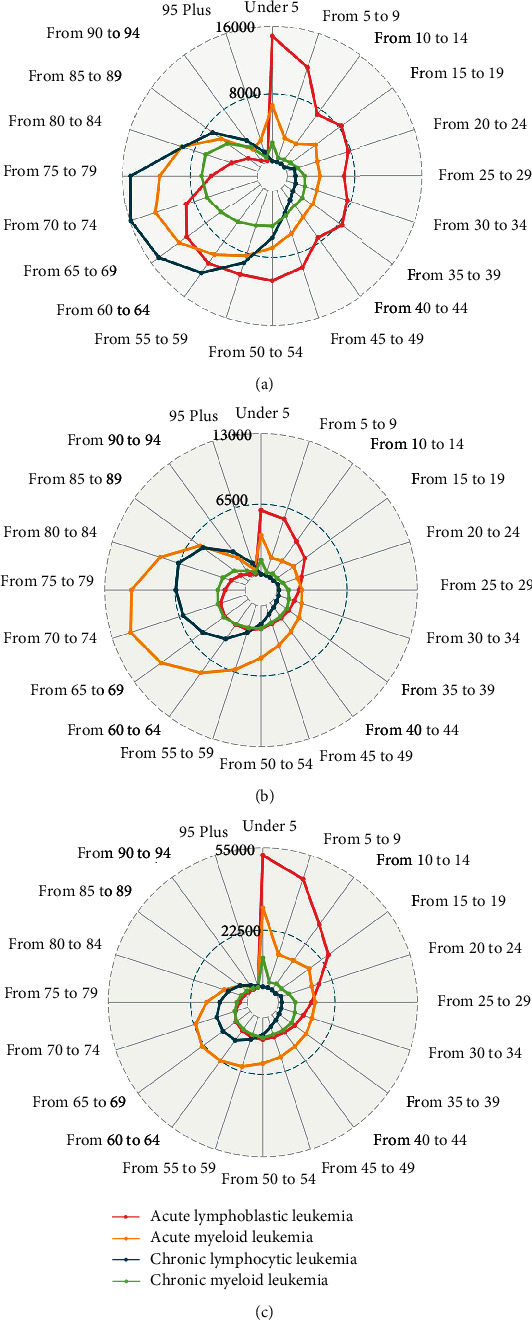
The age distribution of incident cases (a), deaths (b), and DALYs (c) due to 4 leukemia subtypes in 2019.

To investigate the age patterns of leukemia, we divided the patients into 20 groups according to their ages; each age group spanned 5 years. For global incident cases of leukemia, the proportion in the 0–5-year age group (9.24%) was the highest among the 20 age groups, followed by the 70–74-year age group (8.92%). More than half (57.56%) of the deaths due to leukemia occurred among patients over 60 years of age. The number of deaths was the highest among the 70–74-year and 75–79-year age groups, showing that leukemia poses a major threat to human health in the elderly (Supplementary Figure 1). ALL was the principal contributor to incident cases in individuals between the ages of 0 and 59 years old, and CLL was the principal leukemia type in those aged 60–95 years; AML was the principal type in those older than 95 years (Figure 3(a)). The incident cases, deaths, and DALYs of ALL in 2019 generally peaked in the 0–9-year age group, after which a sharp declining trend was observed (Figures 3(b) and 3(c)), indicating that childhood was the stage with the highest incidence of ALL. However, the incidence of ALL increased again with age and reached another peak in the 60–64-year age group (Figure 3(a)). The other three subtypes had a similar age distribution pattern, with the largest number of DALYs in individuals in the 70 and 74-year age group, and incident cases and deaths highest across the age groups from 65 to 84 years (Figure 3). Regarding the subtype distribution of deaths, the results demonstrated that deaths caused by AML were more common overall, while ALL was still the leading cause of death among children and adolescents aged 0–24 years (Figure 3(b)). In addition, the DALYs caused by ALL were also concentrated among individuals between 0 and 24 years old (Figure 3(c)). Distinctly, with the decline in SDI, the incident cases and related deaths from AML, ALL, and CML were more likely to occur in the 0–5-year age group (Supplementary Figure 2). This trend demonstrated that more children may be suffering from leukemia in countries or regions with a lower level of development. Correspondingly, the proportion of leukemia incident cases among elderly individuals was the largest in the high SDI regions.

### 3.5. Correlation between SDI and AAPCs

**Figure 4:**
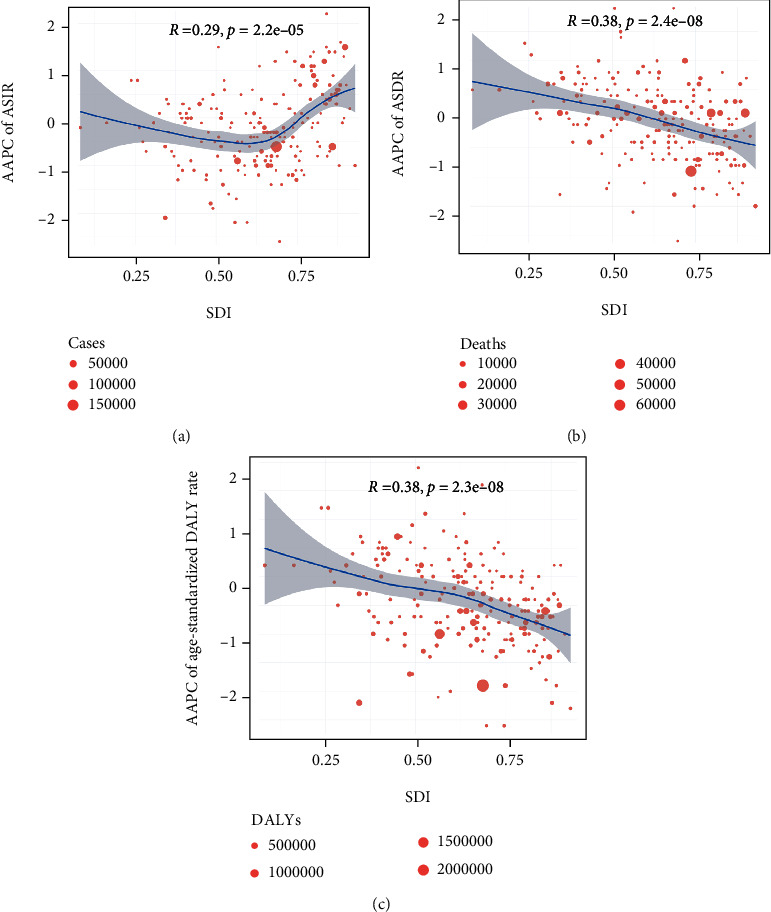
The correlation of AAPCs in ASIR (a), ASDR (b), and age-standardized DALY rate (c) with the SDI value in 204 countries/territories in 2019. The size of the circle indicates the number, and one circle represents a specific country.

The SDI in 2019 serves as a surrogate for the level and availability of health care in each country. As shown in Figure 4(a), a significant positive correlation was observed between SDI and AAPC in the ASIR of leukemia (*ρ* = 0.29, *P* = 2.2 × 10^−5^). Namely, changes in the incidence of leukemia increased at higher levels of socioeconomic development. However, the SDI and AAPCs of both the ASDR (*ρ* = −0.38, *P* = 2.4 × 10^−8^) and age-standardized DALY rate (*ρ* = −0.38, *P* = 2.3 × 10^−8^) were negatively correlated (Figures 4(b) and 4(c)). When stratified by leukemia subtype, correlation analysis showed similar patterns for ALL and CML. The SDI was positively related to the AAPC of the ASIR (ALL: *ρ* = 0.41, *P* = 8.0 × 10^−10^; CML: *ρ* = 0.29, *P* = 2.4 × 10^−5^) but had inverse relationships with the ASDR (ALL: *ρ* = −0.50, *P* = 1.5 × 10^−14^; CML: *ρ* = −0.56, *P* < 2.2 × 10^−16^) and age-standardized DALY rate (ALL: *ρ* = −0.49, *P* = 1.0 × 10^−13^; CML: *ρ* = −0.52, *P* = 1.3 × 10^−15^) (Supplementary Figure 3). For AML, the AAPCs of the ASIR (*ρ* = −0.16, *P* = 0.023) and age-standardized DALY rate (*ρ* = −0.25, *P* = 3.8 × 10^−4^) were negatively correlated with SDI. Nevertheless, a negative correlation between SDI and the AAPCs of the ASDR (*ρ* = −0.32, *P* = 3.0 × 10^−6^) and age-standardized DALY rate (*ρ* = −0.34, *P* = 9.0 × 10^−7^) and SDI were detected in CLL. Detailed information is presented in Supplementary Figure 3.

### 3.6. Risk Factors for Leukemia-Related Deaths and DALYs

**Figure 5:**
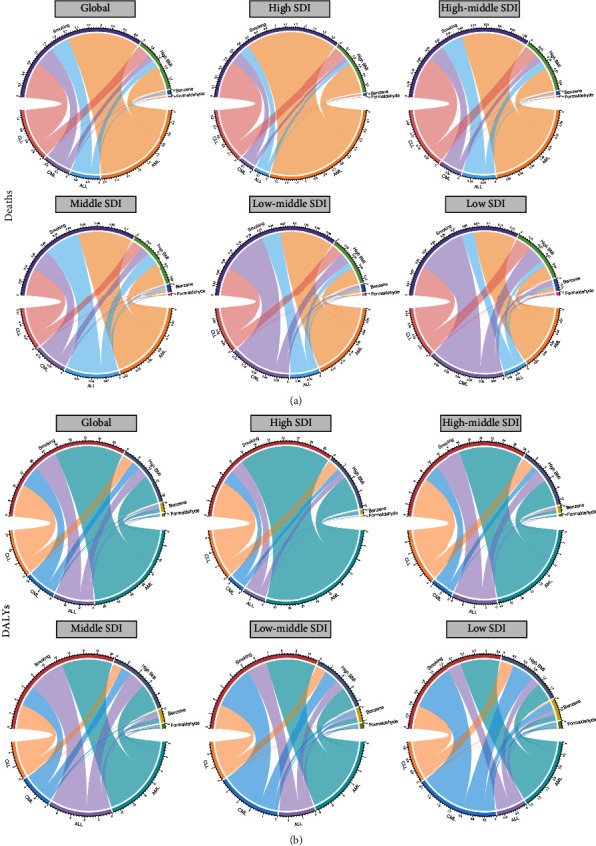
The attributable risk factors of deaths (a) and DALYs (b) due to 4 leukemia subtypes globally and in high, high middle, middle, low middle, and low SDI quintiles in 2019. The numbers are presented after being divided by 10,000.

To determine the potential leukemia-related mortality-attributable risk factors, we analyzed multiple variables in the data provided by the GBD database and identified four risk factors. In 2019, the strongest association between death and DALY due to leukemia was smoking, which accounted for 64.58 thousand (95% UI 39.32 to 91.38) deaths and 1.53 (95% UI 0.90 to 2.16) million DALYs. High body mass index (BMI) ranked as the second most critical risk factor contributing to the 21.73 (95% UI 10.51 to 37.03) thousand deaths and 0.58 (95% UI 0.29 to 0.99) million DALYs. What followed were carcinogens like occupational exposure to benzene (deaths: 1,865.76, 95% UI 564.64 to 3050.99; DALYs: 85,842.68, 95% UI 25,677.22 to 140098.45) and formaldehyde (deaths: 599.83, 95% UI 497.42 to 712.43; DALYs: 2,8451.37, 95% UI 23,384.16 to 34,328.79), which were much less impactful than smoking or high BMI. Taken together, these four risk factors explained approximately 26.54% of the deaths due to leukemia. In regions with different SDI values, the influence of occupational exposure to carcinogens increased slightly with SDI values (Figure 5). In low-SDI regions, the combined proportions of deaths and DALYs attributed to exposure to benzene and formaldehyde were 7.60% and 12.47%, respectively. The corresponding values were lower in high-SDI regions, with an estimation of 1.18% and 2.14%, respectively. Notably, when compared with the other three subtypes of leukemia, the mortality from ALL was most likely to be influenced by carcinogens such as benzene and formaldehyde (5.39%), with CLL being the least affected (1.12%). Other than social development level, attributable risk factors for leukemia mortality also varied by sex and age as follows.

### 3.7. Distribution of Risk Factors according to Sex

Differences in risk factors were observed in terms of sex. The GBD database indicated that the effect of smoking on deaths and DALYs was greater in males than in females. Of the 88,784.2 global leukemia deaths attributed to the four risk factors noted above, 72.74% were attributable to smoking, among which 16.28% and 56.46% were in females and males, respectively. However, the impact of high BMI on leukemia-related deaths was more pronounced in females (deaths in females: 12,496.82, 95% UI 4,773.06 to 22,908.80; deaths in males: 9,237.01, 95% UI 4,303.27 to 15,894.19). It was also noteworthy that in females, smoking and high BMI had similar contributions to deaths. A similar pattern in risk factors was shown for death and DALY (Supplementary Figure 4).

### 3.8. Distribution of Risk Factors among Different Age Groups

Smoking and high BMI were not risk factors for leukemia patients under 20 years old. However, high BMI was identified as the most significant risk factor among the 20–24-year, 25–29-year, and 30–34-year age groups, accounting for the most deaths (20–24-year: 74.95%; 25–29-year: 54.89%; 30–34-year: 41.18%) and DALYs (20–24-year: 74.98%; 25–29-year: 54.91%; 30–34-year: 41.12%) due to leukemia. For leukemia patients older than 34 years, smoking remained the leading cause of leukemia. Interestingly, for leukemia patients over 80 years old, mortality was no longer attributed to exposure to benzene or formaldehyde (Supplementary Figure 5).

### 3.9. Future Trends

**Figure 6:**
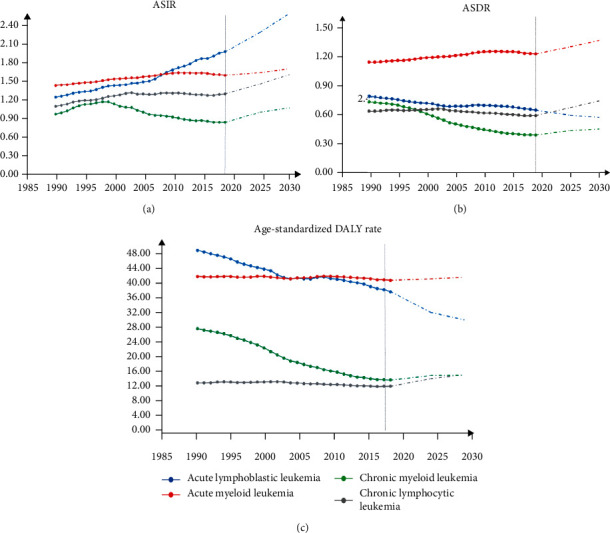
Age-standardized (world population) trends in observed (solid lines) and predicted (dashed lines) ASRs of incidence (a), deaths (b), and DALY (c) from 1990 to 2030, by subtype.

Based on the projections of the leukemia burden, it is estimated that the ASIR and ASDR of leukemia will continue to increase, reaching 9.21/10^5^ and 4.58/10^5^ in 2030. The DALY rate is predicted to decline to 139.61/10^5^ by 2030. ASIR is expected to increase for all subtypes of leukemia, with the highest increase (31.1%) predicted for ALL (Figure 6(a)). However, the ASDR and age-standardized DALY rates for ALL are projected to decline by 20.1% and 11.9%, respectively. Except for ALL, the other three types of leukemia showed increasing trends in ASDR and age-standardized DALY rates, especially in CLL, with remarkable increases of 25.4% and 25.7% (Figure 6).

## 4. Discussion

To the best of our knowledge, the present study is the latest report providing a comprehensive overview of the global burden of leukemia and its four main subtypes, based on the GBD 2019 study. Furthermore, we predicted that the leukemia burden through 2030 will provide information on future disease development and will aid decision-makers in formulating reasonable interventions to reduce the damage caused by leukemia.

In general, the global burden of leukemia is improving according to the negative AAPC values from 1990 to 2019, which may benefit from the development of global living standards and the continuous progress of modern medical technology. It should be noted that increasing trends of ASIR were detected in the high-SDI regions and some geographical areas, especially Western Europe, which could be due to several reasons. The population-based leukemia registry data was more easily acquired in many high-income countries, while it might still be restricted by underdiagnosis and underregistration of patients in low-income countries [29–31]. It is nevertheless important for developing countries or regions to establish better medical systems to diagnose leukemia and capture the available information [29]. Additionally, population aging may have contributed to this result, as aging is more likely to occur in developed countries [32]. When the four main subtypes of leukemia were studied separately, it was noted that the pattern of trends in ASIR also showed a particularly obvious disparity across regions with different SDI quintiles for CML and ALL.

Regarding mortality, decreasing trends in both ASDR and age-standardized DALY rates were detected in most countries or regions. However, like previous reports based on the GBD 2017 study, the downward trend in relatively underdeveloped areas was not as obvious as in more developed areas [24, 25]. Resource-poor countries might have difficulty ensuring adequate medical resources and access to novel medications for the treatment of leukemia patients. To solve these problems, international collaboration and mobilization of additional resources are needed to reduce the survival gap [29]. Among the four leukemia subtypes, the burden of CML dramatically decreased according to the latest death data and significant downward trends in ASDR and age-standardized DALY rate, which was consistently found in former reports based on the GBD 2017 database [24, 33]. Due to the development of new therapies for CML in the past few decades, such as the approval of imatinib in 2003 for the first-line treatment of CML, the prognosis of CML has significantly improved. Among the four subtypes, most leukemia-related deaths were caused by AML, and an increasing trend was detected for ASDR, especially in regions with relatively low SDIs. Despite many discoveries related to the pathophysiology and molecular heterogeneity of AML, the standard therapy has remained unchanged for the past several decades [34]. Although the complete remission rate for AML patients who undergo standard chemotherapy is over 70% [13], the 5-year survival rate for patients under 60 years is still only 50% [35]. Fortunately, accumulating novel treatment strategies have had superior therapeutic effects. For example, midostaurin is Food and Drug Administration- (FDA-) approved for use in combination with chemotherapy for newly-diagnosed Fms-like tyrosine kinase 3- (FLT3-) mutated AML, and gilteritinib is FDA-approved as a single agent salvage therapy for patients with relapsed or refractory FLT3-mutated AML. Many other TKIs are currently in preclinical or clinical trials and show potential as clinical application prospects [36, 37]. In addition, other targeted therapies such as IDH2/IDH1 inhibitors and BCL-2 inhibitors have also gradually been approved by the FDA [38–40]. Immunotherapies such as bispecific T-cell engager (BiTE) antibodies [41, 42], chimeric antigen receptor (CAR) T cells [43–46], and immune checkpoint inhibitors [47, 48] are under investigation. Notably, when compared with data based on the GBD study 2017, the AAPCs of ASIR, ASDR, and age-standardized DALY rate of AML were all reduced, and the trend in age-standardized DALY rate changed from increasing to stable (AAPC = −0.1, 95% CI -1.0 to 0.0) [49]. In summary, we have confidence in the future improvement of treatment outcomes of AML, and the possibility of reversing the progression of the AML burden.

According to a report from GBD 2017 Childhood Cancer Collaborators, leukemia accounted for the highest proportion of the childhood cancer DALY burden globally, with an attributable fraction of 34.1% (34.0–34.1). Specifically, ALL accounted for the highest proportion of all subtypes [50]. Our study also highlighted the high risk of ALL in childhood and adolescence. The early onset of leukemia and the presence of “preleukemic” genetic signatures at birth indicate that exposure to prenatal and postnatal risk factors is crucial for the development of childhood leukemia, mainly for ALL [51]. Several environmental risk factors involved in the etiology of ALL have been described, such as exposure to pesticides [52, 53] and tobacco smoke [54–56]. In addition, a collection of studies have indicated associations between childhood leukemia, especially ALL, and a loosely related group of environmental exposures including benzene and formaldehyde [57–59]. After childhood and adolescence, the incident cases of ALL peaks in the elderly, while the other three subtypes also mostly occurred in this stage. Characteristically, regarding the distribution of leukemia, aging is an inextricable topic; this has been noted in previous reports about leukemia and its main subtypes [22, 23, 33, 49, 60]. It has been reported that with age, hematopoietic stem cells (HSCs) gradually lose their capacity to regenerate, leading to typical features of blood aging including immunosenescence, anemia, and unbalanced myeloid cell production. These features in turn increase the risk of autoimmune and hematological malignancies [61]. The deep mechanisms of leukemia caused by aging have also been revealed in recent reports [62, 63]. The association between aging and leukemogenesis partly explains why the proportion of leukemia incident cases among elderly individuals was the largest in the high SDI regions, as population aging is a significant contributor. The prognosis in elderly patients is worse than that of young patients due to poorer performance status at diagnosis, lower complete remission rates with intensive chemotherapy, increased early death rates with intensive chemotherapy, chemotherapy resistance, and increased incidence of unfavorable cytogenetics and secondary AML [64, 65]. With population aging increasing in many developed countries, a heavier burden of leukemia may arise in the future. Furthermore, based on our projection of future trends through 2030, the ASIR and ASDR of several subtypes of leukemia will continue to increase. To address the future burden of leukemia, policymakers need to further improve medical systems, endeavor to achieve early diagnosis and treatment, and promote health education to reduce individual exposures to related risk factors.

Importantly, smoking was the most important factor—especially in males—contributing to leukemia-related deaths and DALYs. Several studies have demonstrated that smoking is associated with disease progression and premature death [66–69], which may be due to severe infections leading to aplasia and leukemogenic compounds favoring complex karyotypic abnormalities. In addition to the impact on clinical outcomes, an association between cigarette smoking and increased susceptibility to leukemia was also previously indicated in AML and CML [70–72]. Furthermore, several studies have suggested that heavier paternal smoking around the time of conception is a risk factor for childhood ALL, as mentioned above [54–56]. Previous evidence suggests that men should be strongly encouraged to cease smoking, particularly when planning to start a family or have a child [73]. Judging from the current situation, it is necessary to vigorously educate people on the dangers of smoking. Highlighting higher prices of tobacco may be particularly useful according to a report from the International Agency for Research on Cancer (IARC) [74].

Of note, high BMI was another important risk factor for leukemia-related deaths and DALYs, especially in females. Many studies have indicated that overweight and obesity can act as predictors of adverse outcomes in both children and adults with leukemia [75–78]. The protective effect of obesity on leukemic cells may be through increased levels of insulin and insulin-like growth factor-1 (IGF-1), which provides a chemoprotective niche and metabolic “fuel” to promote systemic inflammation [79]. Correspondingly, the association between overweight and the overall risk of leukemia and its four subtypes is also supported by several large cohort studies and meta-analyses of cohort and case-control studies [80–82]. The metabolic, endocrinologic, immunologic, and inflammatory-like changes caused by obesity may increase the mutation rate of cells, dysregulate gene function, disturb DNA repair, or induce epigenetic changes, which are all conducive to tumor transformation [83]. Since BMI is an easily modifiable prognostic factor, it is suggested that leukemia patients should receive active interventions to obtain more normal body weight. It is also recommended that healthy people maintain a normal BMI to aid in the prevention of leukemia. In addition to these two important risk factors, occupational exposure to benzene and formaldehyde also contributed to the deaths and DALYs of leukemia. The occurrence of leukemia was the most susceptible to these two carcinogens in the low SDI regions. This may also be related to the high incidence of cases, deaths, and DALYs in children from these regions. Benzene is a basic raw material in the petrochemical industry, and formaldehyde is common in interior decoration materials. Studies have provided further evidence that leukemia-related cytogenetic changes can occur in the circulating myeloid progenitor cells of healthy workers exposed to benzene and formaldehyde, which may be an underlying mechanism of leukemogenesis [84–86]. Given their important application value in many fields, it would be meaningful to develop environmentally friendly, novel materials to reduce the use of these two carcinogens.

Approximately 80% of all smokers live in low- and middle-income countries [33, 87], and the obesity epidemic has shown an increasing trend in developing countries [33, 88, 89]. In addition, the risk of exposure to carcinogens in the low SDI regions was significantly higher than in the high SDI regions [49]. These environmental factors were thought to be mainly-attributable risk factors for childhood leukemia and related deaths [51]. CLL has one of the strongest inherited predispositions of hematological malignancies, mainly affecting older individuals with a median age at diagnosis of 72 years [10, 90]. There is little consistent evidence available to link CLL with environmental exposure to either radiation or chemicals, except in the case of agricultural workers and herbicides [90]. This may explain the inconsistency of CLL compared to the other three subtypes.

The GBD 2019 study provided us with newly updated data, allowing our study to be carried out successfully. However, based on some objective conditions, several limitations were unavoidable. First, due to the limitation of diagnostic accuracy, underreporting and misdiagnosis may cause deviations in the true number of incident cases, incidence rates, and corresponding age-standardized rates, especially in some underdeveloped countries. This situation was likely more serious in the past few decades but is improving over time. Second, even though the impact of racial and ethnic disparities on disease has been elucidated in several studies [91, 92], we could not obtain the corresponding data from the GBD database for subgroup analysis. Third, some special subtypes of leukemia, such as eosinophilic leukemia, hairy cell leukemia, and plasma cell leukemia, have not been evaluated separately; all were classified as “other leukemia,” which was not investigated as they may be of little significance to readers.

## 5. Conclusion

In this study, even though the incident cases, deaths, and DALYs are increasing annually, downward trends in ASIR, ASDR, and age-standardized DALY rates were detected, especially for CML. This indicated that the overall burden of leukemia has been lightened over the past 30 years. We found that leukemia was more likely to develop in males and elderly individuals. It is noteworthy that the decline in ASDR and age-standardized DALY rate in regions with a relatively low SDI was not as significant as that in developed regions. Smoking remained the most significant risk factor associated with leukemia mortality in males, while smoking and high BMI made a similar contribution to mortality in females. Other contributors, including occupational exposure to benzene and formaldehyde, also increased the risk of leukemia deaths and DALYs. As such, it is necessary for policymakers to further improve healthcare systems, work toward early diagnosis and treatment, and promote health education to reduce individual exposures to relevant risk factors.

## Data Availability

The datasets analyzed in this study can be found from the Global Health Data Exchange (GHDx) website (http://ghdx.healthdata.org/gbd-results-tool).

## Supplementary Materials

Supplementary Materials Supplementary Table 1: the numbers, age-standardized rates, and AAPCs in incidence, death, and DALYs for leukemia in 204 countries and territories. Supplementary Table 2: the AAPCs of ASIR, ASDR, and age-standardized DALY rate due to 4 leukemia subtypes from 1990 to 2019 in 204 countries/territories. Supplementary Figure 1: proportional distribution of incident cases, deaths, and DALYs by the age group. Supplementary Figure 2: radar maps of incident cases (a), deaths (b), and DALYs (c) among SDI quintiles due to 4 subtypes of leukemia across different age groups. Supplementary Figure 3: the correlation between AAPC of ASIR (a), ASDR (b), age-standardized DALY rate (c), and SDI for 4 leukemia subtypes in 2019. Supplementary Figure 4: chordal graphs of risk factors contributing to leukemia-related deaths (a) and DALYs (b) by sex. Supplementary Figure 5: chordal graphs of risk factors contributing to leukemia-related deaths (a) and DALYs (b) among different age groups.
