# Supplementary material for: The Global Burden of Leukemia and Its Attributable Factors in 204 Countries and Territories: Findings from the Global Burden of Disease 2019 Study and Projections to 2030
Source: J Oncol. 2022 Apr 25;2022:1612702. doi: 10.1155/2022/1612702 (PMC9061017; doi:10.1155/2022/1612702)
Supplement: Supplementary Materials — Supplementary Table 1: the numbers, age-standardized rates, and AAPCs in incidence, death, and DALYs for leukemia in 204 countries and territories. Supplementary Table 2: the AAPCs of ASIR, ASDR, and age-standardized DALY rate due to 4 leukemia subtypes from 1990 to 2019 in 204 countries/territories. Supplementary Figure 1: proportional distribution of incident cases, deaths, and DALYs by the age group. Supplementary Figure 2: radar maps of incident cases (a), deaths (b), and DALYs (c) among SDI quintiles due to 4 subtypes of leukemia across different age groups. Supplementary Figure 3: the correlation between AAPC of ASIR (a), ASDR (b), age-standardized DALY rate (c), and SDI for 4 leukemia subtypes in 2019. Supplementary Figure 4: chordal graphs of risk factors contributing to leukemia-related deaths (a) and DALYs (b) by sex. Supplementary Figure 5: chordal graphs of risk factors contributing to leukemia-related deaths (a) and DALYs (b) among different age groups. [file 1612702.f1.zip › h.supplementary information.pdf]

Supplementary information

Supplementary Table 1. The numbers, age-standardized rates, and AAPCs in incidence, death and DALYs for leukemia.

Supplementary Table 2. The numbers, age-standardized rates, and AAPCs in incidence, death and DALYs for leukemia in 204 countries and territories.

Supplementary Table 3. The AAPCs (95% CI) of ASIR, ASDR and age-standardized DALY rate due to 4 leukemia subtypes from 1990 to 2019.

Supplementary Table 4. The AAPCs of ASIR, ASDR and age-standardized DALY rate due to 4 leukemia subtypes from 1990 to 2019 in 204 countries/territories.

Supplementary Figure 1. Proportional distribution of incident cases, deaths and DALYs by age group.

Supplementary Figure 2. Radar maps of incident cases (A), deaths (B), DALYs (C) among SDI quintiles due to 4 subtypes of leukemia across different age groups.

Supplementary Figure 3. The correlation between AAPC of ASIR (A), ASDR (B), age-standardized DALY rate (C) and SDI for 4 leukemia subtypes in 2019.

Supplementary Figure 4. Chordal graphs of risk factors contributing to leukemia-related deaths (A) and DALYs (B) by sex.

Supplementary Figure 5. Chordal graphs of risk factors contributing to leukemia-related deaths (A) and DALYs (B) among different age groups.
