# Supplementary material for: The Global Burden of Leukemia and Its Attributable Factors in 204 Countries and Territories: Findings from the Global Burden of Disease 2019 Study and Projections to 2030
Source: J Oncol. 2022 Apr 25;2022:1612702. doi: 10.1155/2022/1612702 (PMC9061017; doi:10.1155/2022/1612702)
Supplement: Supplementary Materials — Supplementary Table 1: the numbers, age-standardized rates, and AAPCs in incidence, death, and DALYs for leukemia in 204 countries and territories. Supplementary Table 2: the AAPCs of ASIR, ASDR, and age-standardized DALY rate due to 4 leukemia subtypes from 1990 to 2019 in 204 countries/territories. Supplementary Figure 1: proportional distribution of incident cases, deaths, and DALYs by the age group. Supplementary Figure 2: radar maps of incident cases (a), deaths (b), and DALYs (c) among SDI quintiles due to 4 subtypes of leukemia across different age groups. Supplementary Figure 3: the correlation between AAPC of ASIR (a), ASDR (b), age-standardized DALY rate (c), and SDI for 4 leukemia subtypes in 2019. Supplementary Figure 4: chordal graphs of risk factors contributing to leukemia-related deaths (a) and DALYs (b) by sex. Supplementary Figure 5: chordal graphs of risk factors contributing to leukemia-related deaths (a) and DALYs (b) among different age groups. [file 1612702.f1.zip › i.Supplementary Tables.pdf]

Supplementary Table 1. The numbers, age-standardized rates, and AAPCs in incidence, death and DALYs for leukemia.

Characteristic	Incident cases			Deaths			DALYs		
	Number of incident cases, 2019 (95% UI)	ASIR/100,000, 2019 (95% UI)	AAPC (%) 1990 to 2019 (95% CI)	Number of deaths, 2019 (95% UI)	ASDR/100,000, 2019 (95% UI)	AAPC (%) 1990 to 2019 (95% CI)	Number of DALYs, 2019 (95% UI)	Age-standardized DALY rate/100,000, 2019 (95% UI)	AAPC (%) 1990 to 2019 (95% CI)
**Total**	643579.01 (586980.09, 699729.39)	8.22 (7.50, 8.94)	-0.5 (-0.6, -0.5)	334592.26 (306817.69, 360213.90)	4.26 (3.91, 4.58)	-1.1 (-1.1, -1.0)	11657546.69 (10529142.15, 12700651.10)	150.51 (135.71, 164.14)	-1.6 (-1.7, -1.5)
**Sex**									
Male	350582.35 (307569.54, 389659.92)	9.42 (8.31, 10.46)	-0.3 (-0.5, -0.2)	188468.55 (164929.23, 207722.43)	5.20 (4.59, 5.72)	-0.9 (-1.0, -0.9)	6670586.07 (5697463.05, 7460123.73)	175.43 (149.63, 196.45)	-1.4 (-1.5, -1.4)
Female	292996.66 (263380.31, 322329.81)	7.23 (6.47, 7.96)	-0.8 (-0.9, -0.7)	146123.71 (131684.84, 158238.00)	3.50 (3.17, 3.79)	-1.3 (-1.4, -1.2)	4986960.62 (4524242.79, 5443176.13)	127.17 (114.95, 138.89)	-1.8 (-2.0, -1.7)
**SDI**									

Low	46164.44 (35614.13, 57709.15)	4.83 (3.85, 5.73)	-0.9 (-1.0, -0.8)	29923.91 (24140.53, 35500.26)	3.88 (3.12, 4.56)	-0.7 (-0.8, -0.7)	1647140.94 (1308167.78, 2021129.55)	150.39 (121.26, 178.59)	-1.2 (-1.3, -1.1)
Low-middle	73431.00 (64332.25, 85155.13)	4.68 (4.12, 5.40)	-1.0 (-1.1, -0.9)	51122.02 (45290.84, 59125.14)	3.45(3.07, 3.98)	-0.8 (-0.9, -0.7)	2201321.70 (1920873.80, 2557600.52)	131.47 (115.00, 152.65)	-1.4 (-1.5, -1.3)
Middle	154769.81 (136563.92, 172530.72)	6.54 (5.78, 7.28)	-1.0 (-1.2, -0.9)	92243.15 (81501.80, 103862.14)	3.90 (3.45, 4.38)	-1.1 (-1.2, -1.1)	3627966.30 (3206480.33, 4046086.09)	152.72 (134.81, 170.25)	-1.7 (-1.8, -1.6)
High-middle	167438.89 (150331.22, 183008.76)	10.08 (9.03, 11.11)	-0.2 (-0.3, 0.0)	75826.14 (68743.13, 81314.27)	4.21(3.80, 4.53)	-1.2 (-1.3, -1.1)	2340608.31 (2088069.86, 2534242.06)	150.88 (133.73, 164.04)	-1.9 (-2.0, -1.7)
High	188538.05 (169161.62, 208210.75)	11.99 (10.87, 13.21)	0.2 (0.1, 0.4)	85262.75 (76984.95, 89676.49)	4.61 (4.27, 4.82)	-0.9 (-1.0, -0.8)	1832262.50 (1731033.88, 1910164.37)	126.46 (120.67, 131.58)	-1.4 (-1.5, -1.4)
**Regions**									

Central Asia	5246.66 (4605.56, 6047.70)	6.05 (5.35, 6.93)	-1.3 (-1.4, -1.1)	3188.21 (2848.79, 3603.62)	3.89 (3.50, 4.37)	-1.0 (-1.2, -0.8)	149495.54 (130533.91, 171440.83)	164.44 (144.52, 188.23)	-1.6 (-1.7, -1.5)
Central Europe	17318.39 (15236.62, 19601.47)	9.59 (8.45, 10.87)	0.6 (0.5, 0.7)	9586.46 (8458.75, 10785.63)	4.85 (4.29, 5.45)	-0.5 (-0.7, -0.4)	233129.47 (205859.87, 262478.34)	144.97 (127.72, 163.98)	-1.1 (-1.3, -1.0)
Eastern Europe	21615.30 (19527.90, 23850.45)	7.54 (6.86, 8.29)	-0.3 (-1.1, 0.5)	11746.14 (10640.14, 12897.19)	3.90 (3.55, 4.28)	-1.0 (-1.6, -0.3)	352317.04 (321016.55, 387489.21)	139.99 (127.84, 153.03)	-1.7 (-2.4, -1.1)
Australasia	4717.22 (3814.35, 5798.72)	10.46 (8.44, 12.78)	0.3 (0.0, 0.7)	2367.33 (2121.00, 2571.30)	4.83 (4.39, 5.20)	-0.7 (-0.9, -0.6)	49849.31 (46144.08, 53204.16)	123.87 (116.10, 131.44)	-1.2 (-1.3, -1.1)
High-income Asia Pacific	29195.95 (24933.06, 33270.91)	10.33 (8.98, 11.70)	0.5 (0.2, 0.7)	12116.94 (10458.88, 13028.23)	3.02 (2.72, 3.22)	-1.5 (-1.6, -1.4)	267329.03 (241668.10, 284478.41)	98.21 (89.49, 104.91)	-2.2 (-2.4, -2.0)
High-income North America	61549.28 (53737.72, 70237.98)	10.69 (9.38, 12.13)	-0.4 (-0.6, -0.2)	34706.07 (32027.06, 36512.47)	5.65 (5.28, 5.92)	-0.6 (-0.8, -0.5)	738966.19 (703032.17, 769665.53)	143.76 (138.05, 149.13)	-1.1 (-1.3, -1.0)
Southern Latin America	5386.76 (4272.51, 6699.70)	7.20 (5.71, 8.93)	-0.1 (-0.4, 0.1)	3839.65 (3588.68, 4062.64)	4.90 (4.60, 5.18)	-0.6 (-0.7, -0.5)	117863.65 (111407.17, 124877.42)	166.92 (157.59, 177.31)	-0.9 (-1.1, -0.8)
Western Europe	118623.58 (102900.91, 135440.55)	16.87 (14.68, 19.38)	0.9 (0.7, 1.1)	44997.10 (39920.49, 47621.60)	3.12 (2.54, 3.72)	-0.7 (-0.8, -0.6)	893135.36 (828830.87, 940341.72)	130.50 (123.33, 137.05)	-1.3 (-1.4, -1.2)

Andean Latin America	4423.47 (3233.64, 5622.19)	7.30 (5.33, 9.25)	-0.1 (-0.5, 0.3)	3045.12 (2259.24, 3806.60)	5.15 (3.83, 6.42)	-0.4 (-0.6, -0.3)	130139.26 (94644.63, 167106.98)	208.33 (151.88, 266.81)	-0.5 (-0.8, -0.1)
Caribbean	3607.09 (2835.43, 4446.13)	7.56 (5.83, 9.49)	-0.3 (-0.4, -0.1)	2363.94 (1954.68, 2813.98)	4.82 (3.95, 5.81)	-0.4 (-0.6, -0.3)	93167.95 (71599.39, 119464.24)	200.47 (151.14, 261.34)	-0.6 (-0.8, -0.5)
Central Latin America	15977.36 (13603.35, 18616.77)	6.58 (5.60, 7.67)	-0.2 (-0.2, -0.2)	10720.66 (9282.54, 12353.31)	4.45 (3.85, 5.13)	-0.3 (-0.3, -0.2)	470070.93 (403984.34, 543365.40)	190.57 (163.81, 220.59)	-0.4 (-0.5, -0.4)
Tropical Latin America	12371.13 (11556.86, 13082.09)	5.53 (5.14, 5.88)	-0.7 (-0.8, -0.7)	9065.68 (8444.99, 9504.43)	3.97 (3.69, 4.19)	-0.6 (-0.7, -0.6)	322893.06 (303422.06, 342799.74)	146.69 (137.01, 157.33)	-1.1 (-1.2, -1.0)
North Africa and Middle East	39297.49 (32616.56, 45056.03)	7.76 (6.54, 8.84)	-0.7 (-0.8, -0.6)	25143.10 (21109.25, 28825.50)	5.41 (4.62, 6.13)	-0.9 (-1.0, -0.8)	1011554.80 (822536.76, 1173620.62)	183.36 (150.69, 211.24)	-1.3 (-1.4, -1.3)
South Asia	59855.88 (51906.53, 70104.64)	3.81 (3.32, 4.45)	-0.8 (-1.0, -0.5)	44550.51 (38745.01, 52631.28)	2.98 (2.59, 3.50)	-0.8 (-1.1, -0.6)	1847835.01 (1598397.83, 2192213.56)	109.23 (94.33, 129.55)	-1.3 (-1.5, -1.0)
East Asia	159358.30 (131903.50, 185876.66)	10.41 (8.69, 12.26)	-0.6 (-1.0, -0.3)	62983.34 (52696.54, 73469.41)	3.69 (3.12, 4.28)	-1.8 (-2.0, -1.6)	2399037.72 (2014459.98, 2777481.26)	164.06 (136.91, 189.20)	-2.4 (-2.6, -2.1)

Oceania	807.96 (533.04, 1224.88)	6.84 (4.80, 9.75)	-0.1 (-0.4, 0.1)	457.06 (330.30, 643.64)	4.79 (3.58, 6.48)	-0.3 (-0.5, -0.1)	24621.75 (17036.72, 36614.31)	192.00 (137.22, 272.19)	-0.3 (-0.5, -0.1)
Southeast Asia	42262.80 (35843.46, 49692.34)	6.81 (5.80, 7.99)	-0.8 (-0.8, -0.8)	28502.48 (24414.64, 32955.74)	4.74 (4.05, 5.47)	-0.7 (-0.7, -0.6)	1156787.74 (996181.59, 1344034.44)	178.88 (153.85, 208.04)	-1.1 (-1.2, -1.1)
Central Sub-Saharan Africa	4091.39 (3060.97, 5533.05)	3.89 (2.91, 4.98)	-1.2 (-1.3, -1.0)	2417.08 (1840.70, 3060.68)	2.99 (2.18, 4.04)	-0.8 (-0.9, -0.7)	132016.80 (97917.88, 177958.53)	110.28 (83.79, 139.41)	-1.4 (-1.5, -1.2)
Eastern Sub-Saharan Africa	21547.86 (13995.26, 29459.80)	5.85 (4.15, 7.71)	-1.2 (-1.3, -1.1)	12063.97 (8397.79, 15924.26)	4.37 (3.19, 5.61)	-1.0 (-1.1, -0.9)	727912.71 (475127.70, 980634.80)	173.01 (121.47, 228.39)	-1.7 (-1.8, -1.5)
Southern Sub-Saharan Africa	2763.22 (2382.96, 3135.08)	4.34 (3.71, 4.86)	-0.1 (-0.2, 0.1)	1955.85 (1650.57, 2174.00)	3.35 (2.78, 3.73)	-0.2 (-0.6, 0.1)	71812.05 (62129.37, 81633.23)	102.98 (88.05, 115.55)	-0.6 (-0.7, -0.4)
Western Sub-Saharan Africa	13561.93 (10265.05, 17280.77)	3.90 (3.14, 4.68)	0.1 (0.0, 0.2)	8775.57 (6931.93, 10830.65)	3.12 (2.54, 3.72)	0.0 (-0.1, 0.1)	467611.32 (348733.42, 610675.24)	113.00 (88.91, 139.81)	-0.2 (-0.3, -0.1)

Abbreviations: AAPC, average annual percentage change; CI, confidence interval; SDI, socio-demographic index; UI, uncertainty interval.

Supplementary Table 2. The numbers, age-standardized rates, and AAPCs in incidence, death and DALYs for leukemia in 204 countries and territories.

Regions	Incident cases			Deaths			DALYs		
	Number of incident cases, 2019 (95% UI)	ASIR/100,000, 2019 (95% UI)	AAPC (%) 1990 to 2019 (95% CI)	Number of deaths, 2019 (95% UI)	ASDR/100,000, 2019 (95% UI)	AAPC (%) 1990 to 2019 (95% CI)	Number of DALYs, 2019 (95% UI)	Age-standardized DALY rate/100,000, 2019 (95% UI)	AAPC (%) 1990 to 2019 (95% CI)
Afghanistan	3,360.19 (2,124.84, 5,172.16)	12.36 (8.39, 18.15)	-0.6 (-0.8, -0.4)	10,591.49 (8,501.49, 12,911.01)	10.01 (6.95, 14.38)	-0.4 (-0.5, -0.3)	115,862.63 (74,512.38, 176,533.72)	365.49 (241.81, 546.42)	-0.8 (-1.0, -0.6)
Albania	280.31 (210.33, 371.65)	9.39 (7.18, 12.35)	1.2 (0.5, 1.8)	4.01 (3.36, 4.88)	4.19 (3.17, 5.55)	-0.2 (-0.7, 0.3)	4,988.15 (3,739.31, 6,493.50)	178.59 (136.15, 226.78)	-0.1 (-0.7, 0.5)
Algeria	1,508.80 (1,152.08, 1,876.04)	4.20 (3.25, 5.14)	-0.9 (-1.1, -0.8)	261.70 (201.53, 361.63)	3.02 (2.35, 3.71)	-1.2 (-1.3, -1.2)	38,065.28 (29,009.69, 47,473.56)	97.91 (75.39, 121.05)	-1.7 (-1.8, -1.6)
American Samoa	1.90 (1.51, 2.40)	3.77 (3.03, 4.73)	-0.4 (-0.6, -0.3)	21.84 (18.24, 25.91)	2.96 (2.42, 3.61)	-0.6 (-0.7, -0.4)	53.33 (42.04, 68.76)	100.01 (79.41, 128.46)	-0.6 (-0.7, -0.4)

Andorra	26.00 (19.21, 34.44)	22.71 (16.74, 30.34)	1.3 (1.0, 1.5)	0.50 (0.35, 0.71)	6.28 (4.76, 7.97)	-0.7 (-0.8, -0.7)	197.68 (147.96, 255.47)	174.45 (130.56, 226.22)	-0.9 (-0.9, -0.8)
Angola	1,120.86 (755.68, 1,543.51)	4.40 (3.28, 5.49)	-1.2 (-1.4, -0.9)	1,833.21 (1,623.58, 2,009.56)	3.31 (2.45, 4.35)	-0.8 (-1.0, -0.5)	35,937.11 (24,396.02, 49,184.83)	124.63 (92.38, 156.71)	-1.5 (-1.7, -1.2)
Antigua and Barbuda	5.38 (4.50, 6.40)	6.07 (5.02, 7.29)	0.1 (-0.1, 0.3)	310.92 (276.27, 343.69)	4.27 (3.63, 5.01)	0.0 (-0.3, 0.2)	130.56 (109.67, 157.03)	147.26 (121.85, 179.55)	-0.1 (-0.2, 0.0)
Argentina	3,515.61 (2,787.11, 4,371.55)	7.10 (5.63, 8.84)	-0.2 (-0.5, 0.1)	229.69 (177.48, 283.73)	5.16 (4.80, 5.50)	-0.5 (-0.6, -0.4)	82,885.68 (77,809.54, 88,133.01)	175.47 (164.63, 186.96)	-0.9 (-1.1, -0.7)
Armenia	260.22 (218.65, 305.77)	7.62 (6.46, 8.88)	-0.8 (-1.3, -0.3)	1,040.47 (854.10, 1,240.43)	4.34 (3.69, 5.07)	-1.2 (-1.6, -0.7)	5,657.01 (4,789.93, 6,593.90)	177.19 (151.68, 205.45)	-1.6 (-2.0, -1.1)
Australia	3,882.74 (2,992.80, 4,908.58)	10.08 (7.82, 12.72)	0.3 (0.0, 0.7)	62.94 (43.76, 85.88)	4.79 (4.30, 5.18)	-0.8 (-1.0, -0.5)	41,409.51 (37,867.79, 44,575.96)	121.39 (112.21, 129.57)	-1.2 (-1.3, -1.1)
Austria	2,134.56 (1,719.33, 2,604.41)	14.84 (11.96, 18.21)	1.4 (1.0, 1.7)	233.73 (195.02, 276.69)	4.64 (4.18, 5.02)	-0.5 (-0.5, -0.4)	16,826.80 (15,206.68, 18,128.14)	121.22 (110.75, 130.24)	-1.0 (-1.1, -0.8)

Azerbaijan	828.90 (619.83, 1,083.81)	8.89 (6.62, 11.65)	-0.7 (-1.2, -0.2)	157.49 (133.04, 186.36)	4.62 (3.56, 5.91)	-0.5 (-0.8, -0.2)	21,630.41 (16,155.62, 28,146.48)	223.73 (166.71, 293.15)	-1.0 (-1.5, -0.5)
Bahamas	19.45 (15.94, 24.16)	5.07 (4.16, 6.28)	-0.2 (-0.7, 0.2)	31,378.14 (28,944.16, 33,015.60)	3.67 (3.03, 4.48)	-0.4 (-0.8, 0.0)	538.76 (438.17, 667.67)	139.95 (113.69, 173.17)	-0.4 (-1.0, 0.1)
Bahrain	73.91 (57.64, 95.17)	7.67 (6.08, 9.49)	-0.6 (-1.1, -0.1)	300.77 (202.20, 439.51)	5.49 (4.29, 6.84)	-1.3 (-2.0, -0.7)	1,706.45 (1,298.90, 2,233.97)	147.82 (116.51, 183.74)	-1.6 (-2.0, -1.2)
Bangladesh	4,535.06 (3,360.65, 5,952.29)	3.20 (2.38, 4.19)	-1.8 (-2.2, -1.5)	222.56 (171.43, 280.44)	2.55 (1.86, 3.39)	-1.7 (-2.0, -1.4)	143,748.65 (106,557.15, 187,863.73)	95.25 (70.98, 124.22)	-2.2 (-2.5, -1.9)
Barbados	28.14 (23.31, 33.83)	7.25 (5.94, 8.85)	-0.1 (-0.6, 0.4)	187.08 (157.20, 221.66)	5.14 (4.27, 6.15)	-0.2 (-0.5, 0.2)	616.01 (507.87, 748.26)	176.96 (145.53, 217.34)	-0.5 (-1.0, 0.0)
Belarus	1,223.85 (962.40, 1,578.54)	9.31 (7.20, 11.90)	-0.8 (-1.1, -0.6)	423.95 (379.74, 468.28)	4.58 (3.61, 5.84)	-2.0 (-2.3, -1.8)	18,605.83 (14,593.58, 23,879.51)	157.63 (123.87, 201.98)	-3.1 (-3.2, -3.0)
Belgium	2,625.87 (2,068.75, 3,296.37)	13.97 (10.95, 17.54)	0.7 (0.3, 1.0)	898.41 (829.69, 972.58)	5.26 (4.73, 5.72)	-1.0 (-1.2, -0.8)	23,618.96 (21,521.86	131.09 (120.62, 141.58)	-1.4 (-1.6, -1.2)

							, 25,635.77)		
Belize	17.15 (14.82, 19.56)	4.79 (4.15, 5.46)	-0.6 (-1.1, -0.1)	372.67 (271.26, 512.45)	3.34 (2.91, 3.77)	0.0 (-0.2, 0.2)	503.38 (438.55, 572.54)	129.92 (113.14, 146.61)	-0.4 (-1.0, 0.1)
Benin	501.38 (316.72, 770.38)	4.64 (3.22, 6.47)	0.3 (0.2, 0.4)	4,190.75 (3,706.89, 4,628.98)	3.55 (2.57, 4.75)	0.2 (0.0, 0.3)	17,623.12 (11,106.12, 27,420.07)	139.27 (94.34, 199.94)	0.1 (-0.2, 0.4)
Bermuda	6.51 (5.46, 7.93)	6.97 (5.78, 8.50)	-0.5 (-0.7, -0.4)	6.58 (5.66, 7.67)	3.59 (3.03, 4.35)	-1.8 (-1.9, -1.7)	97.53 (81.65, 117.71)	119.38 (98.86, 144.40)	-1.8 (-1.9, -1.6)
Bhutan	25.98 (18.11, 36.94)	4.20 (2.98, 5.96)	-0.5 (-0.8, -0.3)	3,503.00 (2,603.12, 4,614.39)	3.32 (2.34, 4.69)	-0.4 (-0.6, -0.3)	762.54 (510.48, 1,119.42)	113.80 (78.00, 165.14)	-1.2 (-1.6, -0.8)
Bolivia (Plurinational State of)	945.76 (706.18, 1,217.60)	8.81 (6.72, 11.18)	-0.5 (-0.6, -0.3)	89.12 (69.15, 109.29)	6.84 (5.31, 8.58)	-0.3 (-0.3, -0.2)	29,301.21 (21,317.31, 38,015.10)	253.39 (188.66, 323.15)	-0.9 (-1.0, -0.7)
Bosnia and Herzegovina	359.71 (273.95, 456.72)	7.16 (5.36, 9.08)	1.4 (1.0, 1.8)	9,336.28 (8,160.02, 10,277.91)	4.22 (3.22, 5.28)	0.5 (0.0, 0.9)	5,966.41 (4,418.05, 7,586.93)	136.72 (99.42, 173.80)	0.0 (-0.6, 0.6)
Botswana	102.15 (71.56, 138.00)	5.85 (4.07, 7.80)	1.1 (0.9, 1.2)	269.66 (203.74, 349.18)	4.19 (2.94, 5.58)	0.5 (0.4, 0.6)	2,690.88 (1,869.02, 3,652.27)	136.01 (95.41, 182.11)	0.6 (0.4, 0.7)

Brazil	11,993.65 (11,228.51, 12,641.67)	5.52 (5.13, 5.86)	-0.7 (-0.8, -0.7)	732.65 (638.52, 804.07)	3.96 (3.68, 4.16)	-0.7 (-0.9, -0.6)	312,188.12 (294,027.89, 330,198.89)	146.35 (136.71, 156.49)	-1.1 (-1.3, -1.0)
Brunei Darussalam	33.39 (27.99, 38.30)	9.20 (7.76, 10.48)	0.1 (0.0, 0.3)	9,859.56 (8,419.32, 10,580.29)	5.63 (4.82, 6.39)	-0.4 (-0.6, -0.3)	845.12 (688.11, 966.38)	208.63 (171.43, 239.39)	-0.7 (-0.9, -0.5)
Bulgaria	835.42 (665.03, 1,038.03)	8.17 (6.51, 10.06)	0.1 (-0.3, 0.6)	2,657.68 (2,460.01, 2,838.52)	4.75 (3.80, 5.86)	0.2 (0.0, 0.4)	15,502.76 (12,265.02, 19,163.30)	172.80 (137.35, 213.26)	-0.5 (-0.8, -0.2)
Burkina Faso	1,059.64 (620.67, 1,631.54)	5.06 (3.51, 7.01)	0.8 (0.6, 1.0)	563.18 (451.47, 694.80)	3.73 (2.80, 4.91)	0.6 (0.4, 0.7)	35,902.89 (20,868.37, 56,594.70)	150.62 (102.15, 212.98)	0.7 (0.5, 0.9)
Burundi	517.87 (287.73, 893.63)	4.49 (2.89, 6.96)	-0.7 (-1.1, -0.2)	643.43 (504.49, 817.55)	3.26 (2.26, 4.63)	-0.5 (-0.9, -0.1)	16,991.78 (9,821.16, 28,958.12)	133.18 (85.63, 204.40)	-1.0 (-1.5, -0.4)
Cabo Verde	26.23 (21.39, 32.79)	5.46 (4.51, 6.80)	0.8 (0.5, 1.1)	1,203.44 (795.74, 1,734.74)	4.04 (3.41, 4.93)	1.0 (0.6, 1.4)	717.46 (570.64, 888.97)	138.88 (112.69, 171.79)	0.0 (-0.4, 0.4)
Cambodia	1,199.37 (924.09, 1,522.79)	8.24 (6.48, 10.30)	-1.3 (-1.4, -1.1)	35.20 (25.74, 44.65)	5.92 (4.70, 7.24)	-1.1 (-1.2, -0.9)	33,988.96 (25,970.72	219.11 (170.08, 275.68)	-1.7 (-1.8, -1.5)

							, 43,149.75)		
Cameroon	1,102.55 (723.15, 1,588.06)	4.96 (3.43, 6.85)	0.5 (0.3, 0.7)	13.92 (11.43, 17.09)	3.81 (2.67, 5.11)	0.3 (0.2, 0.4)	37,166.17 (24,016.62 , 54,053.63)	142.57 (95.11, 199.78)	0.4 (0.1, 0.6)
Canada	8,438.45 (6,506.22, 10,625.54)	15.10 (11.69, 19.02)	0.6 (0.2, 1.1)	276.92 (214.58, 352.54)	4.96 (4.50, 5.38)	-0.8 (-1.0, -0.7)	70,304.06 (64,439.77 , 76,403.54)	129.88 (120.23, 139.96)	-1.2 (-1.3, -1.1)
Central African Republic	234.40 (128.15, 413.23)	5.02 (3.23, 7.57)	-0.5 (-0.6, -0.4)	921.24 (696.37, 1,156.51)	3.78 (2.63, 5.15)	-0.4 (-0.5, -0.4)	7,994.46 (4,584.51, 13,409.30)	153.27 (98.53, 235.37)	-0.6 (-0.7, -0.4)
Chad	661.32 (425.83, 972.90)	4.65 (3.38, 6.11)	0.8 (0.7, 1.0)	266.55 (211.61, 334.39)	3.62 (2.69, 4.66)	0.8 (0.7, 0.9)	23,626.26 (15,328.06 , 35,152.93)	141.27 (100.00, 189.18)	0.7 (0.5, 0.9)
Chile	1,497.49 (1,164.56, 1,886.38)	7.29 (5.67, 9.15)	0.7 (0.5, 1.0)	40.53 (29.68, 53.02)	4.13 (3.82, 4.46)	-0.5 (-0.7, -0.3)	27,943.04 (25,940.74 , 30,300.51)	145.89 (134.13, 158.86)	-0.9 (-1.2, -0.7)
China	154,648.09 (127,207.43, 181,075.43)	10.47 (8.70, 12.34)	-0.6 (-1.0, -0.3)	2,658.89 (1,965.17, 3,469.43)	3.67 (3.07, 4.28)	-1.8 (-2.0, -1.7)	2,309,312.76 (1,924,583.51,	163.82 (136.60, 189.48)	-2.4 (-2.7, -2.2)

							2,691,119.96)		
Colombia	3,372.92 (2,621.88, 4,224.41)	6.98 (5.43, 8.73)	-0.1 (-0.4, 0.3)	8.78 (6.67, 10.66)	4.39 (3.42, 5.50)	-0.7 (-1.1, -0.3)	89,180.35 (69,081.25, 111,721.12)	190.35 (148.43, 237.81)	-0.6 (-0.9, -0.2)
Comoros	27.36 (17.21, 42.45)	4.34 (2.88, 6.41)	-0.1 (-1.1, 0.9)	3.84 (2.75, 5.13)	3.11 (2.22, 4.23)	0.0 (-0.9, 0.9)	836.23 (506.42, 1,302.56)	123.89 (77.95, 188.66)	-0.4 (-1.6, 0.9)
Congo	158.44 (116.54, 212.16)	4.04 (3.03, 5.23)	-1.1 (-1.4, -0.8)	0.09 (0.06, 0.11)	3.27 (2.43, 4.29)	-0.9 (-1.1, -0.7)	5,017.19 (3,605.98, 6,890.93)	111.40 (82.42, 148.22)	-1.3 (-1.7, -1.0)
Cook Islands	0.68 (0.53, 0.86)	3.11 (2.36, 3.97)	-1.1 (-1.2, -1.1)	514.36 (325.92, 743.71)	2.34 (1.82, 2.91)	-1.2 (-1.3, -1.1)	15.21 (11.33, 19.44)	73.45 (53.36, 95.34)	-1.5 (-1.5, -1.4)
Costa Rica	396.53 (305.16, 501.02)	8.05 (6.22, 10.17)	0.3 (0.0, 0.5)	729.28 (563.74, 917.48)	5.11 (3.96, 6.37)	-0.6 (-1.0, -0.3)	8,816.84 (6,863.42, 11,157.64)	183.69 (143.26, 231.04)	-0.6 (-0.9, -0.2)
Croatia	932.33 (727.82, 1,172.08)	12.74 (9.97, 15.97)	1.0 (0.8, 1.2)	17.59 (11.80, 24.71)	4.81 (3.84, 5.98)	-0.5 (-0.9, -0.1)	8,787.49 (6,982.84, 11,055.00)	131.14 (104.73, 163.04)	-1.1 (-1.5, -0.8)
Cuba	987.09 (798.67, 1,213.90)	6.47 (5.26, 7.92)	-0.3 (-0.5, 0.0)	506.56 (328.03, 787.72)	4.00 (3.28, 4.87)	-0.9 (-1.1, -0.7)	18,638.80 (15,164.19	140.97 (115.25, 169.72)	-1.4 (-1.6, -1.2)

							, 22,810.62)		
Cyprus	262.28 (207.47, 309.75)	15.82 (12.74, 18.64)	2.2 (1.9, 2.6)	2,049.08 (1,514.31, 2,392.54)	5.61 (4.02, 6.75)	-0.2 (-0.5, 0.1)	2,302.98 (1,737.51, 2,730.20)	139.78 (106.19, 164.92)	-0.5 (-0.8, -0.3)
Czechia	2,110.18 (1,713.51, 2,541.48)	11.39 (9.30, 13.79)	0.5 (0.2, 0.8)	4,142.89 (2,482.25, 5,700.41)	5.07 (4.20, 6.07)	-1.1 (-1.4, -0.8)	22,326.48 (18,428.15, 27,006.41)	131.07 (108.23, 159.33)	-1.8 (-2.1, -1.5)
Côte d'Ivoire	867.28 (560.73, 1,260.25)	4.36 (3.05, 5.94)	0.1 (-0.1, 0.3)	1,391.29 (1,097.82, 1,805.48)	3.48 (2.51, 4.57)	0.0 (-0.1, 0.2)	30,674.04 (19,750.59, 44,616.05)	129.52 (87.87, 180.32)	0.0 (-0.2, 0.2)
Democratic People's Republic of Korea	2,035.53 (1,535.67, 2,652.33)	7.60 (5.77, 9.85)	-2.2 (-2.3, -2.1)	672.64 (447.58, 993.21)	4.49 (3.40, 5.81)	-1.2 (-1.3, -1.2)	53,840.13 (39,846.50, 71,991.87)	199.98 (150.52, 266.09)	-1.7 (-1.8, -1.7)
Democratic Republic of the Congo	2,487.71 (1,784.66, 3,470.78)	3.61 (2.55, 4.85)	-1.3 (-1.5, -1.1)	306.22 (206.93, 455.80)	2.81 (1.91, 3.96)	-0.9 (-0.9, -0.8)	80,303.92 (58,073.31, 110,215.19)	102.46 (72.74, 136.04)	-1.5 (-1.6, -1.3)
Denmark	1,240.03 (972.88, 1,563.60)	13.16 (10.30, 16.66)	-0.1 (-0.3, 0.2)	31.37 (19.69, 47.81)	5.06 (4.56, 5.59)	-1.6 (-1.8, -1.4)	11,592.02 (10,512.19	127.35 (115.71, 139.43)	-2.4 (-2.7, -2.0)

							, 12,751.15)		
Djibouti	58.82 (35.08, 93.32)	5.38 (3.52, 8.00)	0.4 (0.1, 0.8)	826.83 (730.52, 902.41)	3.66 (2.51, 5.27)	0.5 (0.2, 0.8)	1,802.61 (1,055.98, 2,870.68)	150.81 (93.43, 230.31)	0.2 (-0.1, 0.6)
Dominica	6.44 (5.12, 7.98)	8.55 (6.72, 10.65)	0.2 (0.0, 0.5)	1,494.00 (1,221.51, 1,682.80)	6.39 (5.12, 7.83)	0.1 (-0.1, 0.3)	173.36 (135.78, 218.05)	245.07 (190.33, 308.46)	0.1 (-0.1, 0.4)
Dominican Republic	590.32 (425.30, 814.62)	5.79 (4.19, 7.95)	0.1 (-0.4, 0.5)	767.68 (579.96, 968.29)	4.05 (2.93, 5.51)	0.4 (0.1, 0.7)	16,643.39 (12,057.16 , 22,859.59)	157.64 (114.91, 216.49)	-0.2 (-0.5, 0.1)
Ecuador	1,293.10 (985.56, 1,620.32)	7.81 (5.95, 9.83)	0.4 (0.0, 0.8)	1,986.49 (1,762.69, 2,174.32)	5.75 (4.34, 7.21)	0.3 (0.0, 0.6)	40,263.53 (30,040.69 , 50,473.92)	231.51 (172.79, 289.73)	0.1 (-0.3, 0.5)
Egypt	4,067.52 (2,929.86, 5,603.62)	4.98 (3.55, 6.83)	-0.3 (-0.7, 0.0)	8,796.02 (8,198.71, 9,216.58)	3.78 (2.63, 5.33)	-0.2 (-0.5, 0.0)	122,215.2 1 (88,205.20 , 168,711.7 5)	136.07 (99.78, 183.29)	-0.8 (-0.9, -0.7)
El Salvador	447.24 (322.83, 584.62)	7.30 (5.26, 9.57)	-0.7 (-1.0, -0.5)	3.93 (3.35, 4.61)	4.84 (3.51, 6.28)	-0.1 (-0.2, 0.0)	11,752.79 (8,537.57, 15,390.82)	188.95 (137.16, 248.10)	-0.8 (-1.0, -0.5)

Equatorial Guinea	32.93 (19.21, 54.11)	3.65 (2.21, 5.57)	-1.1 (-1.5, -0.7)	18.56 (15.58, 22.60)	2.92 (1.79, 4.39)	-0.8 (-1.1, -0.5)	1,048.04 (602.27, 1,697.16)	94.23 (55.78, 147.54)	-1.8 (-2.2, -1.4)
Eritrea	274.61 (163.86, 460.49)	4.89 (3.22, 7.40)	0.3 (0.1, 0.5)	8.47 (6.43, 10.73)	3.59 (2.49, 5.03)	0.4 (0.2, 0.7)	9,236.21 (5,428.82, 15,337.84)	145.33 (93.17, 223.93)	0.0 (-0.2, 0.3)
Estonia	252.95 (195.23, 318.42)	12.08 (9.41, 15.07)	0.3 (-0.3, 0.9)	18.99 (15.91, 21.60)	5.62 (4.38, 6.96)	-0.9 (-1.6, -0.1)	3,147.91 (2,446.49, 3,913.19)	169.17 (135.61, 208.38)	-1.7 (-2.5, -0.8)
Eswatini	41.57 (28.23, 56.43)	5.11 (3.56, 6.85)	0.5 (0.2, 0.7)	2,805.52 (2,033.65, 3,774.01)	4.11 (2.87, 5.46)	0.3 (0.1, 0.6)	1,261.37 (848.50, 1,726.98)	134.04 (90.64, 183.77)	0.3 (0.1, 0.6)
Ethiopia	8,307.56 (4,274.41, 12,435.50)	9.50 (5.31, 13.36)	-2.1 (-2.2, -2.0)	294.58 (211.36, 381.35)	7.74 (4.51, 10.92)	-1.8 (-1.9, -1.7)	305,609.29 (159,473.60, 444,030.12)	290.14 (163.46, 397.21)	-2.7 (-3.0, -2.3)
Fiji	68.87 (53.13, 88.41)	8.53 (6.66, 10.82)	-0.3 (-0.5, -0.2)	435.05 (333.14, 565.67)	6.47 (5.07, 8.10)	-0.6 (-0.7, -0.4)	2,173.54 (1,674.84, 2,787.81)	247.57 (191.70, 315.24)	-0.5 (-0.7, -0.4)
Finland	972.41 (760.36, 1,233.02)	10.63 (8.30, 13.49)	0.5 (0.2, 0.7)	1,631.49 (1,436.38, 1,768.25)	3.60 (3.29, 3.92)	-1.3 (-1.4, -1.1)	8,702.17 (7,922.07, 9,550.04)	101.13 (92.61, 110.50)	-1.6 (-1.8, -1.4)

France	17,595.52 (13,871.65, 21,913.58)	16.08 (12.74, 20.31)	1.2 (1.0, 1.4)	1.97 (1.57, 2.42)	5.32 (4.70, 5.82)	-0.7 (-0.9, -0.6)	141,857.62 (127,383.41, 155,130.45)	134.70 (123.72, 145.71)	-1.3 (-1.4, -1.1)
Gabon	57.05 (40.13, 74.26)	4.20 (2.97, 5.34)	-0.8 (-1.0, -0.6)	1,102.57 (939.22, 1,312.00)	3.33 (2.35, 4.27)	-0.6 (-0.7, -0.4)	1,716.09 (1,188.13, 2,286.21)	112.17 (78.67, 146.34)	-1.1 (-1.3, -0.8)
Gambia	47.78 (33.73, 63.56)	3.16 (2.37, 3.95)	0.3 (-0.6, 1.2)	1,237.75 (1,075.92, 1,362.49)	2.67 (2.04, 3.30)	0.3 (-0.5, 1.1)	1,667.20 (1,146.34, 2,300.97)	91.98 (66.73, 118.06)	0.1 (-0.8, 1.1)
Georgia	334.33 (278.47, 391.90)	7.64 (6.40, 8.94)	-1.4 (-2.1, -0.6)	1.40 (1.13, 1.71)	4.82 (4.06, 5.68)	-0.7 (-1.4, 0.0)	7,961.66 (6,681.34, 9,331.24)	197.88 (167.99, 230.27)	-1.5 (-2.1, -0.9)
Germany	32,361.45 (25,105.35, 41,856.15)	21.91 (17.17, 28.45)	1.5 (1.2, 1.8)	33,826.81 (28,763.84, 40,416.20)	4.88 (4.38, 5.31)	-0.4 (-0.6, -0.1)	189,021.06 (170,843.40, 207,065.07)	131.29 (120.84, 142.41)	-1.0 (-1.1, -0.9)
Ghana	850.97 (614.84, 1,119.34)	3.58 (2.74, 4.58)	-0.8 (-1.0, -0.6)	4.42 (2.53, 6.52)	2.91 (2.30, 3.67)	-0.9 (-1.0, -0.8)	29,581.16 (20,674.22, 39,425.95)	106.22 (78.54, 138.08)	-1.3 (-1.5, -1.1)

Greece	3,269.06 (2,598.50, 4,044.78)	17.73 (13.86, 22.23)	1.1 (0.6, 1.5)	156.88 (100.77, 242.87)	6.59 (5.95, 7.05)	-0.2 (-0.7, 0.2)	29,842.19 (27,180.98 , 32,142.11)	168.83 (155.89, 181.27)	-0.5 (-0.7, -0.4)
Greenland	2.88 (2.30, 3.57)	4.43 (3.56, 5.51)	-0.7 (-1.0, -0.5)	139.08 (122.26, 158.06)	3.20 (2.57, 3.94)	-1.3 (-1.5, -1.0)	54.95 (43.08, 69.10)	86.03 (67.38, 108.77)	-1.6 (-2.0, -1.1)
Grenada	6.25 (5.54, 6.97)	5.99 (5.31, 6.73)	-0.9 (-1.1, -0.6)	248.17 (171.96, 336.86)	4.28 (3.84, 4.73)	-1.0 (-1.2, -0.8)	157.19 (137.25, 176.72)	149.56 (131.65, 168.13)	-1.3 (-1.6, -1.0)
Guam	8.82 (7.12, 10.70)	5.01 (4.06, 6.05)	-0.8 (-1.2, -0.3)	407.95 (323.81, 507.42)	3.34 (2.72, 4.08)	-1.2 (-1.8, -0.7)	219.85 (178.47, 270.25)	126.31 (102.61, 155.09)	-0.9 (-1.1, -0.7)
Guatemala	1,119.44 (869.52, 1,405.79)	6.91 (5.40, 8.64)	0.6 (0.1, 1.0)	78.88 (59.57, 102.82)	4.89 (3.80, 6.10)	0.9 (0.4, 1.3)	37,100.24 (28,869.07 , 46,534.89)	211.05 (164.15, 264.20)	0.6 (0.1, 1.2)
Guinea	318.41 (232.02, 425.58)	3.34 (2.51, 4.25)	0.1 (-0.1, 0.3)	287.24 (198.92, 416.41)	2.80 (2.11, 3.53)	0.1 (0.0, 0.2)	11,884.36 (8,479.52, 16,120.36)	103.63 (76.48, 133.59)	-0.1 (-0.3, 0.2)
Guinea-Bissau	67.03 (48.66, 92.62)	5.02 (3.74, 6.78)	-0.3 (-0.4, -0.1)	270.24 (220.65, 324.15)	4.09 (3.07, 5.44)	-0.2 (-0.3, -0.1)	2,401.17 (1,737.96, 3,306.08)	150.17 (111.08, 201.49)	-0.5 (-0.8, -0.3)
Guyana	34.91 (26.81, 44.81)	4.90 (3.78, 6.25)	-0.2 (-0.5, 0.2)	185.87 (144.46, 232.82)	3.87 (3.01, 4.89)	-0.2 (-0.5, 0.1)	1,173.81 (902.95, 1,510.70)	156.01 (120.56, 200.30)	0.0 (-0.6, 0.6)

Haiti	1,087.82 (544.28, 1,858.56)	9.40 (5.14, 15.21)	-1.1 (-1.3, -1.0)	5,265.41 (2,912.09, 7,253.87)	6.40 (4.09, 9.58)	-0.9 (-1.0, -0.8)	36,836.38 (20,218.80, 61,572.02)	294.11 (168.51, 475.78)	-1.3 (-1.4, -1.1)
Honduras	606.94 (448.72, 801.15)	7.83 (6.06, 10.01)	-0.5 (-0.6, -0.5)	21.10 (17.01, 25.61)	6.28 (4.83, 7.97)	-0.1 (-0.3, 0.2)	18,641.69 (13,386.50, 24,840.67)	216.06 (161.02, 281.01)	-1.2 (-1.3, -1.1)
Hungary	1,720.33 (1,410.27, 2,061.21)	10.52 (8.62, 12.70)	0.4 (0.0, 0.8)	228.05 (169.14, 292.58)	5.27 (4.40, 6.28)	-1.1 (-1.3, -1.0)	22,037.23 (18,200.75, 26,442.30)	152.94 (126.73, 184.02)	-1.4 (-1.7, -1.1)
Iceland	57.41 (48.49, 67.04)	12.40 (10.49, 14.46)	0.3 (0.2, 0.5)	1,334.28 (1,099.03, 1,611.25)	3.67 (3.17, 4.16)	-0.8 (-1.0, -0.6)	453.81 (396.81, 512.82)	101.56 (88.27, 115.57)	-1.1 (-1.4, -0.9)
India	43,570.33 (37,235.04, 52,165.15)	3.56 (3.03, 4.25)	-0.9 (-1.2, -0.6)	266.10 (204.10, 347.81)	2.87 (2.44, 3.42)	-0.9 (-1.3, -0.5)	1,312,881.13 (1,115,465.62, 1,600,749.10)	100.48 (85.30, 122.30)	-1.5 (-1.9, -1.1)
Indonesia	15,268.89 (12,252.32, 18,468.14)	6.71 (5.42, 8.08)	-1.0 (-1.0, -0.9)	633.09 (562.67, 696.24)	4.93 (3.99, 5.95)	-0.5 (-0.5, -0.4)	437,885.09 (351,929.03,	180.52 (145.51, 220.76)	-1.3 (-1.3, -1.2)

							538,978.86)		
Iran (Islamic Republic of)	6,766.76 (4,646.38, 7,890.11)	8.85 (6.17, 10.29)	-1.0 (-1.3, -0.7)	380.84 (344.27, 409.44)	6.04 (4.31, 6.85)	-1.1 (-1.3, -1.0)	162,850.27 (110,681.46, 188,806.16)	205.30 (140.29, 237.81)	-1.6 (-1.8, -1.4)
Iraq	3,175.25 (2,441.22, 3,968.43)	9.89 (7.69, 12.30)	-0.8 (-1.0, -0.5)	135.50 (86.62, 209.51)	6.95 (5.40, 8.75)	-0.6 (-0.8, -0.3)	79,420.60 (61,872.41, 100,139.39)	227.60 (176.92, 283.88)	-1.2 (-1.4, -0.9)
Ireland	968.88 (750.66, 1,214.22)	15.06 (11.70, 18.92)	1.4 (0.9, 1.9)	622.18 (545.48, 706.37)	4.25 (3.81, 4.67)	-1.2 (-1.4, -1.0)	7,031.29 (6,350.22, 7,727.06)	109.90 (99.90, 120.52)	-1.4 (-1.7, -1.1)
Israel	1,611.78 (1,250.96, 2,026.82)	14.78 (11.37, 18.70)	1.1 (0.8, 1.4)	1,290.24 (1,012.10, 1,656.11)	6.24 (5.50, 6.80)	-0.7 (-0.9, -0.4)	16,371.11 (14,844.69, 17,737.29)	154.42 (141.13, 166.92)	-1.1 (-1.4, -0.8)
Italy	20,460.65 (17,021.34, 24,398.78)	20.87 (17.47, 24.82)	0.9 (0.7, 1.1)	5.85 (5.10, 6.73)	5.10 (4.61, 5.38)	-0.9 (-1.0, -0.8)	142,984.84 (129,985.22,	146.68 (137.03, 155.08)	-1.4 (-1.7, -1.2)

							151,720.86)		
Jamaica	183.85 (142.70, 231.91)	6.36 (4.94, 8.03)	1.2 (0.3, 2.2)	8.14 (6.87, 9.52)	4.45 (3.47, 5.57)	1.4 (0.4, 2.5)	4,848.27 (3,753.67, 6,180.31)	170.67 (132.24, 217.06)	1.1 (-0.3, 2.5)
Japan	22,630.29 (19,076.10, 26,390.13)	10.81 (9.34, 12.49)	0.5 (0.4, 0.7)	283.37 (256.80, 312.02)	3.09 (2.79, 3.25)	-1.3 (-1.4, -1.1)	200,775.57 (181,948.22, 211,813.40)	97.52 (92.15, 102.16)	-1.9 (-2.0, -1.8)
Jordan	868.92 (708.22, 1,074.47)	9.86 (7.99, 11.92)	-0.6 (-0.7, -0.4)	378.33 (274.73, 483.75)	6.62 (5.29, 7.99)	-0.9 (-1.3, -0.5)	17,937.39 (14,616.00, 21,885.12)	191.83 (155.84, 229.55)	-1.3 (-1.5, -1.1)
Kazakhstan	978.09 (854.43, 1,116.24)	5.45 (4.77, 6.19)	-1.2 (-1.4, -0.9)	454.07 (365.39, 541.70)	3.56 (3.13, 4.04)	-1.2 (-1.5, -1.0)	25,578.25 (22,415.09, 29,104.04)	140.32 (123.18, 159.52)	-1.9 (-2.1, -1.6)
Kenya	1,276.92 (931.84, 1,678.85)	3.25 (2.51, 4.15)	-0.4 (-0.7, -0.1)	138.69 (106.26, 171.62)	2.53 (1.97, 3.19)	0.1 (-0.1, 0.3)	42,338.15 (30,049.00, 55,757.66)	95.22 (70.82, 122.24)	-0.4 (-0.7, -0.1)

Kiribati	5.99 (4.52, 8.02)	5.92 (4.57, 7.48)	-0.5 (-0.7, -0.3)	4,424.32 (3,137.38, 5,029.79)	4.43 (3.51, 5.63)	-0.5 (-0.6, -0.3)	188.62 (142.08, 250.69)	171.26 (131.59, 219.89)	-0.6 (-0.9, -0.4)
Kuwait	218.40 (182.10, 263.92)	6.88 (5.68, 8.39)	-0.7 (-1.6, 0.3)	47.94 (36.94, 60.73)	3.62 (3.01, 4.39)	-1.6 (-2.8, -0.4)	3,752.15 (3,150.75, 4,501.22)	110.42 (92.39, 133.64)	-1.9 (-2.6, -1.2)
Kyrgyzstan	227.63 (200.43, 259.80)	3.94 (3.47, 4.49)	-2.2 (-2.5, -2.0)	63.92 (44.42, 86.13)	2.85 (2.50, 3.24)	-1.6 (-2.0, -1.3)	7,122.94 (6,267.21, 8,061.15)	113.99 (100.39, 129.02)	-2.5 (-2.8, -2.2)
Lao People's Democratic Republic	468.07 (328.39, 625.31)	7.64 (5.48, 9.95)	-1.9 (-2.1, -1.7)	2.43 (1.90, 2.97)	5.50 (4.09, 7.01)	-1.6 (-1.7, -1.5)	14,270.60 (9,966.87, 18,907.18)	214.01 (153.26, 280.80)	-2.2 (-2.4, -2.0)
Latvia	297.87 (243.05, 362.60)	9.37 (7.70, 11.35)	-0.6 (-1.3, 0.2)	26.38 (20.31, 33.63)	5.02 (4.13, 6.05)	-1.4 (-2.2, -0.5)	4,322.74 (3,556.57, 5,224.06)	162.15 (132.78, 196.97)	-2.1 (-2.8, -1.3)
Lebanon	622.37 (494.32, 791.48)	12.06 (9.60, 15.26)	0.5 (0.4, 0.6)	3.78 (2.91, 4.79)	6.52 (5.15, 8.53)	-1.0 (-1.1, -0.9)	10,497.59 (8,232.37, 13,119.50)	203.88 (160.37, 254.39)	-1.1 (-1.1, -1.0)
Lesotho	84.46 (58.76, 116.02)	5.40 (3.81, 7.35)	1.5 (1.4, 1.6)	285.12 (261.26, 303.31)	4.53 (3.18, 6.11)	1.4 (1.3, 1.6)	2,550.01 (1,751.07, 3,555.00)	145.15 (100.02, 199.82)	1.4 (1.3, 1.5)
Liberia	133.70 (90.50, 183.23)	3.86 (2.65, 5.20)	-0.8 (-1.0, -0.5)	161.86 (118.86, 213.82)	3.15 (2.18, 4.23)	-0.5 (-0.7, -0.3)	4,680.27 (3,142.13, 6,391.17)	113.35 (77.85, 153.04)	-1.1 (-1.4, -0.8)

Libya	387.77 (304.26, 498.88)	6.92 (5.45, 8.74)	-0.5 (-0.8, -0.3)	56.66 (38.62, 81.47)	5.18 (4.00, 6.49)	-0.6 (-1.0, -0.3)	10,282.91 (7,998.55, 13,282.17)	168.55 (131.72, 214.29)	-0.9 (-1.2, -0.6)
Lithuania	406.98 (332.11, 495.69)	8.50 (7.00, 10.21)	-0.8 (-1.4, -0.1)	92.36 (63.59, 124.08)	5.13 (4.24, 6.13)	-1.2 (-2.0, -0.4)	6,293.76 (5,154.53, 7,572.66)	157.12 (130.46, 187.50)	-2.0 (-2.8, -1.2)
Luxembourg	115.35 (95.11, 138.86)	13.99 (11.53, 16.86)	0.0 (-0.3, 0.3)	3,451.10 (2,930.51, 4,041.66)	5.46 (4.78, 6.25)	-1.3 (-1.5, -1.1)	1,189.36 (1,040.87, 1,360.40)	148.27 (129.07, 171.25)	-1.8 (-2.0, -1.6)
Madagascar	816.85 (553.60, 1,154.97)	3.55 (2.56, 4.77)	-1.1 (-1.3, -0.9)	106.63 (78.70, 141.24)	2.63 (1.95, 3.48)	-0.6 (-0.7, -0.5)	27,443.17 (18,745.62 , 39,068.20)	105.45 (75.60, 141.53)	-1.3 (-1.5, -1.1)
Malawi	528.07 (340.06, 832.60)	3.49 (2.46, 5.03)	-1.0 (-1.3, -0.7)	20.74 (12.14, 32.41)	2.55 (1.86, 3.46)	-0.6 (-0.8, -0.4)	17,748.58 (11,185.26 , 28,351.91)	102.30 (68.87, 153.47)	-1.2 (-1.6, -0.8)
Malaysia	2,002.97 (1,568.44, 2,612.67)	7.05 (5.52, 9.18)	-0.4 (-0.5, -0.3)	946.78 (787.53, 1,132.30)	5.10 (4.01, 6.64)	-0.4 (-0.6, -0.2)	54,662.12 (42,791.40 , 69,727.80)	180.99 (141.59, 232.52)	-0.9 (-1.2, -0.6)
Maldives	20.43 (16.50, 25.05)	5.51 (4.52, 6.61)	-1.7 (-2.0, -1.4)	438.23 (313.42, 622.46)	3.51 (2.86, 4.17)	-2.1 (-2.2, -1.9)	491.41 (396.86, 633.51)	118.38 (97.05, 146.06)	-2.6 (-2.8, -2.3)

Mali	569.44 (404.31, 812.27)	3.26 (2.44, 4.35)	-0.2 (-0.5, 0.2)	213.70 (141.00, 314.52)	2.62 (1.99, 3.43)	-0.2 (-0.5, 0.1)	21,066.79 (14,533.29 , 31,164.35)	99.78 (72.45, 136.89)	-0.4 (-0.8, 0.0)
Malta	81.54 (67.63, 97.18)	12.29 (10.26, 14.65)	1.4 (1.2, 1.7)	20.02 (17.17, 22.80)	3.86 (3.34, 4.38)	-1.1 (-1.2, -1.0)	726.22 (634.96, 822.63)	116.95 (102.36, 131.47)	-0.9 (-1.0, -0.8)
Marshall Islands	3.38 (2.30, 4.82)	7.31 (5.08, 10.33)	-0.2 (-0.2, -0.1)	7,697.52 (6,499.14, 8,584.48)	5.77 (4.09, 8.12)	-0.4 (-0.6, -0.2)	109.27 (74.81, 156.80)	210.32 (144.75, 299.86)	-0.3 (-0.3, -0.2)
Mauritania	104.45 (67.12, 154.09)	3.53 (2.41, 4.88)	-0.5 (-0.8, -0.3)	7.31 (5.35, 9.51)	2.89 (2.05, 3.82)	-0.7 (-0.9, -0.5)	3,342.98 (2,069.43, 4,971.92)	96.98 (63.45, 138.51)	-0.9 (-1.2, -0.7)
Mauritius	75.34 (61.02, 91.26)	5.84 (4.73, 7.09)	-0.5 (-0.9, -0.1)	345.67 (250.51, 463.42)	3.39 (2.79, 4.07)	-0.2 (-0.6, 0.1)	1,827.76 (1,493.63, 2,217.66)	141.89 (116.02, 171.18)	-0.4 (-0.7, -0.1)
Mexico	7,787.37 (6,810.46, 8,824.89)	6.44 (5.63, 7.28)	-0.2 (-0.4, 0.1)	28.38 (19.45, 38.69)	4.37 (3.84, 4.95)	-0.1 (-0.2, 0.0)	238,811.95 (210,172.64, 268,748.31)	194.13 (170.74, 217.88)	-0.3 (-0.6, 0.0)
Micronesia (Federated States of)	6.24 (3.31, 9.02)	7.27 (4.23, 10.41)	-0.5 (-0.5, -0.4)	5,857.10 (5,316.65, 6,138.89)	5.78 (3.65, 8.28)	-0.5 (-0.6, -0.5)	193.72 (99.37, 284.17)	204.27 (109.23, 298.40)	-0.7 (-0.7, -0.6)

Monaco	17.67 (13.13, 22.36)	27.38 (19.66, 36.33)	0.7 (0.5, 0.8)	44.92 (34.58, 58.32)	9.66 (7.42, 11.85)	0.2 (0.1, 0.2)	171.55 (132.42, 211.42)	273.49 (215.35, 341.21)	-0.3 (-0.4, -0.3)
Mongolia	111.20 (83.08, 148.77)	3.79 (2.86, 4.96)	-0.1 (-0.4, 0.1)	1,876.10 (1,453.28, 2,350.53)	2.88 (2.20, 3.74)	-0.2 (-0.4, 0.1)	3,635.76 (2,686.49, 4,938.27)	114.00 (85.40, 151.89)	-0.5 (-0.8, -0.3)
Montenegro	86.92 (70.83, 105.62)	10.03 (8.16, 12.21)	1.3 (0.9, 1.6)	576.77 (513.17, 641.06)	4.58 (3.77, 5.52)	0.0 (-0.4, 0.4)	1,134.40 (924.38, 1,372.42)	144.73 (115.67, 177.20)	-0.6 (-1.0, -0.2)
Morocco	992.35 (752.74, 1,265.34)	3.06 (2.37, 3.86)	-0.1 (-0.3, 0.1)	194.00 (151.65, 247.74)	2.47 (1.91, 3.10)	0.0 (-0.3, 0.3)	26,472.12 (19,583.69, 34,258.74)	77.29 (57.99, 98.82)	-0.5 (-0.7, -0.2)
Mozambique	1,953.50 (1,093.74, 3,576.30)	6.33 (4.29, 9.68)	-0.1 (-0.4, 0.3)	4,404.62 (3,489.54, 5,641.97)	4.32 (3.22, 5.69)	0.1 (-0.1, 0.3)	61,585.48 (34,067.76, 111,060.74)	180.87 (118.93, 283.85)	-0.3 (-0.7, 0.1)
Myanmar	4,219.39 (2,929.94, 5,833.45)	8.37 (5.93, 11.44)	-1.4 (-1.5, -1.3)	397.90 (287.45, 546.31)	5.53 (4.19, 7.15)	-1.3 (-1.4, -1.3)	118,829.91 (82,099.35, 164,511.81)	225.56 (157.10, 311.15)	-1.8 (-1.9, -1.7)

Namibia	59.84 (41.99, 79.80)	3.33 (2.45, 4.35)	0.6 (0.3, 0.9)	32.56 (27.94, 37.30)	2.53 (1.95, 3.23)	0.3 (0.0, 0.6)	1,620.44 (1,109.40, 2,170.78)	80.88 (57.28, 107.56)	0.2 (-0.1, 0.4)
Nauru	0.76 (0.50, 1.11)	9.07 (6.39, 12.74)	-0.4 (-0.5, -0.3)	2.64 (2.03, 3.37)	6.46 (4.62, 8.80)	-0.5 (-0.6, -0.4)	22.84 (15.15, 33.38)	242.73 (166.99, 346.34)	-0.6 (-0.7, -0.5)
Nepal	891.35 (681.67, 1,119.57)	3.59 (2.78, 4.49)	-0.9 (-1.0, -0.8)	671.27 (513.12, 847.43)	3.08 (2.36, 3.88)	-0.6 (-0.7, -0.5)	26,878.14 (20,742.25, 34,105.52)	98.37 (76.25, 124.12)	-1.6 (-1.8, -1.4)
Netherlands	3,966.56 (3,109.72, 5,002.07)	15.30 (11.93, 19.31)	0.7 (-0.4, 1.8)	1,312.36 (979.60, 1,725.29)	5.43 (4.87, 5.90)	0.1 (-0.4, 0.5)	36,162.89 (32,935.48, 39,252.83)	134.00 (123.11, 144.95)	-0.8 (-1.2, -0.4)
New Zealand	834.48 (694.96, 986.05)	12.46 (10.50, 14.66)	0.4 (0.1, 0.7)	40.97 (33.59, 49.20)	5.05 (4.63, 5.40)	-0.7 (-0.8, -0.6)	8,439.80 (7,867.49, 8,957.80)	137.31 (129.60, 144.69)	-1.1 (-1.2, -0.9)
Nicaragua	342.47 (261.33, 428.21)	6.06 (4.76, 7.44)	0.1 (-0.4, 0.6)	57.17 (43.68, 73.70)	4.41 (3.53, 5.33)	0.3 (-0.2, 0.8)	10,862.17 (7,819.21, 13,893.57)	174.41 (129.13, 220.56)	-0.5 (-0.8, -0.2)
Niger	941.14 (534.26, 1,571.07)	4.43 (3.00, 6.33)	-0.1 (-0.3, 0.2)	1,847.09 (1,377.08, 2,430.70)	3.32 (2.31, 4.50)	0.0 (-0.3, 0.3)	31,494.19 (18,644.79, 53,766.76)	129.05 (85.74, 188.43)	-0.3 (-0.7, 0.0)

Nigeria	5,199.82 (3,704.43, 6,947.04)	3.46 (2.70, 4.39)	0.0 (-0.1, 0.1)	100.51 (71.56, 121.83)	2.87 (2.24, 3.58)	-0.1 (-0.2, 0.1)	177,759.98 (123,076.58, 250,446.36)	96.97 (72.25, 128.36)	-0.3 (-0.5, -0.1)
Niue	0.12 (0.09, 0.16)	6.58 (4.64, 8.66)	-0.3 (-0.3, -0.2)	30.09 (19.22, 49.17)	4.40 (3.17, 5.80)	-0.7 (-0.7, -0.6)	2.86 (2.00, 3.83)	162.66 (111.96, 221.55)	-0.7 (-0.7, -0.6)
North Macedonia	240.48 (180.92, 307.26)	8.61 (6.43, 10.96)	0.8 (0.6, 0.9)	638.67 (380.53, 994.02)	4.57 (3.55, 5.73)	-0.1 (-0.2, 0.0)	3,994.31 (2,924.59, 5,109.44)	154.10 (111.29, 197.31)	-0.5 (-0.8, -0.2)
Northern Mariana Islands	2.82 (2.28, 3.52)	6.29 (5.09, 7.86)	-1.2 (-1.5, -0.9)	930.61 (841.87, 994.14)	4.39 (3.65, 5.38)	-1.7 (-1.8, -1.5)	67.51 (54.24, 86.21)	146.35 (118.60, 187.16)	-1.7 (-1.8, -1.6)
Norway	691.07 (588.58, 808.46)	8.73 (7.45, 10.13)	0.2 (0.0, 0.4)	337.70 (265.98, 441.68)	3.07 (2.84, 3.25)	-0.8 (-0.9, -0.7)	6,289.78 (5,901.18, 6,679.06)	84.02 (79.07, 89.30)	-1.5 (-1.8, -1.3)
Oman	165.51 (123.64, 209.10)	6.49 (5.18, 7.70)	0.0 (-0.2, 0.2)	1,155.44 (890.79, 1,492.83)	4.45 (3.65, 5.19)	-0.6 (-0.8, -0.4)	4,026.37 (2,989.86, 5,036.66)	128.85 (101.38, 153.55)	-1.0 (-1.2, -0.8)
Pakistan	10,833.15 (8,450.83, 13,875.10)	5.48 (4.49, 6.77)	0.3 (0.1, 0.5)	144.42 (118.49, 177.02)	3.94 (3.28, 4.72)	0.2 (0.0, 0.3)	363,564.55 (290,877.48,	164.17 (134.44, 198.89)	0.2 (0.0, 0.3)

							449,773.54)		
Palau	0.58 (0.43, 0.75)	2.97 (2.28, 3.72)	-0.2 (-0.3, -0.1)	177.69 (145.49, 213.59)	2.28 (1.72, 2.85)	-0.4 (-0.5, -0.4)	14.64 (11.05, 18.61)	73.42 (56.55, 91.37)	-0.5 (-0.6, -0.4)
Palestine	317.97 (265.61, 391.25)	9.52 (8.05, 11.28)	-1.0 (-1.3, -0.7)	773.89 (580.40, 1,053.05)	7.27 (6.07, 8.56)	-0.9 (-1.1, -0.7)	7,499.91 (6,317.55, 9,165.70)	203.13 (170.77, 240.13)	-1.2 (-1.4, -1.1)
Panama	271.47 (208.40, 342.21)	6.53 (5.02, 8.22)	0.3 (0.1, 0.5)	467.03 (334.30, 628.98)	4.46 (3.47, 5.59)	-0.2 (-0.4, 0.0)	7,541.84 (5,802.09, 9,526.08)	181.64 (139.65, 229.35)	-0.3 (-0.6, -0.1)
Papua New Guinea	591.96 (356.87, 954.73)	6.59 (4.30, 9.92)	0.0 (-0.4, 0.3)	2,120.31 (1,410.19, 3,185.10)	4.50 (3.15, 6.45)	-0.1 (-0.4, 0.2)	17,953.50 (11,669.59, 28,126.08)	185.10 (125.31, 275.56)	-0.3 (-0.6, 0.1)
Paraguay	377.48 (284.84, 493.78)	6.07 (4.58, 7.85)	-0.1 (-0.4, 0.2)	776.53 (605.64, 970.46)	4.52 (3.43, 5.84)	0.2 (-0.2, 0.6)	10,704.94 (8,104.96, 13,923.19)	161.69 (122.22, 210.70)	-0.4 (-0.8, 0.0)
Peru	2,184.62 (1,452.56, 3,034.11)	6.60 (4.38, 9.16)	-0.3 (-0.8, 0.2)	7,152.94 (6,289.66, 7,633.14)	4.40 (3.03, 6.00)	-0.4 (-1.0, 0.3)	60,574.52 (39,891.44, 84,888.77)	180.89 (119.20, 253.41)	-0.8 (-1.3, -0.2)
Philippines	6,953.86 (5,837.66, 8,424.87)	6.79 (5.71, 8.20)	-1.0 (-1.2, -0.9)	253.28 (196.58, 315.92)	4.55 (3.81, 5.42)	-1.2 (-1.3, -1.0)	208,842.81 (175,728.3	191.18 (161.76, 223.59)	-1.1 (-1.3, -0.8)

							0, 244,235.14)		
Poland	6,333.37 (5,327.83, 7,510.62)	10.30 (8.75, 12.17)	0.9 (0.7, 1.1)	0.05 (0.04, 0.07)	5.21 (4.43, 6.07)	-0.6 (-0.7, -0.5)	81,048.19 (69,162.24, 94,508.74)	145.41 (124.73, 167.90)	-1.5 (-1.7, -1.3)
Portugal	2,404.62 (1,865.02, 3,016.09)	13.54 (10.34, 17.03)	1.2 (0.7, 1.6)	6.04 (4.92, 7.39)	4.65 (4.19, 5.03)	-0.9 (-1.3, -0.6)	22,028.06 (19,861.69, 23,912.90)	133.39 (121.96, 145.39)	-1.8 (-2.3, -1.3)
Puerto Rico	374.00 (296.56, 468.67)	6.76 (5.37, 8.56)	-0.1 (-0.2, 0.0)	1,010.90 (777.25, 1,244.49)	4.06 (3.22, 5.08)	-1.1 (-1.2, -1.0)	5,964.93 (4,705.20, 7,505.17)	124.19 (97.87, 156.45)	-1.5 (-1.6, -1.4)
Qatar	127.06 (87.86, 179.53)	13.28 (9.49, 18.27)	1.2 (0.0, 2.4)	53.30 (46.38, 60.94)	8.54 (5.58, 12.24)	0.3 (0.1, 0.6)	2,532.18 (1,714.82, 3,793.10)	172.50 (126.62, 229.30)	-0.9 (-1.7, -0.2)
Republic of Korea	5,983.49 (4,174.11, 7,467.31)	9.19 (6.70, 11.45)	0.6 (0.3, 0.9)	152.19 (134.16, 173.17)	2.69 (2.00, 3.15)	-1.8 (-2.0, -1.7)	60,045.45 (44,776.48, 71,351.70)	96.63 (72.15, 116.25)	-2.7 (-2.9, -2.5)
Republic of Moldova	208.89 (183.83, 234.75)	5.01 (4.39, 5.66)	-2.6 (-3.3, -1.8)	19.58 (13.58, 27.87)	2.90 (2.58, 3.25)	-2.6 (-3.5, -1.7)	4,619.53 (4,094.41, 5,203.01)	118.39 (104.99, 132.69)	-3.1 (-3.4, -2.9)

Romania	2,134.38 (1,753.28, 2,580.27)	7.36 (6.09, 8.78)	0.7 (0.7, 0.8)	3,494.93 (2,543.72, 4,647.07)	4.16 (3.43, 4.97)	0.1 (-0.4, 0.6)	35,614.79 (29,170.12 , 42,804.41)	142.37 (118.98, 169.26)	-1.0 (-1.1, -1.0)
Russian Federation	14,266.01 (12,501.69, 16,317.41)	7.09 (6.29, 8.06)	0.7 (-0.4, 1.7)	438.90 (335.95, 563.43)	3.55 (3.11, 4.03)	-0.8 (-1.5, 0.0)	218,493.3 1 (191,831.4 6, 249,166.8 0)	122.63 (107.65, 138.69)	-1.3 (-2.1, -0.5)
Rwanda	519.94 (330.87, 810.96)	4.67 (3.23, 6.72)	-0.4 (-0.9, 0.0)	929.05 (590.40, 1,510.34)	3.25 (2.42, 4.34)	-0.5 (-0.9, - 0.2)	16,458.62 (10,474.21 , 26,008.04)	133.26 (90.55, 199.49)	-0.9 (-1.3, -0.4)
Saint Kitts and Nevis	3.73 (2.81, 4.64)	6.22 (4.70, 7.72)	-1.1 (-1.6, - 0.5)	566.69 (384.08, 791.00)	4.06 (3.22, 4.92)	-1.2 (-1.4, - 0.9)	83.26 (59.47, 107.26)	136.94 (99.49, 175.74)	-1.6 (-2.0, -1.1)
Saint Lucia	11.08 (9.27, 13.06)	5.89 (4.89, 6.96)	-0.4 (-0.6, - 0.2)	717.57 (549.61, 898.72)	4.15 (3.48, 4.85)	-0.7 (-1.0, - 0.5)	279.26 (232.37, 329.07)	153.87 (127.26, 182.31)	-0.7 (-0.9, -0.5)
Saint Vincent and the Grenadines	7.87 (6.77, 9.13)	6.50 (5.58, 7.58)	-0.4 (-0.6, - 0.2)	3,325.40 (2,976.70, 3,635.37)	4.72 (4.11, 5.43)	-0.3 (-0.4, - 0.2)	204.51 (174.69, 238.62)	173.08 (147.10, 203.75)	-0.5 (-0.8, -0.3)
Samoa	10.32 (7.24, 13.79)	5.92 (4.26, 7.75)	-0.5 (-0.6, - 0.4)	5.15 (4.16, 6.32)	4.63 (3.42, 5.97)	-0.5 (-0.5, - 0.5)	282.93 (194.72, 383.38)	152.23 (106.93, 203.33)	-0.6 (-0.7, -0.6)

San Marino	15.94 (12.09, 21.33)	35.14 (26.37, 47.19)	1.6 (1.5, 1.7)	1,647.37 (1,140.13, 2,421.00)	8.22 (5.48, 11.55)	-0.2 (-0.3, -0.1)	109.39 (73.41, 155.43)	230.29 (156.84, 327.24)	-0.3 (-0.4, -0.3)
Sao Tome and Principe	5.43 (3.77, 7.68)	3.58 (2.58, 4.80)	0.2 (0.0, 0.3)	1,452.61 (997.53, 1,984.69)	2.82 (2.08, 3.67)	0.2 (0.0, 0.5)	174.63 (121.82, 248.10)	100.53 (71.87, 136.25)	-0.3 (-0.5, -0.1)
Saudi Arabia	1,378.55 (1,018.38, 1,896.26)	5.36 (4.11, 7.08)	0.7 (0.6, 0.8)	616.28 (451.39, 787.17)	3.43 (2.67, 4.59)	-0.8 (-0.8, -0.7)	33,448.69 (24,936.13, 45,749.00)	107.27 (82.58, 141.54)	-0.9 (-1.0, -0.8)
Senegal	509.49 (348.67, 684.32)	4.37 (3.13, 5.69)	0.1 (-0.7, 0.9)	388.79 (268.67, 539.21)	3.54 (2.58, 4.55)	0.1 (-0.5, 0.7)	17,246.43 (11,526.97, 23,857.04)	127.35 (88.50, 171.31)	-0.2 (-1.2, 0.9)
Serbia	1,067.56 (812.94, 1,356.98)	7.92 (5.95, 9.91)	-0.1 (-0.7, 0.5)	1,685.29 (1,126.95, 2,346.12)	4.93 (3.81, 6.16)	-0.3 (-0.6, 0.0)	17,917.78 (13,637.46, 22,748.40)	142.07 (106.73, 177.95)	-1.2 (-1.8, -0.7)
Seychelles	9.91 (8.54, 11.69)	9.34 (8.06, 10.95)	0.2 (-0.3, 0.7)	1,498.04 (1,063.85, 1,983.64)	6.36 (5.48, 7.36)	-0.3 (-0.7, 0.1)	229.99 (197.44, 271.18)	219.06 (186.64, 260.91)	-0.4 (-1.2, 0.3)
Sierra Leone	358.94 (221.45, 571.96)	4.89 (3.29, 7.11)	0.4 (0.2, 0.7)	597.80 (394.95, 867.48)	3.60 (2.55, 4.96)	0.3 (0.2, 0.5)	12,539.17 (7,608.19, 20,063.14)	149.82 (98.35, 220.85)	0.2 (0.0, 0.5)

Singapore	548.78 (425.14, 685.85)	8.70 (6.82, 10.90)	0.9 (0.6, 1.3)	0.53 (0.42, 0.65)	2.75 (2.49, 3.01)	-1.7 (-2.2, -1.2)	5,662.89 (5,162.44, 6,197.89)	93.82 (85.02, 103.70)	-2.3 (-2.7, -1.9)
Slovakia	751.22 (566.78, 978.09)	9.49 (7.23, 12.29)	0.8 (0.5, 1.1)	724.16 (562.52, 905.50)	4.46 (3.29, 5.68)	-0.8 (-1.1, -0.5)	9,759.40 (7,268.41, 12,554.28)	135.10 (101.55, 173.11)	-1.1 (-1.3, -0.8)
Slovenia	466.16 (356.01, 629.82)	12.92 (9.96, 17.54)	1.5 (1.4, 1.6)	96.44 (80.79, 115.89)	4.58 (3.59, 5.85)	-0.7 (-0.7, -0.6)	4,052.09 (3,165.38, 5,204.01)	121.97 (96.76, 154.07)	-1.1 (-1.3, -0.9)
Solomon Islands	47.43 (30.32, 77.46)	9.03 (6.07, 14.31)	0.0 (-0.2, 0.2)	4.49 (4.01, 4.97)	6.83 (4.66, 10.68)	-0.1 (-0.3, 0.0)	1,542.70 (964.60, 2,564.56)	266.25 (170.17, 435.50)	-0.1 (-0.3, 0.1)
Somalia	1,005.79 (573.61, 1,573.70)	4.82 (2.92, 7.05)	-0.2 (-0.4, -0.1)	315.02 (213.49, 471.32)	3.49 (2.24, 4.90)	0.0 (-0.1, 0.1)	34,492.54 (21,238.00, 51,367.68)	149.58 (96.56, 212.99)	-0.3 (-0.4, -0.1)
South Africa	2,092.00 (1,780.09, 2,373.49)	4.37 (3.66, 4.94)	-0.3 (-0.5, -0.1)	49.88 (40.76, 60.25)	3.34 (2.66, 3.76)	-0.2 (-0.6, 0.1)	51,063.94 (43,169.27, 59,229.35)	99.42 (83.20, 113.33)	-1.0 (-1.5, -0.5)
South Sudan	514.59 (345.42, 729.92)	5.05 (3.64, 6.85)	-0.7 (-0.9, -0.5)	657.44 (535.58, 802.16)	3.34 (2.38, 4.56)	-0.4 (-0.5, -0.2)	16,573.48 (11,179.44, 23,098.80)	148.89 (104.37, 203.18)	-0.8 (-1.0, -0.6)

Spain	10,957.62 (8,615.90, 13,779.35)	16.10 (12.74, 20.33)	1.1 (1.0, 1.2)	5,249.30 (4,602.84, 5,958.00)	4.42 (4.01, 4.78)	-0.8 (-0.9, -0.7)	85,450.64 (77,930.84, 92,576.13)	127.21 (117.29, 137.62)	-1.6 (-1.7, -1.5)
Sri Lanka	1,303.42 (931.67, 1,725.32)	5.75 (4.08, 7.65)	-0.6 (-1.0, -0.2)	616.34 (462.53, 795.72)	3.84 (2.79, 4.99)	-0.9 (-1.1, -0.6)	31,619.02 (22,328.72, 42,012.52)	140.24 (99.67, 185.72)	-1.4 (-2.0, -0.7)
Sudan	2,837.20 (1,891.69, 4,044.63)	8.76 (5.90, 12.03)	-0.6 (-0.7, -0.5)	7,426.66 (6,453.86, 8,467.60)	6.44 (4.40, 8.69)	-0.3 (-0.4, -0.2)	85,335.60 (55,673.50, 122,200.59)	234.93 (155.79, 329.68)	-0.8 (-0.9, -0.7)
Suriname	29.61 (23.57, 36.40)	5.15 (4.11, 6.30)	0.0 (-0.8, 0.7)	74.89 (51.02, 104.66)	3.65 (2.95, 4.43)	0.0 (-0.5, 0.5)	827.30 (660.28, 1,012.42)	143.50 (114.88, 175.81)	-0.2 (-1.2, 0.8)
Sweden	2,257.11 (1,921.20, 2,622.84)	13.62 (11.65, 15.74)	0.3 (0.1, 0.5)	49.92 (34.03, 63.35)	4.32 (3.98, 4.58)	-0.5 (-0.6, -0.4)	17,821.81 (16,608.46, 18,873.06)	111.92 (105.30, 118.13)	-1.2 (-1.3, -1.1)
Switzerland	1,778.78 (1,391.82, 2,246.88)	13.30 (10.42, 17.08)	-1.0 (-1.3, -0.7)	3,391.35 (2,575.12, 4,278.55)	3.69 (3.34, 4.01)	-2.0 (-2.3, -1.8)	13,148.78 (12,000.84, 14,401.99)	100.94 (92.72, 110.84)	-2.8 (-3.1, -2.6)

Syrian Arab Republic	2,664.86 (2,013.59, 3,501.08)	20.88 (15.92, 27.37)	-0.9 (-1.3, -0.4)	0.41 (0.28, 0.60)	15.82 (11.96, 20.42)	-1.1 (-1.2, -1.0)	62,403.61 (47,499.23, 83,969.14)	456.25 (348.86, 606.34)	-1.5 (-1.7, -1.2)
Taiwan (Province of China)	2,674.68 (2,055.97, 3,447.15)	9.26 (7.25, 11.86)	0.5 (0.1, 1.0)	4,212.96 (3,538.75, 4,971.20)	3.76 (2.99, 4.79)	-0.2 (-0.5, 0.2)	35,884.83 (28,335.39, 46,255.57)	129.50 (103.23, 164.03)	-0.9 (-1.4, -0.4)
Tajikistan	421.28 (313.07, 623.71)	5.27 (4.14, 7.24)	-1.6 (-1.9, -1.3)	45.72 (33.88, 61.39)	3.82 (3.09, 4.91)	-1.1 (-1.3, -0.9)	13,822.96 (10,228.55, 20,367.49)	154.51 (118.30, 217.70)	-1.9 (-2.3, -1.5)
Thailand	6,078.42 (3,951.43, 8,200.56)	8.04 (5.46, 10.46)	-0.3 (-0.4, -0.1)	2,950.35 (2,499.05, 3,532.42)	4.69 (2.95, 6.35)	-0.4 (-0.6, -0.3)	129,916.31 (84,446.93, 174,809.84)	171.17 (119.67, 224.21)	-0.8 (-1.0, -0.7)
Timor-Leste	82.64 (50.63, 107.15)	7.01 (4.67, 9.04)	-1.1 (-1.4, -0.9)	5.31 (3.56, 7.30)	5.04 (3.64, 6.42)	-0.6 (-0.9, -0.3)	2,368.15 (1,431.52, 3,086.76)	186.29 (117.62, 239.52)	-1.2 (-1.7, -0.7)
Togo	236.74 (174.53, 315.23)	4.02 (2.98, 5.28)	0.2 (-0.1, 0.4)	0.43 (0.32, 0.56)	3.23 (2.40, 4.22)	0.2 (-0.1, 0.4)	8,056.53 (5,765.60, 10,730.78)	116.63 (85.14, 154.06)	0.0 (-0.3, 0.3)

Tokelau	0.07 (0.05, 0.10)	5.34 (3.68, 7.34)	-0.8 (-1.0, -0.7)	1,071.96 (950.72, 1,176.03)	4.09 (2.88, 5.62)	-0.9 (-0.9, -0.9)	1.93 (1.32, 2.72)	141.83 (95.97, 201.22)	-1.1 (-1.2, -1.0)
Tonga	3.64 (2.74, 4.78)	3.97 (3.01, 5.14)	-0.1 (-0.2, 0.1)	297.78 (216.38, 385.96)	3.09 (2.39, 3.93)	-0.1 (-0.3, 0.0)	102.16 (75.95, 136.31)	106.72 (79.93, 140.62)	-0.1 (-0.3, 0.0)
Trinidad and Tobago	75.58 (56.69, 97.69)	5.02 (3.80, 6.50)	-0.9 (-1.1, -0.8)	6,491.61 (5,335.97, 7,817.44)	3.61 (2.75, 4.65)	-1.2 (-1.4, -1.0)	2,105.02 (1,584.68, 2,729.65)	150.84 (113.33, 194.85)	-1.3 (-1.5, -1.1)
Tunisia	519.59 (373.90, 703.17)	4.38 (3.16, 5.89)	-0.6 (-0.6, -0.5)	11.05 (9.63, 12.51)	2.93 (2.14, 3.92)	-0.8 (-0.9, -0.6)	11,244.74 (8,178.67, 15,076.59)	95.35 (69.88, 127.03)	-1.3 (-1.5, -1.2)
Turkey	6,883.86 (5,515.59, 8,615.53)	8.45 (6.85, 10.48)	-1.3 (-1.4, -1.1)	354.55 (249.42, 471.61)	5.28 (4.21, 6.68)	-1.7 (-1.9, -1.6)	143,270.18 (115,248.33, 176,024.88)	176.71 (143.76, 214.02)	-2.4 (-2.8, -2.1)
Turkmenistan	238.95 (195.76, 293.11)	5.00 (4.12, 6.12)	-1.4 (-2.3, -0.6)	887.02 (640.34, 1,163.63)	3.22 (2.63, 3.97)	-0.8 (-1.4, -0.2)	7,375.76 (6,083.25, 9,026.65)	147.98 (122.05, 180.79)	-1.6 (-2.0, -1.1)
Tuvalu	0.68 (0.46, 0.96)	6.30 (4.23, 8.86)	-1.2 (-1.3, -1.1)	309.84 (217.91, 422.04)	4.91 (3.37, 6.87)	-1.0 (-1.1, -0.9)	20.05 (13.46, 28.50)	175.06 (117.54, 248.92)	-1.4 (-1.5, -1.3)

Uganda	1,423.46 (868.91, 2,246.06)	3.54 (2.49, 5.02)	-0.1 (-0.5, 0.3)	145.44 (107.28, 201.29)	2.40 (1.77, 3.17)	-0.2 (-0.4, 0.0)	42,911.49 (26,346.00, 67,469.48)	98.31 (66.58, 140.90)	-0.5 (-0.8, -0.1)
Ukraine	4,958.75 (4,176.18, 5,859.63)	8.60 (7.33, 10.01)	-1.2 (-2.3, -0.2)	60,380.74 (50,213.16, 70,987.66)	4.78 (4.07, 5.69)	-1.4 (-2.1, -0.7)	96,833.96 (82,600.66, 114,737.47)	194.66 (166.43, 227.72)	-1.8 (-2.9, -0.8)
United Arab Emirates	428.81 (300.13, 592.22)	7.69 (5.21, 10.13)	-1.0 (-1.3, -0.8)	2.33 (1.61, 3.35)	6.56 (4.33, 8.71)	-1.3 (-1.6, -1.0)	13,054.39 (9,155.37, 18,219.56)	177.70 (130.00, 229.86)	-1.2 (-1.7, -0.7)
United Kingdom	12,648.01 (10,622.97, 14,965.18)	12.55 (10.66, 14.72)	0.4 (0.2, 0.6)	7.03 (5.77, 8.29)	4.67 (4.33, 4.87)	-0.6 (-0.7, -0.5)	118,455.78 (111,853.08, 123,402.73)	119.88 (114.88, 124.72)	-1.2 (-1.4, -1.1)
United Republic of Tanzania	3,485.67 (2,200.02, 5,504.43)	5.69 (4.06, 8.19)	0.0 (-0.2, 0.3)	11.75 (9.66, 14.55)	3.65 (2.73, 4.85)	0.1 (0.0, 0.3)	106,405.04 (68,829.54, 166,185.73)	161.63 (112.08, 236.39)	-0.1 (-0.4, 0.1)

United States of America	53,106.99 (45,609.57, 61,436.59)	10.21 (8.88, 11.77)	-0.6 (-0.7, -0.4)	189.32 (170.34, 205.84)	5.74 (5.36, 6.01)	-0.6 (-0.8, -0.4)	668,595.44 (636,491.88, 696,306.42)	145.44 (139.75, 150.82)	-1.1 (-1.3, -1.0)
United States Virgin Islands	9.71 (7.95, 11.57)	6.32 (5.12, 7.68)	-0.1 (-0.3, 0.0)	10.56 (6.99, 15.00)	4.37 (3.62, 5.19)	-0.4 (-0.5, -0.3)	190.16 (154.64, 231.23)	139.32 (112.21, 172.56)	-0.9 (-1.2, -0.6)
Uruguay	373.39 (296.15, 461.17)	7.99 (6.30, 9.96)	-0.4 (-0.6, -0.2)	130.82 (101.69, 163.34)	5.51 (5.06, 6.00)	-0.7 (-0.9, -0.5)	7,029.01 (6,459.30, 7,571.30)	169.89 (156.11, 182.93)	-1.2 (-1.4, -1.0)
Uzbekistan	1,846.06 (1,563.91, 2,177.23)	6.30 (5.40, 7.34)	-1.2 (-1.4, -1.0)	132.13 (101.52, 166.04)	4.24 (3.66, 4.93)	-0.7 (-0.9, -0.4)	56,710.79 (48,230.99, 66,869.92)	175.87 (150.42, 206.78)	-1.4 (-1.6, -1.2)
Vanuatu	15.57 (10.23, 22.03)	6.47 (4.26, 9.15)	0.2 (0.0, 0.4)	272.51 (169.54, 427.65)	5.10 (3.40, 7.23)	0.0 (-0.2, 0.2)	494.17 (318.00, 714.37)	185.97 (121.80, 265.95)	0.1 (-0.1, 0.3)
Venezuela (Bolivarian Republic of)	1,632.96 (1,245.24, 2,096.99)	5.84 (4.46, 7.50)	-0.4 (-0.8, -0.1)	800.27 (598.38, 1,020.87)	4.13 (3.21, 5.32)	-0.8 (-1.6, 0.0)	47,363.06 (36,493.34, 61,151.94)	170.84 (131.89, 219.72)	-0.7 (-1.4, 0.0)
Viet Nam	4,524.75 (3,430.63, 5,834.20)	4.95 (3.81, 6.34)	0.1 (0.0, 0.2)	39.89 (28.12, 51.22)	3.81 (2.95, 4.83)	-0.2 (-0.3, -0.1)	120,340.11 (91,544.50	128.66 (98.87, 163.62)	-0.5 (-0.5, -0.4)

							, 153,698.21)		
Yemen	1,891.93 (1,242.62, 2,739.81)	8.32 (5.60, 11.91)	0.0 (-0.2, 0.1)	1.93 (1.58, 2.40)	6.57 (4.47, 9.14)	0.1 (0.0, 0.2)	58,648.79 (37,476.25, 86,900.70)	226.14 (148.08, 326.31)	-0.2 (-0.4, -0.1)
Zambia	819.63 (539.41, 1,292.13)	5.08 (3.76, 6.88)	-0.8 (-0.9, -0.7)	2,186.24 (1,704.40, 2,744.26)	3.63 (2.82, 4.56)	-0.5 (-0.6, -0.3)	26,898.14 (17,449.08, 41,759.06)	147.43 (106.74, 205.82)	-1.0 (-1.2, -0.9)
Zimbabwe	383.21 (294.79, 494.91)	3.58 (2.75, 4.78)	-0.3 (-0.7, 0.2)	694.10 (462.46, 972.43)	2.88 (2.19, 3.89)	-0.2 (-0.5, 0.1)	12,625.41 (9,641.29, 16,278.27)	100.62 (77.14, 131.14)	-0.2 (-0.6, 0.3)

Abbreviations: AAPC, average annual percentage change; CI, confidence interval; UI, uncertainty interval.

Supplementary Table 3. The AAPCs (95% CI) of ASIR, ASDR and age-standardized DALY rate due to 4 leukemia subtypes from 1990 to 2019.

Characteristics	AML			CML			ALL			CLL		
	ASIR	ASDR	Age-standardized DALY rate	ASIR	ASDR	Age-standardized DALY rate	ASIR	ASDR	Age-standardized DALY rate	ASIR	ASDR	Age-standardized DALY rate
**Overall**	0.4 (0.3, 0.5)	0.2 (0.2, 0.3)	-0.1 (-0.1, 0.0)	-0.5 (-0.8, -0.2)	-2.2 (-2.3, -2.0)	-2.4 (-2.5, -2.2)	1.6 (1.5, 1.8)	-0.7 (-0.8, -0.6)	-0.9 (-1.0, -0.8)	0.6 (0.3, 0.8)	-0.3 (-0.4, -0.1)	-0.2 (-0.4, -0.1)
**Sex**												
Male	0.5 (0.4, 0.6)	0.4 (0.3, 0.5)	0.0 (0.0, 0.1)	-0.5 (-0.8, -0.2)	-2.0 (-2.2, -1.8)	-2.2 (-2.3, -2.0)	1.7 (1.5, 1.8)	-0.6 (-0.7, -0.5)	-0.8 (-0.9, -0.7)	0.5 (0.3, 0.7)	-0.4 (-0.5, -0.3)	-0.4 (-0.6, -0.3)
Female	0.3 (0.2, 0.3)	0.1 (0.0, 0.1)	-0.2 (-0.3, -0.2)	-0.5 (-0.8, -0.3)	-2.5 (-2.6, -2.4)	-2.6 (-2.8, -2.5)	1.6 (1.4, 1.7)	-0.8 (-0.9, -0.8)	-1.1 (-1.2, -1.0)	0.6 (0.4, 0.8)	-0.2 (-0.3, 0.0)	-0.1 (-0.2, 0.1)
**SDI**												
Low	0.5 (0.4, 0.6)	0.4 (0.4, 0.5)	0.2 (0.2, 0.3)	-2.2 (-2.3, -2.1)	-1.8 (-1.9, -1.7)	-2.5 (-2.6, -2.4)	-0.9 (-1.1, -0.7)	-0.8 (-0.9, -0.7)	-0.9 (-1.1, -0.8)	1.1 (0.9, 1.3)	0.8 (0.7, 1.0)	0.7 (0.6, 0.9)
Low-middle	0.8 (0.7, 0.9)	0.6 (0.5, 0.7)	0.4 (0.3, 0.5)	-1.5 (-1.7, -1.4)	-1.4 (-1.6, -1.3)	-1.9 (-2.1, -1.8)	-0.4 (-0.6, -0.2)	-0.6 (-0.8, -0.4)	-0.9 (-1.2, -0.7)	1.4 (1.2, 1.7)	0.8 (0.3, 1.3)	0.8 (0.6, 1.0)
Middle	0.7 (0.6, 0.9)	0.6 (0.6, 0.7)	0.3 (0.2, 0.4)	-0.9 (-1.0, -0.7)	-1.2 (-1.3, -1.1)	-1.5 (-1.6, -1.4)	1.8 (1.4, 2.1)	-0.1 (-0.2, 0.0)	-0.5 (-0.6, -0.4)	2.6 (2.4, 2.8)	0.9 (0.7, 1.1)	0.9 (0.7, 1.1)
High-middle	0.2 (0.1, 0.3)	0.1 (0.0, 0.2)	-0.5 (-0.6, -0.4)	0.3 (0.0, 0.5)	-2.5 (-2.7, -2.4)	-2.8 (-3.0, -2.6)	2.9 (2.7, 3.1)	-1.0 (-1.2, -0.8)	-1.2 (-1.4, -1.0)	1.6 (1.3, 1.8)	0.1 (-0.1, 0.3)	0.0 (-0.3, 0.2)
High	0.4 (0.3, 0.4)	0.3 (0.3, 0.4)	-0.2 (-0.3, -0.2)	0.3 (0.0, 0.6)	-3.8 (-4.0, -3.6)	-4.0 (-4.2, -3.8)	1.9 (1.7, 2.1)	-1.5 (-1.6, -1.4)	-1.7 (-1.7, -1.6)	0.1 (-0.2, 0.4)	-0.9 (-1.1, -0.8)	-1.1 (-1.2, -0.9)
**Regions**												

Central Asia	0.0 (0.0, 0.1)	-0.1 (-0.2, 0.0)	-0.4 (-0.5, -0.3)	-1.5 (-1.8, -1.1)	-1.4 (-1.8, -1.1)	-2.1 (-2.5, -1.7)	-0.1 (-0.2, -0.1)	-1.1 (-1.4, -0.9)	-1.4 (-1.6, -1.1)	1.3 (0.9, 1.6)	0.5 (0.4, 0.7)	0.0 (-0.2, 0.1)
Central Europe	0.8 (0.6, 0.9)	0.7 (0.6, 0.8)	0.0 (-0.1, 0.1)	-0.4 (-0.6, -0.2)	-2.8 (-3.0, -2.5)	-3.3 (-3.6, -3)	1.9 (1.5, 2.2)	-1.9 (-2.0, -1.8)	-2.1 (-2.2, -2.0)	3.3 (3.0, 3.6)	1.7 (1.6, 1.9)	1.6 (1.4, 1.7)
Eastern Europe	-1.3 (-1.9, -0.7)	-1.2 (-1.9, -0.6)	-1.9 (-2.5, -1.2)	0.5 (-0.8, 1.8)	-0.8 (-1.8, 0.3)	-1.3 (-2.4, -0.1)	0.1 (-0.4, 0.7)	-1.9 (-2.1, -1.6)	-2.4 (-2.6, -2.1)	1.7 (0.8, 2.7)	0.5 (-0.2, 1.3)	0.3 (-0.5, 1.2)
Australasia	0.0 (-0.2, 0.1)	0.0 (-0.1, 0.1)	-0.4 (-0.7, -0.2)	-0.8 (-1.7, 0.1)	-4.2 (-4.7, -3.6)	-4.3 (-4.7, -3.9)	1.0 (0.3, 1.6)	-2.1 (-2.3, -1.9)	-2.3 (-2.5, -2.1)	0.5 (0.1, 1.0)	-0.6 (-1.0, -0.3)	-0.8 (-1.1, -0.4)
High-income Asia Pacific	-0.1 (-0.2, 0.0)	-0.1 (-0.2, 0.1)	-0.8 (-1.0, -0.7)	0.7 (0.4, 0.9)	-4.4 (-4.7, -4.2)	-4.8 (-5.1, -4.6)	2.7 (2.1, 3.3)	-1.8 (-2.0, -1.7)	-1.9 (-2.1, -1.8)	1.0 (0.8, 1.1)	-0.8 (-1.1, -0.5)	-0.7 (-0.9, -0.4)
High-income North America	0.6 (0.5, 0.7)	0.5 (0.4, 0.6)	0.0 (-0.2, 0.2)	-1.3 (-1.6, -1.0)	-3.8 (-4.1, -3.5)	-4.3 (-4.6, -4.0)	0.4 (0.3, 0.5)	-1.1 (-1.2, -0.9)	-1.4 (-1.6, -1.2)	-0.2 (-0.6, 0.1)	-0.8 (-0.9, -0.6)	-1.1 (-1.2, -0.9)
Southern Latin America	0.5 (0.4, 0.6)	0.4 (0.3, 0.5)	0.0 (-0.2, 0.1)	-2.4 (-3.0, -1.9)	-3.0 (-3.4, -2.6)	-3.4 (-3.8, -3.1)	1.0 (0.7, 1.4)	-0.5 (-0.6, -0.3)	-0.8 (-1.0, -0.6)	0.7 (0.3, 1.1)	-0.4 (-0.7, -0.2)	-0.6 (-0.8, -0.4)
Western Europe	0.7 (0.5, 0.8)	0.7 (0.6, 0.8)	0.0 (0.0, 0.1)	1.5 (1.1, 2.0)	-3.8 (-4.1, -3.5)	-4.0 (-4.3, -3.8)	2.4 (2.1, 2.6)	-1.6 (-1.7, -1.5)	-1.6 (-1.8, -1.5)	0.9 (0.6, 1.2)	-0.4 (-0.6, -0.3)	-0.7 (-0.8, -0.5)
Andean Latin America	1.6 (1.5, 1.7)	1.4 (1.3, 1.5)	1.1 (0.9, 1.2)	-0.1 (-0.6, 0.3)	-0.1 (-0.5, 0.3)	-0.5 (-1.0, -0.1)	0.7 (0.4, 0.9)	0.4 (0.1, 0.6)	0.1 (-0.1, 0.4)	2.7 (2.1, 3.4)	1.6 (0.9, 2.2)	1.3 (0.6, 1.9)
Caribbean	0.5 (0.3, 0.7)	0.3 (0.1, 0.4)	0.1 (-0.1, 0.3)	-0.9 (-1.3, -0.6)	-1.4 (-1.6, -1.2)	-1.6 (-1.7, -1.5)	0.4 (0.2, 0.6)	-0.3 (-0.5, 0.0)	-0.3 (-0.4, -0.1)	0.8 (0.7, 1.0)	-0.1 (-0.3, 0.0)	-0.1 (-0.4, 0.2)
Central Latin America	1.1 (0.9, 1.4)	1.0 (0.7, 1.3)	0.7 (0.4, 0.9)	-1.2 (-1.4, -1.0)	-1.4 (-1.6, -1.2)	-1.7 (-1.9, -1.5)	1.3 (1.2, 1.4)	0.5 (0.3, 0.8)	0.3 (0.1, 0.5)	1.4 (1.1, 1.7)	0.2 (-0.1, 0.6)	0.1 (-0.2, 0.5)
Tropical Latin America	0.4 (0.3, 0.4)	0.2 (0.2, 0.3)	-0.2 (-0.3, -0.1)	-2.9 (-3.1, -2.8)	-2.9 (-3.0, -2.7)	-3.6 (-3.7, -3.4)	-0.2 (-0.4, 0.0)	-0.6 (-0.7, -0.4)	-0.8 (-1.0, -0.6)	1.3 (1.1, 1.4)	0.4 (0.3, 0.6)	0.2 (0.0, 0.3)

North Africa and Middle East	0.0 (-0.1, 0.1)	-0.2 (-0.2, -0.1)	-0.5 (-0.6, -0.4)	-1.6 (-1.6, -1.5)	-1.9 (-2.0, -1.8)	-2.2 (-2.3, -2.1)	-0.1 (-0.2, 0.1)	-1.1 (-1.2, -1.0)	-1.4 (-1.5, -1.2)	2.1 (1.8, 2.3)	0.5 (0.4, 0.7)	0.2 (0.0, 0.4)
South Asia	0.7 (0.5, 0.8)	0.4 (0.3, 0.6)	0.2 (0.1, 0.3)	-1.7 (-1.9, -1.5)	-1.6 (-2.0, -1.3)	-2.1 (-2.4, -1.8)	-1.0 (-1.5, -0.6)	-1.2 (-1.6, -0.8)	-1.4 (-1.8, -0.9)	1.1 (0.9, 1.4)	0.5 (0.0, 1.0)	0.5 (0.3, 0.8)
East Asia	1.0 (0.7, 1.2)	0.8 (0.6, 0.9)	0.3 (0.2, 0.5)	1.3 (0.9, 1.6)	-2.3 (-2.6, -2.1)	-2.7 (-3.0, -2.4)	5.1 (4.5, 5.6)	-0.7 (-0.8, -0.6)	0.1 (-0.2, 0.3)	4.9 (4.6, 5.3)	1.8 (1.5, 2.0)	1.7 (1.5, 2.0)
Oceania	0.1 (-0.1, 0.2)	-0.2 (-0.3, 0.0)	-0.2 (-0.4, 0.0)	-1.1 (-1.1, -1.0)	-1.1 (-1.1, -1.0)	-1.1 (-1.3, -0.9)	-0.1 (-0.2, -0.1)	-0.3 (-0.6, 0.1)	-0.2 (-0.5, 0.1)	-0.1 (-0.2, 0.1)	-0.4 (-0.5, -0.2)	-0.2 (-0.4, -0.1)
Southeast Asia	1.0 (1.0, 1.1)	0.8 (0.8, 0.9)	0.7 (0.6, 0.8)	-0.6 (-0.8, -0.4)	-0.6 (-0.8, -0.4)	-1.0 (-1.2, -0.8)	-0.1 (-0.2, -0.1)	-0.4 (-0.5, -0.3)	-0.6 (-0.7, -0.5)	2.1 (1.9, 2.4)	1.2 (1.0, 1.4)	1.1 (1.0, 1.1)
Central Sub-Saharan Africa	0.1 (0.0, 0.2)	0.0 (-0.2, 0.1)	-0.3 (-0.5, -0.1)	-1.2 (-1.3, -1.1)	-1.0 (-1.1, -0.9)	-1.6 (-1.7, -1.4)	-0.7 (-1.0, -0.5)	-0.6 (-0.8, -0.4)	-0.8 (-1.1, -0.5)	2.6 (2.3, 2.9)	2.5 (2.2, 2.7)	2.3 (2.1, 2.6)
Eastern Sub-Saharan Africa	0.6 (0.6, 0.7)	0.5 (0.4, 0.5)	0.3 (0.1, 0.5)	-2.6 (-2.7, -2.5)	-2.3 (-2.4, -2.2)	-3.2 (-3.3, -3.0)	-1.0 (-1.2, -0.9)	-1.0 (-1.1, -0.8)	-1.1 (-1.3, -1.0)	1.3 (1.1, 1.4)	1.1 (0.9, 1.2)	0.9 (0.8, 1.0)
Southern Sub-Saharan Africa	0.6 (0.5, 0.7)	0.3 (0.2, 0.5)	0.3 (0.1, 0.4)	-1.1 (-1.5, -0.7)	-1.1 (-1.6, -0.7)	-1.2 (-1.5, -0.9)	-0.1 (-0.4, 0.1)	-0.2 (-0.4, 0.0)	-0.3 (-0.5, -0.2)	0.7 (0.2, 1.2)	0.3 (-0.3, 0.9)	0.2 (-0.2, 0.7)
Western Sub-Saharan Africa	0.5 (0.4, 0.7)	0.4 (0.2, 0.5)	0.1 (0.0, 0.3)	-0.2 (-0.3, -0.1)	-0.1 (-0.2, 0.0)	-0.4 (-0.5, -0.2)	-0.3 (-0.4, -0.2)	-0.3 (-0.4, -0.1)	-0.3 (-0.4, -0.2)	0.9 (0.7, 1.0)	0.5 (0.4, 0.7)	0.5 (0.4, 0.7)

Abbreviations: AAPC, average annual percentage change; DALY, disability-adjusted life year; CI, confidence interval; AML, acute myeloid leukemia; CML, chronic myeloid leukemia; ALL, acute lymphoblastic leukemia; CLL, chronic lymphocytic leukemia; SDI, socio-demographic index.

Supplementary Table 4. The average annual percentage changes (AAPCs) of ASIR, ASDR and age-standardized DALY rate due to 4 leukemia subtypes from 1990 to 2019 at a national level.

Regions	AML			CML			ALL			CLL		
	ASIR	ASDR	Age-standardized DALY rate	ASIR	ASDR	Age-standardized DALY rate	ASIR	ASDR	Age-standardized DALY rate	ASIR	ASDR	Age-standardized DALY rate
Afghanistan	0.4 (0.3, 0.5)	0.2 (0.1, 0.3)	0.2 (0.0, 0.4)	-1.2 (-1.3, -1.1)	-1.1 (-1.2, -1.0)	-1.5 (-1.7, -1.3)	-0.4 (-0.6, -0.1)	-0.3 (-0.5, 0.0)	-0.5 (-0.8, -0.1)	0.8 (0.7, 0.8)	0.5 (0.4, 0.6)	0.5 (0.4, 0.5)
Albania	0.4 (0.0, 0.8)	0.1 (-0.3, 0.6)	0.3 (-0.1, 0.7)	0.9 (0.5, 1.3)	-1.4 (-1.7, -1.0)	-0.9 (-1.3, -0.6)	3.1 (2.6, 3.6)	0.0 (-0.7, 0.7)	0.3 (-0.5, 1.0)	4.8 (4.3, 5.3)	2.6 (1.9, 3.3)	2.7 (2.1, 3.3)
Algeria	-0.3 (-0.3, -0.2)	-0.4 (-0.5, -0.3)	-0.7 (-0.8, -0.6)	-2.0 (-2.0, -1.9)	-2.2 (-2.2, -2.1)	-2.6 (-2.7, -2.5)	-1.1 (-1.4, -0.9)	-1.8 (-2.0, -1.5)	-2.0 (-2.3, -1.8)	1.3 (1.1, 1.4)	0.0 (-0.2, 0.1)	-0.1 (-0.2, 0.0)
American Samoa	-0.8 (-0.9, -0.6)	-1.0 (-1.2, -0.9)	-0.9 (-1.1, -0.8)	-1.7 (-2.4, -1.0)	-1.8 (-2.5, -1.1)	-1.6 (-2.0, -1.2)	-0.6 (-1.1, 0.0)	-0.7 (-1.3, -0.1)	-0.5 (-1.1, 0.0)	0.2 (-0.1, 0.5)	-0.3 (-0.6, -0.1)	-0.1 (-0.3, 0.1)
Andorra	-0.1 (-0.2, 0.0)	-0.2 (-0.3, -0.2)	-0.4 (-0.5, -0.3)	2.7 (2.3, 3.0)	-2.9 (-3.0, -2.8)	-2.5 (-2.7, -2.4)	2.4 (2.2, 2.6)	-1.3 (-1.3, -1.2)	-1.2 (-1.4, -1.0)	1.3 (1.1, 1.5)	-0.3 (-0.4, -0.2)	-0.1 (-0.2, 0.0)
Angola	0.5 (0.1, 0.9)	0.4 (0.0, 0.7)	0.1 (-0.4, 0.5)	-0.8 (-1.3, -0.4)	-0.6 (-1.0, -0.3)	-1.3 (-1.8, -0.9)	-0.5 (-1.1, 0.1)	-0.4 (-1.0, 0.2)	-0.5 (-0.8, -0.2)	3.3 (3.1, 3.5)	3.1 (2.9, 3.2)	2.8 (2.6, 3.0)
Antigua and Barbuda	1.5 (1.2, 1.8)	1.4 (1.2, 1.5)	1.2 (0.9, 1.6)	-1.2 (-1.5, -0.9)	-1.4 (-1.8, -0.9)	-1.7 (-2.1, -1.3)	0.7 (0.2, 1.2)	0.3 (0.0, 0.7)	0.3 (-0.2, 0.8)	1.4 (0.6, 2.1)	0.8 (-0.1, 1.7)	0.4 (-0.3, 1.0)
Argentina	0.6 (0.4, 0.8)	0.5 (0.3, 0.6)	0.0 (-0.2, 0.2)	-2.9 (-3.4, -2.4)	-3.2 (-3.6, -2.7)	-3.5 (-3.8, -3.1)	0.4 (0.2, 0.7)	-0.4 (-0.6, -0.2)	-0.6 (-0.7, -0.5)	0.4 (0.0, 0.7)	-0.6 (-0.8, -0.4)	-0.7 (-1.0, -0.5)
Armenia	0.8 (0.1, 1.5)	0.7 (0.1, 1.3)	0.2 (-0.5, 1.0)	-1.6 (-2.0, -1.1)	-1.9 (-2.4, -1.5)	-2.3 (-2.9, -1.7)	-1.4 (-1.9, -0.9)	-2.7 (-3.2, -2.1)	-2.7 (-3.1, -2.3)	0.7 (0.1, 1.3)	-0.5 (-1.0, 0.0)	-0.9 (-1.4, -0.4)

Australia	0.0 (-0.2, 0.2)	-0.1 (-0.2, 0.1)	-0.5 (-0.6, -0.3)	-0.8 (-1.7, 0.1)	-4.1 (-4.7, -3.5)	-4.3 (-4.8, -3.7)	1.0 (0.7, 1.3)	-2.1 (-2.3, -2.0)	-2.3 (-2.6, -2.1)	0.4 (-0.1, 0.9)	-0.7 (-1.2, -0.3)	-0.9 (-1.2, -0.5)
Austria	1.5 (1.2, 1.7)	1.4 (1.1, 1.6)	0.7 (0.4, 0.9)	1.6 (1.3, 2.0)	-3.6 (-3.8, -3.3)	-3.7 (-4.0, -3.3)	3.4 (3.0, 3.8)	-1.0 (-1.2, -0.7)	-1.0 (-1.2, -0.7)	0.7 (0.3, 1.2)	-0.5 (-0.7, -0.2)	-0.8 (-1.0, -0.6)
Azerbaijan	0.7 (0.4, 1.1)	0.5 (0.2, 0.8)	0.3 (-0.1, 0.7)	-0.7 (-1.1, -0.4)	-0.6 (-0.9, -0.3)	-1.4 (-1.9, -0.9)	-0.1 (-0.6, 0.5)	-0.3 (-0.8, 0.2)	-0.5 (-1.3, 0.3)	2.0 (1.9, 2.2)	1.3 (1.0, 1.5)	0.7 (0.6, 0.9)
Bahamas	0.6 (0.3, 0.9)	0.3 (-0.1, 0.8)	0.4 (-0.1, 0.9)	-1.1 (-1.8, -0.5)	-1.2 (-1.8, -0.6)	-1.2 (-1.8, -0.6)	-0.1 (-0.7, 0.5)	-0.3 (-0.9, 0.3)	-0.4 (-1.2, 0.3)	0.6 (-0.2, 1.3)	0.0 (-0.6, 0.6)	-0.1 (-0.6, 0.5)
Bahrain	-0.4 (-0.7, 0.0)	-0.6 (-0.9, -0.2)	-0.8 (-1.0, -0.6)	-2.1 (-3.0, -1.2)	-2.7 (-3.8, -1.6)	-3.0 (-3.6, -2.5)	0.4 (-0.4, 1.1)	-1.5 (-2.0, -0.9)	-1.6 (-2.2, -1.0)	0.2 (-0.7, 1.1)	-1.4 (-2.2, -0.5)	-1.8 (-2.7, -0.9)
Bangladesh	0.0 (-0.2, 0.2)	-0.1 (-0.3, 0.1)	-0.4 (-0.6, -0.2)	-3.1 (-3.4, -2.8)	-2.9 (-3.3, -2.6)	-3.6 (-3.9, -3.4)	-1.8 (-2.1, -1.5)	-1.8 (-2.1, -1.4)	-1.9 (-2.3, -1.6)	0.5 (0.2, 0.9)	-0.2 (-0.6, 0.2)	-0.3 (-0.6, 0.1)
Barbados	0.7 (0.2, 1.1)	0.5 (0.2, 0.8)	0.2 (-0.2, 0.5)	-0.2 (-0.7, 0.3)	-0.4 (-0.9, 0.0)	-0.7 (-1.3, 0.0)	0.1 (-0.6, 0.7)	-0.5 (-1.2, 0.1)	-0.8 (-1.4, -0.2)	1.8 (1.2, 2.4)	0.9 (0.8, 1.0)	0.7 (0.6, 0.8)
Belarus	-1.3 (-1.6, -1.0)	-1.3 (-1.7, -1.0)	-2.2 (-2.5, -1.8)	0.2 (-0.6, 1.0)	-1.7 (-2.6, -0.9)	-2.3 (-3.1, -1.5)	-0.3 (-1.0, 0.4)	-3.5 (-4.3, -2.8)	-4.0 (-4.5, -3.5)	1.6 (1.3, 1.9)	-0.2 (-0.4, 0.0)	-0.4 (-0.6, -0.2)
Belgium	1.1 (0.7, 1.5)	1.0 (0.6, 1.4)	0.4 (0.1, 0.8)	2.4 (1.5, 3.4)	-3.2 (-3.9, -2.4)	-3.1 (-3.9, -2.4)	2.5 (2.1, 2.9)	-2.1 (-2.3, -1.9)	-2.2 (-2.3, -2.0)	0.8 (0.2, 1.4)	-0.7 (-1.7, 0.3)	-0.6 (-1.3, 0.1)
Belize	1.5 (1.1, 1.8)	1.2 (0.9, 1.6)	1.1 (0.9, 1.2)	0.1 (-0.2, 0.4)	0.1 (-0.2, 0.4)	0.0 (-0.4, 0.5)	-0.4 (-0.8, 0.1)	-0.4 (-0.9, 0.1)	-0.7 (-1.2, -0.2)	2.2 (1.4, 3.0)	1.7 (1.1, 2.3)	1.7 (1.1, 2.2)
Benin	0.7 (0.4, 1.0)	0.5 (0.3, 0.8)	0.3 (0.0, 0.7)	-0.3 (-0.5, -0.1)	-0.2 (-0.4, 0.0)	-0.3 (-0.5, -0.1)	0.0 (-0.2, 0.2)	0.0 (-0.2, 0.2)	0.1 (-0.2, 0.3)	0.7 (0.6, 0.8)	0.5 (0.4, 0.6)	0.4 (0.2, 0.5)
Bermuda	-0.4 (-0.7, -0.2)	-0.6 (-0.9, -0.4)	-0.7 (-0.9, -0.5)	0.1 (-0.1, 0.3)	-2.4 (-2.7, -2.2)	-2.6 (-2.8, -2.3)	3.1 (2.8, 3.4)	-1.5 (-1.7, -1.2)	-1.2 (-1.5, -1.0)	1.0 (0.4, 1.5)	-0.9 (-1.5, -0.3)	-1.0 (-1.5, -0.4)

Bhutan	1.3 (1.1, 1.4)	1.1 (1.0, 1.2)	0.9 (0.6, 1.1)	-2.1 (-2.3, -1.9)	-1.8 (-2.0, -1.6)	-2.8 (-3.1, -2.5)	-0.5 (-1.1, 0.0)	-0.5 (-1.0, 0.0)	-0.7 (-1.3, 0.0)	1.7 (1.6, 1.8)	0.9 (0.8, 1.0)	0.7 (0.7, 0.8)
Bolivia (Plurinational State of)	1.4 (1.3, 1.5)	1.3 (1.2, 1.3)	0.9 (0.8, 0.9)	-0.4 (-0.5, -0.4)	-0.3 (-0.3, -0.2)	-1.0 (-1.1, -1.0)	-0.1 (-0.3, 0.1)	0.0 (-0.2, 0.2)	-0.4 (-0.6, -0.3)	2.7 (2.5, 2.9)	2.1 (2.0, 2.1)	1.6 (1.6, 1.7)
Bosnia and Herzegovina	1.1 (0.9, 1.4)	1.0 (0.7, 1.2)	0.7 (0.3, 1.0)	1.0 (0.6, 1.3)	0.2 (-0.3, 0.7)	-0.3 (-0.7, 0.2)	1.6 (0.9, 2.4)	-0.4 (-1.3, 0.4)	-0.5 (-1.7, 0.7)	4.8 (4.2, 5.3)	3.2 (2.7, 3.7)	3.0 (2.6, 3.3)
Botswana	1.6 (1.3, 1.9)	1.3 (1.1, 1.6)	1.6 (1.4, 1.7)	0.0 (-0.2, 0.2)	-0.1 (-0.3, 0.1)	0.1 (-0.1, 0.4)	1.2 (0.9, 1.6)	1.1 (0.7, 1.4)	1.4 (1.1, 1.6)	2.2 (1.9, 2.4)	1.4 (1.3, 1.6)	1.4 (1.2, 1.7)
Brazil	0.3 (0.3, 0.4)	0.2 (0.1, 0.3)	-0.2 (-0.3, -0.1)	-3.0 (-3.2, -2.8)	-2.9 (-3.1, -2.8)	-3.7 (-3.8, -3.5)	-0.3 (-0.4, -0.1)	-0.6 (-0.8, -0.5)	-0.9 (-1.0, -0.7)	1.2 (1.0, 1.4)	0.4 (0.2, 0.5)	0.1 (0.0, 0.2)
Brunei Darussalam	0.1 (-0.1, 0.4)	-0.1 (-0.4, 0.2)	-0.1 (-0.4, 0.1)	-1.2 (-1.9, -0.6)	-1.4 (-2.1, -0.7)	-1.9 (-2.2, -1.5)	2.0 (1.6, 2.4)	1.2 (0.8, 1.6)	1.0 (0.5, 1.4)	1.4 (1.0, 1.9)	0.4 (0.2, 0.7)	0.3 (0.1, 0.5)
Bulgaria	1.1 (0.9, 1.3)	1.0 (0.7, 1.2)	0.7 (0.5, 1.0)	-1.6 (-2.2, -1.0)	-2.1 (-2.5, -1.6)	-2.6 (-3.2, -2.0)	0.6 (0.2, 1.0)	-0.8 (-1.1, -0.5)	-1.1 (-1.6, -0.7)	2.3 (1.9, 2.6)	1.2 (0.9, 1.5)	1.1 (0.9, 1.4)
Burkina Faso	1.1 (1.0, 1.3)	1.0 (0.7, 1.2)	0.9 (0.7, 1.1)	0.1 (0.5, 3.3)	0.4 (0.2, 0.6)	0.4 (0.1, 0.6)	0.7 (0.4, 0.9)	0.6 (0.4, 0.9)	0.8 (0.6, 1.1)	1.0 (0.6, 1.3)	0.7 (0.4, 1.0)	0.7 (0.4, 1.0)
Burundi	1.0 (0.8, 1.3)	0.9 (0.6, 1.2)	0.6 (0.4, 0.9)	-2.0 (-2.5, -1.5)	-1.8 (-2.2, -1.4)	-2.3 (-3.0, -1.6)	0.1 (-0.2, 0.4)	0.2 (-0.2, 0.5)	0.0 (-0.3, 0.3)	0.7 (0.5, 0.9)	0.6 (0.5, 0.8)	0.5 (0.3, 0.7)
Cabo Verde	1.2 (1.0, 1.5)	1.2 (0.9, 1.5)	0.6 (0.4, 0.8)	1.0 (0.5, 1.4)	1.1 (0.4, 1.9)	0.1 (-0.3, 0.4)	0.1 (-0.3, 0.5)	0.1 (-0.4, 0.5)	-0.6 (-0.9, -0.3)	3.5 (2.3, 4.8)	2.9 (2.3, 3.6)	2.5 (1.9, 3.2)
Cambodia	1.0 (0.9, 1.1)	0.8 (0.7, 0.9)	0.6 (0.4, 0.7)	-0.6 (-0.7, -0.5)	-0.5 (-0.7, -0.3)	-1.1 (-1.2, -0.9)	-0.6 (-0.7, -0.4)	-0.5 (-0.6, -0.3)	-0.9 (-1.2, -0.6)	1.6 (1.5, 1.8)	1.1 (1.0, 1.1)	0.8 (0.8, 0.9)

Cameroon	0.7 (0.3, 1.0)	0.5 (0.2, 0.7)	0.4 (-0.1, 0.9)	-0.3 (-0.4, -0.3)	-0.3 (-0.4, -0.3)	-0.3 (-0.4, -0.2)	1.1 (0.6, 1.6)	1.1 (0.5, 1.6)	1.3 (0.7, 1.9)	0.7 (0.5, 0.9)	0.4 (0.2, 0.6)	0.3 (0.1, 0.5)
Canada	0.9 (0.8, 1.0)	0.7 (0.6, 0.9)	0.3 (0.2, 0.4)	1.2 (0.7, 1.8)	-3.8 (-4.1, -3.4)	-3.8 (-4.1, -3.4)	1.8 (1.3, 2.2)	-1.6 (-1.8, -1.3)	-1.6 (-2.0, -1.2)	0.6 (-0.1, 1.2)	-0.7 (-1.3, 0.0)	-0.8 (-1.4, -0.3)
Central African Republic	0.0 (-0.1, 0.1)	-0.2 (-0.3, -0.1)	-0.3 (-0.4, -0.2)	-0.6 (-0.7, -0.4)	-0.6 (-0.7, -0.4)	-0.6 (-0.8, -0.4)	-0.2 (-0.4, -0.1)	-0.3 (-0.4, -0.1)	-0.2 (-0.4, -0.1)	2.2 (1.8, 2.5)	2.2 (1.9, 2.5)	2.0 (1.7, 2.3)
Chad	1.1 (0.8, 1.3)	0.9 (0.7, 1.1)	0.8 (0.4, 1.1)	0.6 (0.4, 0.7)	0.7 (0.6, 0.8)	0.4 (0.3, 0.6)	0.6 (0.2, 0.9)	0.6 (0.2, 1.0)	0.6 (0.2, 1.0)	0.8 (0.7, 0.9)	0.7 (0.6, 0.8)	0.6 (0.5, 0.7)
Chile	0.7 (0.4, 1.0)	0.7 (0.4, 0.9)	0.1 (-0.1, 0.3)	-1.4 (-2.2, -0.6)	-2.6 (-3.1, -2.1)	-3.2 (-3.9, -2.5)	2.2 (1.8, 2.7)	-0.5 (-1.0, -0.1)	-0.8 (-1.3, -0.4)	1.8 (1.7, 2.0)	0.2 (0.1, 0.4)	-0.1 (-0.2, 0.1)
China	0.8 (0.7, 1.0)	0.7 (0.5, 0.8)	0.3 (0.2, 0.4)	1.2 (0.8, 1.6)	-2.4 (-2.8, -2.1)	-2.7 (-3.0, -2.4)	5.1 (4.6, 5.6)	0.4 (0.2, 0.6)	0.2 (-0.1, 0.4)	5.1 (4.7, 5.4)	1.8 (1.5, 2.0)	1.8 (1.5, 2.0)
Colombia	1.4 (1.2, 1.7)	1.3 (1.0, 1.5)	1.2 (0.9, 1.4)	-0.9 (-1.7, -0.2)	-1.6 (-2.4, -0.9)	-2.1 (-2.4, -1.7)	1.6 (1.3, 1.9)	0.4 (0.0, 0.7)	0.4 (0.0, 0.7)	0.5 (-0.4, 1.4)	-0.9 (-1.8, 0.0)	-1.1 (-2.1, -0.1)
Comoros	0.8 (-0.5, 2.2)	0.7 (-0.3, 1.7)	0.5 (-1.0, 2.1)	-0.5 (-1.8, 0.8)	-0.5 (-1.9, 1.0)	-0.7 (-2.2, 0.9)	0.5 (-1.1, 2.1)	0.5 (-1.0, 2.1)	0.5 (-1.1, 2.2)	1.9 (1.8, 2.0)	1.6 (1.4, 1.7)	1.5 (1.3, 1.7)
Congo	0.2 (0.0, 0.4)	0.1 (-0.1, 0.2)	-0.1 (-0.4, 0.1)	-1.4 (-1.6, -1.2)	-1.3 (-1.5, -1.1)	-1.6 (-1.9, -1.3)	-0.7 (-1.2, -0.3)	-0.7 (-1.1, -0.3)	-0.8 (-1.2, -0.3)	2.2 (1.9, 2.4)	1.9 (1.7, 2.1)	1.8 (1.5, 2.0)
Cook Islands	-0.4 (-0.4, -0.3)	-0.5 (-0.6, -0.5)	-0.7 (-0.8, -0.7)	-0.9 (-1.1, -0.8)	-1.4 (-1.6, -1.3)	-1.5 (-1.6, -1.5)	-0.3 (-0.4, -0.2)	-1.3 (-1.4, -1.2)	-1.7 (-1.8, -1.6)	-0.1 (-0.2, 0.1)	-1.3 (-1.4, -1.2)	-1.2 (-1.3, -1.1)
Costa Rica	0.6 (0.0, 1.2)	0.4 (0.0, 0.9)	0.3 (0.1, 0.6)	-1.5 (-3.0, 0.0)	-2.2 (-3.2, -1.3)	-2.2 (-3.3, -1.1)	1.2 (0.2, 2.3)	-0.3 (-1.3, 0.6)	-0.5 (-1.4, 0.4)	0.9 (-0.6, 2.5)	0.0 (-1.5, 1.6)	-0.1 (-1.7, 1.5)
Croatia	2.7 (2.1, 3.3)	2.8 (2.2, 3.4)	1.5 (1.0, 2.0)	2.6 (2.1, 3.2)	-1.4 (-1.9, -0.9)	-2.0 (-2.3, -1.7)	-0.2 (-0.5, 0.0)	-0.5 (-1.0, 0.0)	-0.2 (-0.4, 0.0)	0.7 (-0.2, 1.6)	-0.7 (-1.4, 0.0)	-0.9 (-1.8, -0.1)

Cuba	0.0 (-0.3, 0.2)	-0.1 (-0.3, 0.1)	-0.6 (-1.0, -0.1)	-0.6 (-1.2, -0.1)	-1.6 (-1.7, -1.5)	-1.9 (-2.2, -1.6)	4.0 (3.5, 4.6)	-1.3 (-1.7, -0.9)	-0.8 (-1.4, -0.2)	0.8 (0.7, 0.9)	-0.3 (-0.5, -0.2)	-0.4 (-0.7, -0.2)
Cyprus	1.0 (0.8, 1.2)	0.9 (0.6, 1.1)	0.4 (0.2, 0.6)	4.1 (3.2, 5.0)	-3.2 (-4.3, -2.0)	-2.5 (-3.1, -1.9)	0.2 (-0.2, 0.7)	-1.2 (-1.4, -0.9)	-1.6 (-2.0, -1.2)	3.1 (2.5, 3.7)	0.7 (0.1, 1.2)	0.7 (0.2, 1.2)
Czechia	-0.3 (-0.4, -0.1)	-0.4 (-0.5, -0.2)	-0.9 (-1.1, -0.7)	1.1 (-0.1, 2.2)	-3.1 (-3.8, -2.4)	-3.2 (-3.6, -2.7)	5.9 (4.8, 7.1)	-4.6 (-5.0, -4.2)	-0.8 (-1.1, -0.4)	2.7 (1.8, 3.6)	1.2 (0.3, 2.0)	0.8 (0.2, 1.4)
Côte d'Ivoire	0.5 (0.3, 0.6)	0.3 (0.1, 0.5)	0.2 (0.0, 0.4)	-0.3 (-0.4, -0.1)	-0.2 (-0.4, -0.1)	-0.3 (-0.5, -0.2)	0.6 (-0.4, 1.6)	-0.2 (-0.5, 0.0)	-4.3 (-4.7, -3.9)	0.5 (0.3, 0.6)	0.3 (0.1, 0.5)	0.2 (0.1, 0.3)
Democratic People's Republic of Korea	0.0 (-0.1, 0.0)	-0.2 (-0.3, -0.1)	-0.4 (-0.5, -0.3)	-1.9 (-2.0, -1.8)	-1.9 (-2.0, -1.7)	-2.1 (-2.1, -2.0)	-0.2 (-0.3, -0.1)	-0.1 (-0.2, 0.0)	-0.5 (-0.7, -0.3)	0.7 (0.5, 0.8)	0.4 (0.2, 0.5)	0.4 (0.3, 0.6)
Democratic Republic of the Congo	-0.1 (-0.3, 0.1)	-0.3 (-0.5, 0.0)	-0.5 (-0.8, -0.3)	-1.4 (-1.7, -1.2)	-1.3 (-1.5, -1.0)	-1.8 (-2.0, -1.6)	-0.8 (-1.2, -0.5)	-0.7 (-1.1, -0.3)	-0.9 (-1.3, -0.5)	2.5 (2.4, 2.7)	2.4 (2.1, 2.7)	2.3 (1.9, 2.6)
Denmark	-1.5 (-1.9, -1.1)	-1.3 (-1.6, -1.0)	-2.0 (-2.5, -1.5)	-0.5 (-1.1, 0.0)	-5.1 (-5.7, -4.6)	-5.3 (-5.8, -4.8)	1.8 (1.0, 2.6)	-2.6 (-3.2, -2.1)	-2.8 (-3.3, -2.4)	0.7 (0.5, 0.9)	-0.7 (-0.9, -0.4)	-1.0 (-1.2, -0.9)
Djibouti	1.5 (1.1, 2.0)	1.4 (1.0, 1.7)	1.2 (0.9, 1.6)	0.1 (-0.2, 0.4)	0.2 (0.0, 0.5)	-0.1 (-0.4, 0.3)	1.2 (0.9, 1.6)	1.2 (0.8, 1.5)	1.3 (0.9, 1.6)	1.9 (1.8, 2.1)	1.6 (1.5, 1.7)	1.7 (1.5, 1.8)
Dominica	1.0 (0.7, 1.3)	0.6 (0.5, 0.8)	0.7 (0.5, 0.9)	0.1 (0.0, 0.3)	0.2 (0.0, 0.4)	0.1 (-0.1, 0.2)	0.7 (0.3, 1.0)	0.6 (0.3, 0.9)	0.6 (0.3, 1.0)	1.1 (0.7, 1.4)	0.9 (0.7, 1.2)	0.8 (0.4, 1.2)
Dominican Republic	2.6 (2.2, 3.1)	2.5 (2.1, 2.8)	2.1 (1.7, 2.6)	1.7 (0.9, 2.5)	1.7 (1.0, 2.5)	1.4 (0.6, 2.2)	0.7 (0.3, 1.2)	0.8 (0.3, 1.2)	0.3 (-0.2, 0.8)	3.6 (3.0, 4.3)	2.9 (2.3, 3.4)	2.8 (2.3, 3.3)

Ecuador	2.4 (2.1, 2.6)	2.2 (2.0, 2.4)	1.8 (1.5, 2.2)	0.6 (0.2, 0.9)	0.6 (0.3, 0.9)	0.2 (-0.1, 0.6)	1.4 (1.1, 1.8)	1.2 (0.8, 1.5)	1.0 (0.6, 1.4)	3.5 (2.8, 4.2)	2.3 (1.5, 3.1)	2.2 (1.6, 2.8)
Egypt	0.6 (0.3, 0.8)	0.4 (0.2, 0.6)	0.1 (-0.1, 0.4)	-0.8 (-0.9, -0.8)	-0.8 (-0.9, -0.6)	-1.3 (-1.4, -1.2)	-0.6 (-1.1, -0.1)	-0.8 (-1.0, -0.6)	-1.2 (-1.5, -0.8)	2.9 (1.9, 4.0)	1.7 (1.1, 2.4)	1.5 (1.4, 1.7)
El Salvador	3.3 (2.8, 3.8)	3.2 (2.9, 3.6)	2.7 (2.4, 3.0)	1.7 (1.1, 2.3)	1.8 (1.1, 2.5)	1.0 (0.4, 1.7)	2.7 (2.3, 3.1)	2.4 (2.0, 2.8)	1.9 (1.5, 2.2)	4.7 (4.0, 5.5)	3.5 (2.8, 4.2)	3.3 (2.6, 4.1)
Equatorial Guinea	2.0 (1.7, 2.2)	1.9 (1.6, 2.1)	1.4 (1.0, 1.9)	-1.1 (-1.4, -0.9)	-0.9 (-1.0, -0.8)	-1.7 (-2.0, -1.5)	-1.3 (-1.9, -0.7)	-1.2 (-1.7, -0.7)	-1.5 (-2.1, -0.9)	4.5 (4.0, 5.0)	3.9 (3.6, 4.3)	3.5 (3.1, 3.9)
Eritrea	2.0 (1.7, 2.4)	1.8 (1.5, 2.1)	1.8 (1.5, 2.0)	-0.4 (-0.6, -0.1)	-0.2 (-0.4, 0.0)	-0.6 (-0.8, -0.3)	1.1 (0.8, 1.3)	1.0 (0.8, 1.3)	1.1 (0.8, 1.3)	2.5 (2.3, 2.7)	2.3 (2.1, 2.4)	2.2 (2.0, 2.4)
Estonia	0.5 (-0.1, 1.1)	0.5 (-0.1, 1.1)	-0.3 (-0.9, 0.3)	1.6 (1.0, 2.1)	-1.3 (-2.3, -0.4)	-1.9 (-2.5, -1.2)	3.3 (2.8, 3.8)	-1.4 (-1.8, -1.1)	-1.7 (-2.3, -1.2)	4.9 (3.3, 6.5)	3.0 (1.4, 4.6)	2.5 (0.9, 4.1)
Eswatini	1.4 (1.2, 1.5)	1.1 (1.0, 1.2)	1.3 (1.2, 1.4)	0.4 (0.1, 0.6)	0.4 (0.1, 0.6)	0.4 (0.1, 0.6)	1.1 (0.9, 1.4)	1.1 (0.8, 1.3)	1.2 (1.0, 1.3)	1.2 (0.9, 1.6)	1.1 (0.8, 1.4)	1.0 (0.6, 1.4)
Ethiopia	0.3 (0.2, 0.3)	0.2 (0.1, 0.2)	-0.2 (-0.3, -0.1)	-3.1 (-3.2, -2.9)	-2.8 (-2.9, -2.6)	-3.8 (-4.0, -3.5)	-1.6 (-1.9, -1.3)	-1.6 (-1.9, -1.3)	-1.9 (-2.2, -1.5)	0.6 (0.5, 0.8)	0.3 (0.2, 0.5)	0.2 (0.1, 0.3)
Fiji	0.4 (0.0, 0.7)	0.2 (-0.2, 0.5)	0.2 (-0.1, 0.5)	-1.8 (-2.4, -1.2)	-1.8 (-2.4, -1.2)	-2.0 (-2.5, -1.5)	-1.0 (-1.2, -0.7)	-1.0 (-1.1, -0.8)	-0.9 (-1.2, -0.7)	0.0 (-0.1, 0.2)	-0.2 (-0.4, -0.1)	-0.3 (-0.4, -0.2)
Finland	-0.8 (-0.9, -0.6)	-0.6 (-0.8, -0.3)	-1.0 (-1.1, -1.0)	-0.8 (-2.0, 0.4)	-5.0 (-5.6, -4.5)	-5.0 (-5.6, -4.5)	3.3 (3.0, 3.5)	-1.6 (-2.0, -1.1)	-1.5 (-2.2, -0.9)	0.5 (0.0, 0.9)	-1.1 (-1.4, -0.7)	-1.2 (-1.6, -0.9)
France	1.0 (0.8, 1.1)	0.9 (0.6, 1.1)	0.1 (0.0, 0.2)	2.8 (2.2, 3.3)	-3.2 (-3.5, -2.9)	-3.6 (-3.9, -3.3)	3.8 (3.6, 4.0)	-1.5 (-1.6, -1.3)	-1.3 (-1.5, -1.2)	0.8 (0.5, 1.1)	-0.7 (-0.9, -0.5)	-0.9 (-1.1, -0.8)
Gabon	0.3 (0.0, 0.5)	0.2 (-0.1, 0.4)	-0.1 (-0.4, 0.3)	-1.1 (-1.2, -0.9)	-1.0 (-1.1, -0.8)	-1.3 (-1.5, -1.1)	-0.6 (-1.1, -0.2)	-0.6 (-1.0, -0.2)	-0.8 (-1.3, -0.3)	2.5 (2.2, 2.9)	2.2 (1.9, 2.6)	2.0 (1.7, 2.4)

Gambia	0.6 (-0.5, 1.7)	0.5 (-0.5, 1.5)	0.2 (-0.9, 1.3)	0.3 (-0.3, 0.9)	0.3 (-0.3, 0.9)	0.2 (-0.5, 0.9)	0.1 (-0.9, 1.1)	0.2 (-0.8, 1.2)	0.0 (-0.9, 1.0)	1.3 (0.5, 2.1)	1.1 (0.3, 1.8)	1.1 (0.2, 2.0)
Georgia	0.3 (-0.4, 1.0)	0.3 (-0.4, 1.0)	-0.4 (-1.2, 0.4)	-0.2 (-1.3, 0.8)	-0.1 (-1.1, 0.8)	-0.7 (-1.8, 0.3)	-1.4 (-2.2, -0.6)	-1.6 (-2.3, -0.9)	-2.0 (-2.7, -1.3)	3.0 (2.2, 3.9)	3.0 (2.3, 3.7)	2.5 (1.9, 3.1)
Germany	0.8 (0.6, 1.0)	1.0 (0.9, 1.2)	0.2 (-0.1, 0.4)	1.6 (1.1, 2.2)	-3.7 (-4.1, -3.3)	-3.9 (-4.2, -3.7)	2.6 (2.3, 3.0)	-1.4 (-1.6, -1.2)	-1.5 (-1.6, -1.3)	1.3 (0.9, 1.6)	0.0 (-0.2, 0.2)	-0.3 (-0.5, -0.1)
Ghana	0.0 (-0.2, 0.2)	-0.1 (-0.3, 0.1)	-0.4 (-0.6, -0.1)	-0.9 (-1.0, -0.8)	-0.8 (-0.9, -0.7)	-1.2 (-1.3, -1.0)	-2.7 (-3.0, -2.5)	-2.5 (-2.8, -2.3)	-3.0 (-3.3, -2.7)	0.5 (0.3, 0.7)	0.2 (0.1, 0.3)	0.1 (-0.1, 0.3)
Greece	1.7 (1.3, 2.1)	1.6 (1.2, 1.9)	1.1 (0.8, 1.4)	2.5 (1.6, 3.4)	-1.5 (-1.9, -1.2)	-1.5 (-2.0, -1.1)	2.4 (2.1, 2.7)	-0.7 (-1.1, -0.3)	-0.7 (-0.9, -0.5)	1.5 (1.0, 2.0)	0.3 (-0.2, 0.8)	0.3 (-0.2, 0.8)
Greenland	-0.3 (-0.5, -0.1)	-0.5 (-0.6, -0.3)	-0.7 (-0.9, -0.5)	-2.3 (-2.7, -2.0)	-2.4 (-2.8, -2.0)	-2.7 (-2.9, -2.4)	-1.6 (-2.3, -0.9)	-2.1 (-2.4, -1.7)	-2.5 (-3.2, -1.7)	-0.4 (-0.8, -0.1)	-1.3 (-1.7, -0.9)	-1.3 (-1.6, -1.0)
Grenada	0.9 (0.7, 1.2)	0.7 (0.5, 1.0)	0.5 (0.2, 0.8)	-2.8 (-3.2, -2.3)	-2.8 (-3.2, -2.3)	-2.8 (-3.3, -2.4)	-1.5 (-1.8, -1.1)	-1.6 (-1.9, -1.2)	-1.9 (-2.2, -1.5)	-1.4 (-2.1, -0.7)	-2.2 (-2.9, -1.4)	-1.9 (-2.6, -1.3)
Guam	-1.9 (-2.9, -0.9)	-2.3 (-3.3, -1.2)	-1.8 (-2.6, -1.0)	-1.6 (-2.8, -0.5)	-2.0 (-3.1, -0.8)	-1.0 (-2.1, 0.1)	-1.0 (-1.8, -0.1)	-1.3 (-1.9, -0.8)	-1.3 (-1.7, -0.9)	-2.3 (-3.3, -1.3)	-3.4 (-4.2, -2.6)	-2.0 (-2.8, -1.2)
Guatemala	5.4 (4.8, 5.9)	4.9 (4.4, 5.5)	5.1 (4.6, 5.6)	0.7 (0.2, 1.2)	0.8 (0.5, 1.2)	0.6 (0.2, 1.0)	5.6 (5.2, 6.1)	5.6 (5.2, 6.0)	5.5 (5.1, 5.8)	2.9 (2.2, 3.7)	2.0 (1.7, 2.4)	2.0 (1.7, 2.3)
Guinea	0.4 (0.0, 0.7)	0.2 (0.0, 0.5)	0.0 (-0.4, 0.4)	-0.1 (-0.2, 0.0)	-0.1 (-0.1, 0.0)	-0.2 (-0.3, -0.1)	0.1 (-0.3, 0.5)	0.1 (-0.2, 0.5)	0.1 (-0.4, 0.6)	0.5 (0.4, 0.6)	0.3 (0.2, 0.4)	0.3 (0.2, 0.4)
Guinea-Bissau	0.2 (0.0, 0.3)	0.0 (-0.2, 0.2)	-0.4 (-0.6, -0.2)	-0.8 (-0.9, -0.7)	-0.8 (-0.8, -0.7)	-0.9 (-1.0, -0.8)	-0.7 (-1.3, -0.1)	-0.6 (-1.2, -0.1)	-0.6 (-0.9, -0.4)	0.5 (0.4, 0.5)	0.3 (0.2, 0.4)	0.2 (0.1, 0.3)
Guyana	0.8 (0.5, 1.0)	0.5 (0.2, 0.7)	0.6 (0.2, 1.0)	-1.4 (-1.7, -1.2)	-1.4 (-1.7, -1.2)	-1.4 (-1.7, -1.1)	0.8 (-0.2, 1.8)	0.8 (-0.1, 1.8)	0.6 (-0.5, 1.7)	4.2 (3.1, 5.5)	3.6 (3.1, 4.2)	3.9 (3.3, 4.6)

Haiti	0.4 (0.1, 0.7)	0.2 (0.0, 0.4)	0.0 (-0.3, 0.3)	-1.8 (-1.9, -1.7)	-1.6 (-1.7, -1.5)	-2.1 (-2.2, -2.0)	-0.3 (-0.5, 0.0)	-0.2 (-0.4, 0.0)	-0.2 (-0.5, 0.1)	0.5 (0.3, 0.6)	0.3 (0.3, 0.4)	0.2 (0.1, 0.3)
Honduras	0.7 (0.7, 0.8)	0.7 (0.5, 0.9)	0.0 (-0.1, 0.0)	0.1 (-0.5, 0.6)	0.3 (-0.2, 0.9)	-0.6 (-0.9, -0.4)	-0.6 (-0.7, -0.5)	-0.3 (-0.4, -0.3)	-1.3 (-1.5, -1.1)	3.3 (2.7, 3.8)	2.8 (2.3, 3.3)	2.4 (1.9, 2.9)
Hungary	0.6 (0.3, 1.0)	0.5 (0.2, 0.8)	0.0 (-0.3, 0.4)	-1.9 (-2.5, -1.3)	-4.4 (-5.2, -3.6)	-4.8 (-5.5, -4.1)	1.8 (1.3, 2.3)	-2.2 (-2.6, -1.7)	-2.3 (-2.8, -1.8)	1.0 (0.7, 1.2)	-0.6 (-1.1, 0.0)	-0.7 (-1.1, -0.4)
Iceland	0.3 (0.0, 0.5)	0.0 (-0.2, 0.2)	-0.4 (-0.6, -0.2)	2.2 (1.7, 2.7)	-2.6 (-2.9, -2.3)	-2.5 (-2.9, -2.1)	1.3 (0.7, 1.9)	-1.3 (-1.8, -0.7)	-1.5 (-2.1, -0.9)	-1.5 (-2.1, -1.0)	-2.8 (-3.1, -2.5)	-2.7 (-3.1, -2.2)
India	0.6 (0.4, 0.8)	0.4 (0.2, 0.5)	0.1 (-0.1, 0.3)	-1.7 (-2.0, -1.3)	-1.6 (-2.0, -1.3)	-2.1 (-2.5, -1.7)	-1.5 (-2.2, -0.8)	-1.7 (-2.4, -0.9)	-1.9 (-2.8, -1.1)	1.2 (0.8, 1.5)	0.5 (-0.3, 1.3)	0.6 (0.2, 0.9)
Indonesia	1.1 (1.0, 1.1)	0.9 (0.9, 1.0)	0.6 (0.5, 0.6)	-0.5 (-0.5, -0.4)	-0.3 (-0.3, -0.2)	-1.1 (-1.1, -1.0)	-0.5 (-0.6, -0.4)	-0.3 (-0.4, -0.2)	-0.9 (-1.0, -0.7)	2.6 (2.5, 2.8)	2.2 (2.1, 2.4)	1.9 (1.7, 2.0)
Iran (Islamic Republic of)	-0.2 (-0.4, -0.1)	-0.4 (-0.5, -0.3)	-0.7 (-0.9, -0.5)	-1.6 (-1.7, -1.5)	-2.0 (-2.1, -1.9)	-2.4 (-2.6, -2.3)	0.3 (-0.2, 0.7)	-1.3 (-1.5, -1.0)	-1.6 (-1.7, -1.4)	1.7 (1.3, 2.0)	-0.1 (-0.3, 0.0)	-0.2 (-0.4, -0.1)
Iraq	0.5 (0.4, 0.7)	0.4 (0.2, 0.6)	0.2 (0.1, 0.4)	-1.8 (-2.0, -1.6)	-1.8 (-1.9, -1.6)	-2.3 (-2.4, -2.1)	-0.5 (-0.7, -0.2)	-0.8 (-1.0, -0.5)	-1.3 (-1.6, -1.1)	2.1 (1.6, 2.5)	1.0 (0.6, 1.4)	0.8 (0.4, 1.1)
Ireland	0.5 (0.3, 0.7)	0.5 (0.2, 0.8)	0.0 (-0.3, 0.4)	2.3 (1.7, 2.9)	-5.2 (-5.8, -4.6)	-4.7 (-5.5, -4.0)	3.1 (2.8, 3.4)	-2.5 (-2.9, -2.2)	-2.1 (-2.6, -1.6)	1.3 (0.5, 2.0)	-0.6 (-1.2, -0.1)	-0.7 (-1.3, -0.1)
Israel	0.3 (0.2, 0.5)	0.2 (0.1, 0.3)	-0.3 (-0.4, -0.2)	0.9 (0.6, 1.2)	-3.6 (-3.9, -3.4)	-3.8 (-4.2, -3.4)	3.6 (3.3, 4.0)	-1.8 (-2.1, -1.6)	-1.7 (-2.0, -1.4)	0.9 (0.7, 1.1)	-0.7 (-1.0, -0.4)	-0.8 (-1.1, -0.6)
Italy	1.2 (1.1, 1.4)	1.2 (1.2, 1.3)	0.4 (0.3, 0.5)	1.5 (0.9, 2.2)	-3.9 (-4.2, -3.7)	-4.3 (-4.5, -4.0)	2.2 (2.0, 2.5)	-1.2 (-1.5, -0.8)	-1.2 (-1.6, -0.9)	0.8 (0.7, 0.9)	-0.4 (-0.6, -0.2)	-0.7 (-0.8, -0.5)
Jamaica	3.3 (1.6, 5.0)	3.1 (1.5, 4.8)	2.6 (1.4, 3.8)	2.7 (1.0, 4.5)	2.9 (1.4, 4.4)	3.2 (1.5, 4.8)	-0.1 (-1.0, 0.8)	-0.2 (-1.0, 0.6)	-0.6 (-1.5, 0.3)	6.0 (5.2, 6.7)	5.2 (4.5, 5.9)	5.3 (4.5, 6.0)

Japan	-0.1 (-0.2, 0.0)	-0.1 (-0.2, 0.0)	-0.8 (-1.0, -0.6)	0.3 (-0.4, 1.0)	-4.9 (-5.3, -4.5)	-5.4 (-5.8, -4.9)	2.3 (1.9, 2.7)	-1.8 (-2.1, -1.6)	-1.9 (-2.2, -1.5)	0.6 (0.4, 0.8)	-1.0 (-1.2, -0.9)	-1.0 (-1.1, -0.8)
Jordan	1.0 (0.8, 1.3)	0.9 (0.7, 1.1)	0.6 (0.5, 0.8)	-1.3 (-1.7, -1.0)	-1.8 (-2.1, -1.4)	-2.1 (-2.4, -1.8)	1.7 (1.5, 1.8)	0.2 (0.1, 0.3)	0.2 (0.0, 0.4)	2.2 (1.8, 2.7)	0.6 (0.1, 1.1)	0.3 (0.0, 0.5)
Kazakhstan	-0.6 (-0.9, -0.3)	-0.7 (-0.8, -0.5)	-1.2 (-1.4, -0.9)	-2.0 (-2.4, -1.5)	-2.1 (-2.6, -1.7)	-2.7 (-3.3, -2.2)	-0.8 (-1.1, -0.5)	-1.6 (-1.9, -1.4)	-2.1 (-2.5, -1.7)	1.2 (1.0, 1.5)	0.1 (-0.4, 0.6)	-0.3 (-0.8, 0.1)
Kenya	0.8 (0.6, 0.9)	0.8 (0.7, 0.9)	0.6 (0.4, 0.8)	-1.1 (-1.3, -0.9)	-0.6 (-0.9, -0.4)	-1.5 (-1.8, -1.2)	0.1 (-0.2, 0.4)	0.3 (0.0, 0.6)	0.1 (-0.2, 0.4)	1.1 (0.9, 1.3)	1.2 (1.0, 1.3)	1.1 (0.9, 1.2)
Kiribati	0.2 (0.1, 0.3)	0.0 (-0.1, 0.0)	0.0 (-0.2, 0.1)	-0.2 (-0.3, -0.2)	-0.2 (-0.3, -0.1)	-0.3 (-0.4, -0.2)	-0.4 (-0.6, -0.1)	-0.4 (-0.6, -0.1)	-0.3 (-0.5, 0.0)	0.2 (0.0, 0.5)	0.1 (-0.1, 0.3)	0.0 (-0.2, 0.2)
Kuwait	0.9 (-0.1, 1.9)	0.9 (-0.1, 1.9)	0.2 (-0.5, 0.9)	1.5 (-0.1, 3.2)	-0.7 (-2.0, 0.6)	-1.2 (-2.3, 0.0)	2.9 (1.4, 4.4)	-1.2 (-2.3, -0.1)	-1.4 (-2.5, -0.4)	2.6 (1.3, 4.0)	0.9 (-0.6, 2.4)	0.8 (-0.5, 2.2)
Kyrgyzstan	0.0 (-0.3, 0.4)	0.0 (-0.4, 0.5)	-0.8 (-1.1, -0.5)	-2.7 (-3.7, -1.8)	-2.5 (-3.5, -1.5)	-3.5 (-4.4, -2.6)	-1.3 (-1.7, -0.9)	-1.3 (-1.7, -0.9)	-1.9 (-2.4, -1.4)	-1.9 (-2.6, -1.3)	-2.5 (-3.2, -1.9)	-3.2 (-4.0, -2.4)
Lao People's Democratic Republic	0.8 (0.6, 0.9)	0.6 (0.5, 0.7)	0.3 (0.3, 0.4)	-2.1 (-2.2, -2.1)	-2.0 (-2.0, -1.9)	-2.6 (-2.6, -2.5)	-1.0 (-1.2, -0.8)	-0.9 (-1.1, -0.8)	-1.3 (-1.5, -1.1)	0.7 (0.6, 0.8)	0.4 (0.3, 0.5)	0.1 (0.0, 0.2)
Latvia	-0.7 (-1.3, -0.2)	-0.8 (-1.6, 0.0)	-1.2 (-2.0, -0.5)	-0.7 (-1.8, 0.4)	-2.1 (-3.7, -0.6)	-2.4 (-3.8, -0.8)	-0.2 (-0.9, 0.4)	-2.5 (-3.2, -1.9)	-2.9 (-3.6, -2.3)	1.0 (-0.2, 2.2)	-0.3 (-1.4, 0.9)	-0.5 (-1.6, 0.7)
Lebanon	0.0 (-0.1, 0.1)	-0.3 (-0.4, -0.2)	-0.3 (-0.4, -0.2)	0.6 (0.5, 0.7)	-1.8 (-1.9, -1.7)	-1.8 (-1.9, -1.7)	3.1 (2.8, 3.3)	-1.0 (-1.1, -1.0)	-1.1 (-1.2, -0.9)	3.2 (2.8, 3.5)	0.4 (0.1, 0.7)	0.5 (0.3, 0.8)
Lesotho	1.9 (1.9, 2.0)	1.6 (1.6, 1.7)	1.9 (1.8, 1.9)	2.2 (2.1, 2.4)	2.2 (2.1, 2.4)	2.2 (2.1, 2.4)	1.7 (1.5, 1.8)	1.7 (1.6, 1.8)	1.7 (1.5, 1.9)	2.8 (2.6, 3.0)	2.7 (2.5, 2.8)	2.8 (2.6, 3.0)

Liberia	-0.8 (-1.1, -0.5)	-0.7 (-1.1, -0.3)	-1.5 (-1.8, -1.2)	-0.7 (-1.0, -0.5)	-0.6 (-0.9, -0.3)	-1.0 (-1.3, -0.8)	-1.4 (-1.8, -1.0)	-1.2 (-1.7, -0.7)	-1.5 (-1.9, -1.0)	0.6 (0.3, 0.8)	0.3 (0.0, 0.6)	0.2 (-0.1, 0.5)
Libya	0.0 (-0.1, 0.1)	-0.2 (-0.4, -0.1)	-0.4 (-0.5, -0.2)	-1.5 (-1.9, -1.0)	-1.6 (-2.0, -1.1)	-1.7 (-2.3, -1.2)	-0.3 (-0.6, -0.1)	-0.7 (-0.9, -0.5)	-1.2 (-1.6, -0.7)	2.0 (1.4, 2.6)	0.8 (0.4, 1.5)	0.9 (0.3, 1.5)
Lithuania	0.0 (-0.4, 0.4)	0.0 (-0.4, 0.4)	-0.9 (-1.4, -0.4)	-2.7 (-3.6, -1.7)	-3.7 (-4.5, -2.8)	-4.0 (-5.8, -2.1)	-1.6 (-2.4, -0.8)	-3.2 (-3.9, -2.4)	-3.5 (-4.2, -2.8)	1.1 (-0.8, 3.1)	0.8 (-1.8, 3.3)	0.5 (-0.5, 1.6)
Luxembourg	0.3 (0.1, 0.5)	0.3 (0.2, 0.4)	-0.2 (-0.2, -0.1)	2.5 (1.9, 3.1)	-4.0 (-4.2, -3.7)	-3.8 (-4.1, -3.6)	3.3 (2.8, 3.7)	-2.0 (-2.2, -1.8)	-1.8 (-2.1, -1.6)	0.6 (0.2, 0.9)	-1.1 (-1.5, -0.8)	-1.2 (-1.5, -1.0)
Madagascar	0.2 (0.1, 0.3)	0.1 (0.0, 0.2)	-0.3 (-0.5, 0.0)	-1.3 (-1.6, -1.0)	-1.1 (-1.3, -0.9)	-1.8 (-2.1, -1.4)	-0.8 (-1.2, -0.5)	-0.7 (-1.1, -0.3)	-0.9 (-1.4, -0.4)	1.9 (1.7, 2.0)	1.7 (1.6, 1.9)	1.7 (1.6, 1.9)
Malawi	0.4 (0.2, 0.6)	0.2 (0.1, 0.3)	0.1 (-0.2, 0.3)	-2.3 (-2.6, -1.9)	-2.0 (-2.3, -1.6)	-2.9 (-3.4, -2.3)	-0.6 (-1.0, -0.3)	-0.5 (-0.8, -0.2)	-0.7 (-1.1, -0.3)	0.8 (0.5, 1.0)	0.6 (0.5, 0.8)	0.5 (0.3, 0.7)
Malaysia	0.6 (0.5, 0.7)	0.4 (0.3, 0.5)	0.2 (0.1, 0.2)	-0.8 (-1.5, -0.1)	-1.0 (-2.0, -0.1)	-1.2 (-1.8, -0.6)	-0.3 (-0.6, 0.1)	-0.9 (-1.1, -0.7)	-1.2 (-1.6, -0.7)	2.1 (1.8, 2.4)	1.0 (0.2, 1.8)	0.8 (0.6, 1.1)
Maldives	-0.1 (-0.3, 0.0)	-0.3 (-0.5, -0.1)	-0.6 (-0.8, -0.4)	-2.8 (-3.2, -2.4)	-3.4 (-3.7, -3.1)	-4.3 (-4.5, -4.1)	-0.7 (-1.2, -0.2)	-2.1 (-2.6, -1.7)	-2.4 (-2.9, -1.9)	1.7 (1.3, 2.1)	-0.2 (-0.4, 0.1)	-0.5 (-0.8, -0.3)
Mali	0.3 (-0.2, 0.7)	0.2 (-0.2, 0.6)	0.0 (-0.6, 0.5)	-0.6 (-0.9, -0.3)	-0.5 (-0.8, -0.2)	-0.7 (-1.1, -0.4)	-0.2 (-0.7, 0.3)	-0.1 (-0.7, 0.4)	-0.2 (-0.8, 0.4)	0.4 (0.3, 0.5)	0.2 (0.1, 0.3)	0.1 (0.0, 0.2)
Malta	0.5 (0.3, 0.7)	0.1 (-0.1, 0.3)	0.2 (0.1, 0.3)	1.2 (0.8, 1.5)	-4.0 (-4.2, -3.8)	-3.6 (-3.9, -3.4)	3.5 (2.9, 4.1)	-1.6 (-1.7, -1.4)	-1.0 (-1.3, -0.7)	0.4 (0.2, 0.6)	-1.2 (-1.4, -0.9)	-1.2 (-1.5, -1.0)
Marshall Islands	0.5 (0.4, 0.5)	0.2 (0.1, 0.4)	0.3 (0.2, 0.3)	-0.9 (-1.0, -0.9)	-1.0 (-1.0, -0.9)	-0.8 (-0.9, -0.8)	-0.1 (-0.2, -0.1)	-0.2 (-0.2, -0.1)	-0.1 (-0.1, 0.0)	-0.3 (-0.4, -0.1)	-0.6 (-0.8, -0.4)	-0.4 (-0.5, -0.2)
Mauritania	-0.3 (-0.5, -0.1)	-0.4 (-0.6, -0.2)	-0.6 (-0.9, -0.4)	-1.0 (-1.2, -0.9)	-0.9 (-1.0, -0.8)	-1.3 (-1.4, -1.1)	-0.6 (-0.9, -0.3)	-0.5 (-0.6, -0.4)	-0.6 (-0.9, -0.3)	0.1 (0.0, 0.3)	-0.3 (-0.5, -0.1)	-0.5 (-0.7, -0.3)

Mauritius	2.2 (1.2, 3.4)	2.0 (0.9, 3.1)	1.9 (1.0, 2.9)	1.3 (0.1, 2.6)	1.2 (-0.1, 2.4)	0.7 (-0.5, 2.0)	1.2 (0.2, 2.3)	0.7 (-0.4, 1.8)	0.6 (-0.5, 1.7)	1.2 (0.3, 2.1)	0.1 (-0.9, 1.0)	0.4 (-0.4, 1.1)
Mexico	1.0 (0.8, 1.2)	0.9 (0.7, 1.2)	0.6 (0.4, 0.8)	-1.2 (-1.7, -0.8)	-1.3 (-1.5, -1.1)	-1.6 (-2.1, -1.1)	1.3 (1.1, 1.4)	0.8 (0.5, 1.0)	0.5 (0.3, 0.7)	1.8 (1.4, 2.1)	0.6 (0.4, 0.7)	0.4 (0.2, 0.6)
Micronesia (Federated States of)	0.2 (0.1, 0.2)	-0.1 (-0.1, 0.0)	-0.1 (-0.1, 0.0)	-1.3 (-1.4, -1.2)	-1.3 (-1.3, -1.2)	-1.4 (-1.5, -1.3)	-0.4 (-0.5, -0.4)	-0.4 (-0.5, -0.4)	-0.5 (-0.6, -0.5)	0.5 (0.4, 0.7)	-0.1 (-0.1, 0.0)	0.1 (0.0, 0.1)
Monaco	1.1 (1.1, 1.2)	1.0 (0.9, 1.0)	0.5 (0.4, 0.5)	3.0 (2.7, 3.4)	-1.6 (-1.7, -1.4)	-1.4 (-1.6, -1.3)	1.3 (1.1, 1.5)	-1.0 (-1.1, -0.9)	-1.1 (-1.2, -1.0)	1.4 (1.3, 1.5)	0.3 (0.2, 0.5)	0.3 (0.2, 0.4)
Mongolia	0.6 (0.3, 0.8)	0.3 (0.2, 0.5)	0.2 (0.0, 0.4)	-0.5 (-0.8, -0.3)	-0.4 (-0.6, -0.1)	-0.8 (-1.2, -0.5)	-0.8 (-1.2, -0.4)	-0.8 (-1.2, -0.4)	-1.1 (-1.6, -0.6)	1.8 (1.5, 2.1)	0.9 (0.7, 1.2)	0.6 (0.3, 0.9)
Montenegro	0.1 (-0.2, 0.5)	0.0 (-0.3, 0.3)	-0.4 (-0.8, 0.0)	0.8 (0.3, 1.2)	-1.1 (-1.3, -0.8)	-1.5 (-1.8, -1.1)	1.3 (0.6, 2.1)	-1.3 (-1.9, -0.7)	-1.7 (-2.9, -0.5)	3.6 (3.3, 3.9)	2.4 (2.1, 2.8)	2.4 (2.1, 2.8)
Morocco	0.8 (0.7, 1.0)	0.7 (0.5, 0.9)	0.5 (0.4, 0.7)	-1.1 (-1.3, -0.8)	-0.9 (-1.2, -0.6)	-1.5 (-1.7, -1.3)	-0.3 (-0.8, 0.2)	-0.2 (-0.7, 0.3)	-0.7 (-1.3, -0.1)	1.9 (1.4, 2.5)	1.1 (0.5, 1.6)	1.0 (0.7, 1.3)
Mozambique	1.6 (1.3, 1.9)	1.4 (1.1, 1.6)	1.4 (1.0, 1.8)	0.0 (-0.2, 0.2)	0.2 (0.1, 0.4)	-0.4 (-0.7, -0.2)	0.7 (0.1, 1.3)	0.8 (0.2, 1.3)	0.7 (0.0, 1.4)	2.0 (1.9, 2.2)	1.9 (1.7, 2.0)	1.9 (1.8, 2.0)
Myanmar	1.3 (1.2, 1.4)	1.1 (1.0, 1.2)	0.9 (0.7, 1.0)	-2.6 (-2.6, -2.5)	-2.4 (-2.5, -2.4)	-2.9 (-3.0, -2.8)	-0.4 (-0.6, -0.3)	-0.4 (-0.5, -0.3)	-0.7 (-0.9, -0.4)	1.1 (1.0, 1.2)	0.6 (0.5, 0.7)	0.3 (0.2, 0.4)
Namibia	1.2 (0.9, 1.6)	1.0 (0.7, 1.3)	1.1 (0.6, 1.5)	0.2 (-0.1, 0.4)	0.2 (0.0, 0.4)	0.1 (-0.2, 0.3)	1.0 (0.4, 1.7)	0.9 (0.3, 1.4)	1.0 (0.2, 1.7)	1.8 (1.6, 2.1)	1.3 (1.0, 1.5)	1.1 (0.7, 1.5)
Nauru	-0.2 (-0.2, -0.1)	-0.4 (-0.5, -0.3)	-0.4 (-0.5, -0.3)	-1.1 (-1.2, -1.0)	-1.2 (-1.3, -1.0)	-1.1 (-1.2, -1.0)	-0.2 (-0.3, -0.1)	-0.2 (-0.4, -0.1)	-0.3 (-0.5, -0.1)	0.4 (0.3, 0.5)	-0.2 (-0.4, -0.1)	-0.1 (-0.2, 0.0)

Nepal	0.5 (0.4, 0.6)	0.5 (0.4, 0.5)	0.0 (-0.2, 0.1)	-1.9 (-1.9, -1.8)	-1.6 (-1.7, -1.5)	-2.6 (-2.7, -2.5)	-1.5 (-1.8, -1.3)	-1.4 (-1.6, -1.1)	-1.8 (-2.3, -1.3)	1.8 (1.7, 1.9)	1.3 (1.3, 1.4)	1.1 (1.0, 1.1)
Netherlands	0.6 (0.2, 1.0)	0.7 (0.3, 1.0)	-0.1 (-0.6, 0.4)	0.8 (-0.1, 1.6)	-4.8 (-5.5, -4.1)	-4.7 (-5.3, -4.0)	1.4 (0.8, 2.1)	-1.7 (-2.4, -1.1)	-1.8 (-2.4, -1.1)	-2.8 (-4.7, -0.9)	-4.2 (-6.1, -2.4)	-4.1 (-5.7, -2.6)
New Zealand	-0.1 (-0.3, 0.1)	0.0 (-0.2, 0.3)	-0.3 (-0.5, -0.1)	-0.9 (-1.3, -0.5)	-4.4 (-4.8, -4.1)	-4.4 (-4.8, -4.1)	1.3 (0.6, 2.1)	-2.1 (-2.4, -1.7)	-2.1 (-2.6, -1.6)	1.2 (0.7, 1.8)	0.0 (-0.6, 0.6)	-0.1 (-0.6, 0.4)
Nicaragua	0.8 (0.2, 1.5)	0.8 (0.1, 1.5)	0.1 (-0.1, 0.3)	0.2 (-0.9, 1.3)	0.2 (-0.9, 1.3)	-0.6 (-1.7, 0.4)	1.4 (1.1, 1.6)	1.0 (0.7, 1.3)	0.4 (0.2, 0.6)	1.3 (0.7, 1.9)	-0.1 (-1.4, 1.2)	-0.4 (-0.9, 0.1)
Niger	-0.1 (-0.4, 0.2)	-0.2 (-0.5, 0.1)	-0.6 (-0.9, -0.2)	-0.5 (-0.8, -0.3)	-0.4 (-0.6, -0.2)	-0.7 (-1.1, -0.4)	0.0 (-0.4, 0.4)	0.0 (-0.4, 0.4)	0.1 (-0.4, 0.6)	0.8 (0.7, 0.9)	0.6 (0.5, 0.7)	0.5 (0.4, 0.7)
Nigeria	0.6 (0.3, 0.8)	0.4 (0.1, 0.6)	0.1 (-0.1, 0.4)	0.0 (-0.1, 0.1)	0.1 (0.0, 0.2)	-0.3 (-0.4, -0.1)	-0.1 (-0.2, 0.1)	-0.1 (-0.3, 0.2)	-0.2 (-0.3, 0.0)	1.1 (0.9, 1.3)	0.8 (0.6, 0.9)	0.8 (0.6, 1.0)
Niue	0.1 (0.0, 0.1)	-0.2 (-0.2, -0.1)	-0.2 (-0.2, -0.1)	-1.3 (-1.5, -1.1)	-1.5 (-1.7, -1.4)	-1.7 (-1.8, -1.5)	0.2 (0.1, 0.3)	-0.4 (-0.5, -0.4)	-0.4 (-0.5, -0.3)	0.9 (0.7, 1.1)	-0.3 (-0.4, -0.2)	-0.3 (-0.4, -0.1)
North Macedonia	-0.3 (-0.5, -0.1)	-0.5 (-0.7, -0.4)	-0.5 (-0.7, -0.4)	-0.2 (-0.6, 0.1)	-1.5 (-1.8, -1.2)	-1.7 (-2.1, -1.4)	1.4 (0.9, 2.0)	-1.0 (-1.5, -0.5)	-1.1 (-1.7, -0.5)	4.8 (4.5, 5.2)	3.2 (2.9, 3.5)	3.3 (3.0, 3.6)
Northern Mariana Islands	-2.4 (-2.5, -2.2)	-2.7 (-2.8, -2.5)	-2.4 (-2.6, -2.2)	-3.2 (-4.3, -2.1)	-3.5 (-4.2, -2.8)	-3.4 (-4.1, -2.8)	-1.7 (-2.5, -0.9)	-2.4 (-2.7, -2.1)	-2.4 (-2.7, -2.1)	-0.5 (-1.0, 0.0)	-1.7 (-1.9, -1.5)	-1.0 (-1.2, -0.8)
Norway	-0.7 (-0.9, -0.5)	-0.3 (-0.5, -0.1)	-1.1 (-1.3, -0.9)	0.6 (0.4, 0.9)	-4.4 (-4.9, -4.0)	-4.6 (-5.0, -4.2)	1.0 (0.3, 1.6)	-2.1 (-2.4, -1.8)	-2.3 (-2.6, -2.1)	0.5 (0.3, 0.7)	-0.7 (-0.8, -0.6)	-1.0 (-1.1, -0.8)
Oman	0.5 (0.4, 0.6)	0.3 (0.2, 0.5)	0.2 (0.0, 0.3)	-1.0 (-1.3, -0.8)	-2.0 (-2.3, -1.6)	-2.5 (-2.8, -2.2)	1.9 (1.5, 2.2)	-0.6 (-0.7, -0.5)	-0.8 (-1.1, -0.6)	3.0 (2.6, 3.5)	1.2 (1.0, 1.5)	0.8 (0.6, 1.1)
Pakistan	1.4 (1.2, 1.5)	1.0 (0.8, 1.1)	1.1 (1.0, 1.3)	-0.8 (-0.9, -0.6)	-0.8 (-0.9, -0.7)	-0.9 (-1.1, -0.8)	1.1 (0.8, 1.4)	1.0 (0.7, 1.2)	1.1 (0.7, 1.5)	1.3 (1.2, 1.4)	0.9 (0.9, 1.0)	0.9 (0.9, 1.0)

Palau	0.2 (0.1, 0.2)	0.0 (-0.1, 0.0)	-0.1 (-0.2, 0.0)	-0.5 (-0.6, -0.4)	-0.7 (-0.7, -0.6)	-0.8 (-1.0, -0.7)	0.4 (0.3, 0.5)	-0.2 (-0.3, -0.1)	-0.2 (-0.4, -0.1)	1.0 (0.8, 1.1)	0.1 (0.1, 0.2)	0.1 (0.0, 0.2)
Palestine	1.2 (1.1, 1.3)	1.1 (0.9, 1.3)	0.8 (0.7, 1.0)	-0.5 (-0.7, -0.3)	-0.6 (-0.7, -0.4)	-1.0 (-1.4, -0.7)	1.2 (0.8, 1.5)	0.7 (0.4, 1.1)	0.5 (0.0, 0.9)	2.9 (2.6, 3.1)	1.8 (1.2, 2.3)	1.5 (1.0, 2.0)
Panama	0.9 (0.6, 1.2)	0.7 (0.4, 0.9)	0.5 (0.2, 0.8)	-1.0 (-1.9, -0.1)	-1.3 (-2.3, -0.4)	-1.5 (-2.3, -0.7)	0.8 (0.7, 1.0)	-0.1 (-0.4, 0.2)	-0.3 (-0.8, 0.1)	1.1 (0.5, 1.7)	0.0 (-0.7, 0.7)	-0.4 (-1.0, 0.3)
Papua New Guinea	0.4 (0.1, 0.7)	0.3 (0.2, 0.4)	0.2 (-0.2, 0.6)	-0.8 (-1.1, -0.5)	-0.7 (-1.0, -0.5)	-0.9 (-1.2, -0.5)	0.1 (-0.5, 0.6)	0.1 (-0.4, 0.6)	0.0 (-0.5, 0.6)	0.2 (0.1, 0.3)	0.0 (-0.1, 0.1)	0.0 (-0.1, 0.2)
Paraguay	2.4 (2.1, 2.7)	2.2 (2.0, 2.5)	1.8 (1.5, 2.0)	0.9 (0.3, 1.6)	0.9 (0.3, 1.6)	0.5 (-0.2, 1.1)	0.9 (0.5, 1.3)	0.7 (0.3, 1.1)	0.2 (-0.2, 0.6)	3.6 (3.3, 3.9)	2.8 (2.5, 3.1)	2.7 (2.4, 3.0)
Peru	1.1 (0.8, 1.4)	1.0 (0.8, 1.3)	0.6 (0.4, 0.9)	-0.5 (-1.6, 0.6)	-0.5 (-1.6, 0.6)	-1.0 (-2.1, 0.0)	0.4 (-0.1, 0.9)	-0.1 (-0.6, 0.4)	-0.3 (-0.9, 0.2)	2.5 (1.4, 3.6)	1.0 (-0.2, 2.3)	0.8 (-0.6, 2.1)
Philippines	0.2 (0.0, 0.4)	-0.2 (-0.3, 0.0)	0.1 (-0.1, 0.3)	-1.6 (-1.8, -1.4)	-1.7 (-1.9, -1.5)	-1.6 (-1.8, -1.4)	-0.3 (-0.5, -0.2)	-0.5 (-0.7, -0.3)	-0.4 (-0.6, -0.2)	-0.2 (-0.8, 0.3)	-0.7 (-1.0, -0.4)	-0.4 (-0.7, -0.1)
Poland	0.9 (0.8, 1.0)	0.9 (0.8, 1.0)	0.0 (-0.1, 0.1)	-1.2 (-1.5, -0.8)	-3.2 (-3.6, -2.7)	-3.9 (-4.4, -3.4)	2.2 (1.9, 2.6)	-2.1 (-2.5, -1.7)	-2.3 (-2.7, -1.9)	6.6 (6.3, 7.0)	4.6 (4.2, 4.9)	4.4 (4.0, 4.8)
Portugal	0.8 (0.6, 1.0)	0.8 (0.5, 1.0)	-0.1 (-0.6, 0.3)	1.7 (1.2, 2.2)	-4.1 (-4.5, -3.6)	-4.2 (-4.8, -3.6)	4.4 (4.0, 4.8)	-2.3 (-2.9, -1.7)	-2.5 (-2.9, -2.1)	2.9 (2.2, 3.7)	0.8 (0.1, 1.4)	0.6 (0.0, 1.3)
Puerto Rico	-0.2 (-0.7, 0.3)	-0.3 (-0.6, 0.0)	-0.7 (-1.1, -0.4)	-1.5 (-2.9, 0.0)	-3.5 (-4.7, -2.3)	-3.6 (-4.8, -2.4)	1.9 (1.6, 2.1)	-1.7 (-1.9, -1.4)	-1.9 (-2.2, -1.6)	0.0 (-0.7, 0.8)	-1.6 (-2.4, -0.8)	-1.4 (-2.2, -0.6)
Qatar	0.5 (0.0, 1.0)	0.4 (-0.2, 1.1)	-0.4 (-0.7, -0.1)	1.0 (-1.1, 3.1)	-0.6 (-3.6, 2.5)	-1.8 (-2.9, -0.7)	2.4 (1.7, 3.1)	-0.8 (-1.4, -0.2)	-1.8 (-2.5, -1.1)	2.6 (0.8, 4.3)	1.1 (0.8, 1.5)	0.1 (-1.7, 2.0)
Republic of Korea	0.8 (0.4, 1.1)	0.9 (0.6, 1.3)	-0.3 (-0.6, 0.1)	3.2 (2.5, 3.8)	-3.0 (-3.2, -2.8)	-3.7 (-4.0, -3.4)	6.2 (5.8, 6.7)	-1.4 (-1.7, -1.0)	-1.6 (-1.9, -1.3)	4.0 (3.7, 4.4)	0.5 (0.2, 0.8)	0.6 (0.3, 0.8)

Republic of Moldova	-1.1 (-1.6, -0.7)	-1.2 (-1.6, -0.7)	-1.6 (-2.0, -1.2)	-2.6 (-4.4, -0.7)	-2.8 (-4.9, -0.7)	-3.0 (-4.8, -1.2)	-2.8 (-3.3, -2.4)	-3.6 (-4.1, -3.2)	-3.9 (-4.4, -3.4)	0.0 (-1.2, 1.1)	-0.9 (-2.2, 0.4)	-1.2 (-2.5, 0.1)
Romania	0.9 (0.7, 1.1)	0.9 (0.7, 1.2)	0.2 (0.0, 0.3)	-0.7 (-1.1, -0.2)	-1.9 (-2.4, -1.4)	-2.7 (-3.1, -2.2)	1.0 (0.6, 1.3)	-1.4 (-1.6, -1.2)	-2.1 (-2.6, -1.7)	3.1 (2.6, 3.7)	1.6 (1.0, 2.2)	1.1 (0.5, 1.8)
Russian Federation	-1.1 (-1.7, -0.5)	-1.2 (-1.8, -0.6)	-1.6 (-2.2, -1.0)	0.9 (-0.8, 2.7)	-0.2 (-1.6, 1.2)	-0.8 (-2.3, 0.7)	1.0 (0.4, 1.7)	-1.3 (-1.8, -0.8)	-1.7 (-2.2, -1.2)	2.4 (1.3, 3.5)	1.1 (0.1, 2.1)	0.8 (-0.1, 1.8)
Rwanda	1.3 (1.1, 1.5)	1.1 (0.9, 1.3)	1.1 (0.8, 1.4)	-1.8 (-2.1, -1.4)	-1.6 (-2.1, -1.2)	-2.0 (-2.5, -1.4)	0.0 (-0.5, 0.4)	0.1 (-0.4, 0.7)	0.0 (-0.5, 0.5)	1.4 (1.1, 1.7)	1.1 (0.9, 1.3)	0.9 (0.6, 1.1)
Saint Kitts and Nevis	0.8 (0.1, 1.5)	0.5 (0.0, 1.0)	0.5 (-0.3, 1.2)	-1.6 (-1.9, -1.3)	-1.8 (-2.1, -1.5)	-2.1 (-2.5, -1.7)	0.3 (-0.4, 1.1)	-0.5 (-1.2, 0.2)	-0.6 (-1.4, 0.1)	1.6 (1.0, 2.1)	0.5 (-0.2, 1.1)	0.5 (0.0, 0.9)
Saint Lucia	1.0 (0.9, 1.2)	0.8 (0.5, 1.1)	0.7 (0.4, 1.1)	-2.0 (-2.2, -1.7)	-2.1 (-2.3, -1.8)	-2.1 (-2.3, -2.0)	0.0 (-0.3, 0.4)	-0.2 (-0.6, 0.1)	-0.4 (-0.8, 0.0)	0.8 (0.5, 1.1)	0.0 (-0.4, 0.4)	-0.1 (-0.3, 0.2)
Saint Vincent and the Grenadines	2.2 (2.1, 2.4)	2.1 (1.8, 2.4)	1.7 (1.2, 2.2)	-3.6 (-4.0, -3.3)	-3.7 (-4.1, -3.3)	-3.8 (-4.3, -3.3)	0.8 (0.2, 1.4)	0.8 (0.2, 1.4)	0.4 (-0.3, 1.0)	1.8 (1.5, 2.1)	1.4 (1.2, 1.6)	1.4 (1.2, 1.6)
Samoa	0.0 (-0.1, 0.1)	-0.3 (-0.3, -0.2)	-0.4 (-0.4, -0.3)	-0.9 (-0.9, -0.8)	-0.9 (-1.0, -0.9)	-0.9 (-0.9, -0.9)	-0.5 (-0.6, -0.4)	-0.6 (-0.6, -0.6)	-0.7 (-0.8, -0.7)	0.2 (-0.1, 0.6)	-0.4 (-0.6, -0.2)	-0.2 (-0.4, -0.1)
San Marino	0.6 (0.5, 0.7)	0.5 (0.4, 0.6)	0.3 (0.3, 0.4)	2.9 (2.4, 3.5)	-1.9 (-2.1, -1.8)	-1.7 (-1.9, -1.4)	2.5 (2.3, 2.6)	-0.1 (-0.2, 0.0)	0.0 (-0.2, 0.1)	1.5 (1.4, 1.7)	0.0 (-0.1, 0.2)	0.3 (0.2, 0.5)
Sao Tome and Principe	0.4 (0.2, 0.6)	0.4 (0.0, 0.8)	0.1 (-0.5, 0.7)	0.4 (0.1, 0.7)	0.5 (0.2, 0.7)	0.2 (-0.2, 0.6)	-0.7 (-1.0, -0.5)	-0.7 (-0.9, -0.5)	-1.0 (-1.3, -0.7)	1.6 (1.5, 1.8)	1.2 (1.0, 1.4)	1.1 (1.0, 1.3)

Saudi Arabia	0.6 (0.4, 0.7)	-0.1 (-0.2, 0.0)	-0.1 (-0.2, 0.1)	0.1 (0.1, 0.2)	-1.3 (-1.4, -1.2)	-1.1 (-1.2, -1.0)	1.5 (1.3, 1.7)	-1.2 (-1.3, -1.2)	-1.4 (-1.5, -1.3)	3.2 (2.9, 3.5)	0.9 (0.7, 1.0)	1.1 (1.0, 1.2)
Senegal	0.1 (-1.0, 1.3)	0.1 (-0.9, 1.1)	-0.5 (-1.7, 0.8)	-0.5 (-1.1, 0.1)	-0.4 (-0.9, 0.1)	-0.6 (-1.4, 0.1)	-0.1 (-1.4, 1.2)	-0.1 (-1.3, 1.1)	-0.2 (-1.6, 1.2)	0.8 (0.2, 1.4)	0.6 (0.1, 1.1)	0.5 (-0.1, 1.1)
Serbia	0.5 (0.3, 0.8)	0.5 (0.3, 0.6)	-0.1 (-0.4, 0.2)	1.6 (1.3, 1.8)	0.0 (-0.3, 0.3)	-0.5 (-1.1, 0.0)	2.4 (1.8, 2.9)	-0.7 (-1.0, -0.4)	-1.3 (-1.6, -1.1)	3.5 (2.9, 4.1)	1.7 (1.2, 2.4)	1.4 (0.8, 2.0)
Seychelles	0.5 (0.3, 0.8)	0.4 (0.2, 0.5)	0.2 (0.0, 0.5)	-1.1 (-1.5, -0.7)	-1.2 (-1.6, -0.8)	-1.4 (-1.6, -1.1)	0.7 (-1.1, 2.5)	0.5 (-1.0, 2.0)	0.7 (-1.2, 2.5)	0.9 (0.6, 1.2)	0.0 (-0.2, 0.2)	-0.2 (-0.4, 0.0)
Sierra Leone	0.6 (0.2, 0.9)	0.4 (0.1, 0.7)	0.1 (-0.3, 0.5)	-0.1 (-0.3, 0.0)	-0.1 (-0.3, 0.0)	-0.1 (-0.3, 0.0)	0.2 (-0.2, 0.5)	0.2 (-0.2, 0.5)	0.2 (-0.2, 0.6)	1.0 (0.9, 1.1)	0.8 (0.6, 0.9)	0.7 (0.6, 0.8)
Singapore	-0.6 (-1.2, -0.1)	-0.6 (-1.1, -0.1)	-1.3 (-1.9, -0.7)	0.9 (-0.6, 2.5)	-4.9 (-5.5, -4.3)	-5.0 (-5.6, -4.3)	3.6 (2.7, 4.6)	-2.3 (-2.7, -1.9)	-2.2 (-2.7, -1.8)	1.7 (1.0, 2.4)	-0.2 (-1.1, 0.7)	-0.4 (-0.8, 0.0)
Slovakia	-0.3 (-0.5, -0.1)	-0.5 (-0.7, -0.2)	-0.7 (-0.9, -0.4)	-1.6 (-2.2, -1.0)	-4.1 (-4.6, -3.6)	-4.3 (-4.8, -3.8)	2.6 (2.2, 3.0)	-1.3 (-1.5, -1.1)	-1.4 (-1.6, -1.1)	1.4 (1.1, 1.8)	-0.2 (-0.5, 0.1)	-0.2 (-0.5, 0.2)
Slovenia	1.7 (1.3, 2.1)	1.7 (1.3, 2.1)	0.9 (0.3, 1.4)	2.6 (2.4, 2.9)	-2.9 (-3.2, -2.6)	-3.3 (-3.7, -2.9)	3.2 (2.9, 3.5)	-2.4 (-2.9, -1.8)	-2.3 (-2.8, -1.8)	0.8 (0.0, 1.7)	-1.0 (-1.4, -0.7)	-1.0 (-1.8, -0.3)
Solomon Islands	0.7 (0.5, 0.8)	0.4 (0.3, 0.6)	0.4 (0.3, 0.6)	-0.6 (-0.8, -0.4)	-0.6 (-0.8, -0.3)	-0.6 (-0.8, -0.4)	0.2 (-0.1, 0.4)	0.1 (-0.1, 0.3)	0.2 (0.0, 0.5)	0.7 (0.6, 0.9)	0.2 (0.0, 0.4)	0.3 (0.1, 0.5)
Somalia	0.8 (0.7, 1.0)	0.6 (0.5, 0.7)	0.7 (0.5, 1.0)	-0.6 (-0.7, -0.5)	-0.4 (-0.5, -0.3)	-0.7 (-0.9, -0.6)	0.6 (0.3, 1.0)	0.6 (0.3, 1.0)	0.7 (0.3, 1.0)	1.5 (1.4, 1.6)	1.5 (1.5, 1.6)	1.5 (1.4, 1.6)
South Africa	0.4 (0.3, 0.5)	0.2 (0.1, 0.3)	0.1 (-0.2, 0.3)	-2.1 (-2.7, -1.5)	-2.0 (-2.7, -1.4)	-2.3 (-2.7, -1.9)	-0.6 (-1.0, -0.2)	-0.7 (-0.9, -0.5)	-1.0 (-1.5, -0.6)	0.6 (0.0, 1.2)	0.2 (-0.4, 0.8)	-0.2 (-1.0, 0.5)
South Sudan	0.6 (0.4, 0.8)	0.4 (0.2, 0.6)	0.2 (0.0, 0.5)	-0.9 (-1.1, -0.7)	-0.7 (-0.9, -0.6)	-1.3 (-1.5, -1.1)	0.1 (-0.2, 0.4)	0.1 (-0.2, 0.3)	0.0 (-0.3, 0.3)	0.9 (0.8, 1.1)	0.8 (0.7, 0.9)	0.9 (0.7, 1.1)

Spain	1.7 (1.5, 1.8)	1.6 (1.4, 1.7)	0.7 (0.5, 0.8)	2.3 (1.9, 2.7)	-4.2 (-4.5, -3.9)	-4.5 (-4.8, -4.1)	3.7 (3.3, 4.0)	-1.5 (-1.7, -1.3)	-1.4 (-1.5, -1.2)	1.9 (1.5, 2.3)	0.3 (0.1, 0.5)	0.0 (-0.2, 0.3)
Sri Lanka	0.7 (0.3, 1.1)	0.5 (0.2, 0.8)	0.2 (-0.4, 0.8)	-1.0 (-1.7, -0.3)	-1.5 (-2.0, -0.9)	-1.8 (-2.5, -1.0)	0.1 (-0.5, 0.7)	-1.0 (-1.5, -0.5)	-1.3 (-2.0, -0.5)	1.7 (1.1, 2.3)	0.0 (-0.8, 0.8)	0.0 (-0.6, 0.6)
Sudan	1.2 (1.1, 1.3)	0.9 (0.8, 1.1)	0.9 (0.8, 1.0)	-1.5 (-1.6, -1.5)	-1.4 (-1.4, -1.3)	-2.2 (-2.3, -2.0)	0.3 (0.1, 0.5)	0.3 (0.1, 0.4)	0.1 (-0.2, 0.3)	2.2 (2.2, 2.3)	1.6 (1.5, 1.6)	1.3 (1.3, 1.4)
Suriname	0.9 (0.5, 1.3)	0.9 (0.2, 1.5)	0.5 (0.1, 0.9)	-0.8 (-1.5, -0.1)	-0.8 (-1.4, -0.1)	-0.9 (-1.8, 0.0)	-0.3 (-1.0, 0.4)	-0.3 (-1.1, 0.4)	-0.4 (-0.9, 0.2)	2.5 (1.7, 3.3)	1.9 (1.2, 2.7)	2.0 (1.1, 2.8)
Sweden	0.4 (0.3, 0.6)	0.3 (0.1, 0.5)	-0.2 (-0.4, 0.0)	0.2 (-0.7, 1.2)	-4.5 (-5.3, -3.8)	-4.6 (-5.4, -3.8)	0.3 (-0.2, 0.9)	-2.6 (-3.1, -2.0)	-2.4 (-2.7, -2.1)	0.8 (0.7, 0.9)	0.0 (-0.2, 0.1)	-0.3 (-0.6, 0.1)
Switzerland	-0.8 (-1.3, -0.3)	-1.1 (-1.6, -0.5)	-1.8 (-2.1, -1.5)	-0.3 (-0.8, 0.1)	-5.1 (-5.3, -4.8)	-5.3 (-5.7, -4.9)	0.3 (-0.2, 0.9)	-3.5 (-4.0, -3.0)	-3.7 (-4.2, -3.2)	-1.5 (-2.0, -1.0)	-2.4 (-2.8, -2.1)	-2.6 (-3.1, -2.2)
Syrian Arab Republic	-0.1 (-0.2, 0.1)	-0.3 (-0.4, -0.2)	-0.4 (-0.5, -0.2)	-2.4 (-2.7, -2.0)	-2.5 (-2.7, -2.2)	-2.8 (-3.2, -2.5)	-0.2 (-0.6, 0.2)	-0.9 (-1.2, -0.6)	-1.1 (-1.5, -0.6)	1.6 (1.3, 1.9)	0.3 (0.1, 0.4)	0.1 (-0.1, 0.3)
Taiwan (Province of China)	3.8 (2.6, 4.9)	3.8 (2.6, 4.9)	2.9 (2.3, 3.5)	2.5 (1.9, 3.2)	-1.0 (-1.4, -0.6)	-1.7 (-2.2, -1.2)	6.5 (5.6, 7.3)	1.0 (0.2, 1.8)	1.2 (0.4, 2.0)	1.7 (1.2, 2.1)	-0.1 (-0.6, 0.4)	-0.3 (-0.8, 0.1)
Tajikistan	-0.4 (-0.6, -0.2)	-0.4 (-0.6, -0.3)	-0.9 (-1.1, -0.8)	-2.6 (-2.9, -2.3)	-2.4 (-2.8, -1.9)	-3.3 (-3.5, -3.1)	-1.2 (-1.6, -0.7)	-1.0 (-1.4, -0.6)	-1.6 (-2.0, -1.2)	1.0 (0.2, 1.7)	0.9 (0.3, 1.5)	0.0 (-0.5, 0.6)
Thailand	3.1 (2.9, 3.4)	3.0 (2.7, 3.3)	2.6 (2.3, 2.9)	1.5 (0.7, 2.3)	1.0 (0.3, 1.8)	1.1 (0.5, 1.6)	2.3 (2.0, 2.6)	1.1 (0.8, 1.4)	1.1 (0.9, 1.4)	2.4 (1.8, 3.1)	1.0 (0.4, 1.7)	1.0 (0.8, 1.3)
Timor-Leste	1.3 (0.9, 1.7)	1.0 (0.7, 1.3)	0.8 (0.4, 1.2)	-1.2 (-1.5, -0.8)	-1.0 (-1.2, -0.7)	-1.6 (-2.0, -1.3)	-0.2 (-0.6, 0.2)	-0.1 (-0.3, 0.2)	-0.5 (-1.0, -0.1)	2.2 (2.0, 2.4)	1.8 (1.7, 2.0)	1.5 (1.3, 1.7)
Togo	0.4 (0.1, 0.7)	0.3 (0.0, 0.7)	-0.1 (-0.5, 0.3)	-0.5 (-0.7, -0.3)	-0.5 (-0.7, -0.3)	-0.6 (-0.8, -0.3)	-0.1 (-0.4, 0.2)	-0.1 (-0.3, 0.2)	-0.1 (-0.4, 0.2)	0.6 (0.4, 0.9)	0.5 (0.2, 0.7)	0.4 (0.1, 0.6)

Tokelau	0.0 (-0.1, 0.1)	-0.2 (-0.3, 0.0)	-0.3 (-0.5, -0.1)	-1.9 (-2.0, -1.7)	-1.9 (-2.0, -1.7)	-2.0 (-2.1, -1.8)	-0.4 (-0.5, -0.2)	-0.5 (-0.6, -0.3)	-0.6 (-0.9, -0.4)	0.6 (0.4, 0.8)	-0.4 (-0.5, -0.3)	-0.3 (-0.4, -0.2)
Tonga	0.6 (0.5, 0.8)	0.4 (0.3, 0.6)	0.5 (0.4, 0.6)	-0.9 (-1.1, -0.7)	-0.9 (-1.1, -0.7)	-0.9 (-1.1, -0.7)	0.2 (0.0, 0.4)	0.2 (-0.1, 0.5)	0.1 (-0.1, 0.3)	0.3 (-0.2, 0.8)	-0.2 (-0.6, 0.2)	-0.1 (-0.4, 0.3)
Trinidad and Tobago	0.1 (-0.2, 0.3)	0.0 (-0.2, 0.2)	-0.2 (-0.4, 0.0)	-2.3 (-3.0, -1.6)	-2.4 (-3.0, -1.7)	-2.3 (-3.0, -1.7)	-1.2 (-1.4, -0.9)	-1.4 (-1.9, -0.8)	-1.5 (-2.1, -0.9)	1.0 (0.3, 1.7)	0.1 (-0.5, 0.8)	0.2 (-0.5, 0.9)
Tunisia	0.2 (0.1, 0.2)	0.0 (0.0, 0.1)	-0.4 (-0.4, -0.3)	-0.6 (-0.8, -0.5)	-1.3 (-1.4, -1.2)	-2.0 (-2.0, -1.9)	0.6 (0.5, 0.7)	-1.2 (-1.3, -1.0)	-1.6 (-1.8, -1.5)	2.5 (2.3, 2.7)	0.6 (0.4, 0.8)	0.7 (0.5, 0.8)
Turkey	-0.5 (-0.6, -0.3)	-0.6 (-0.7, -0.4)	-1.1 (-1.3, -0.9)	-3.5 (-3.9, -3.1)	-4.0 (-4.7, -3.3)	-5.0 (-5.4, -4.5)	-0.3 (-1.1, 0.5)	-2.1 (-2.7, -1.6)	-2.5 (-3.1, -1.8)	1.9 (1.5, 2.3)	0.1 (-0.2, 0.4)	-0.4 (-0.8, 0.0)
Turkmenistan	0.2 (-0.4, 0.9)	0.1 (-0.5, 0.8)	-0.3 (-0.8, 0.2)	-1.3 (-2.2, -0.3)	-1.2 (-2.1, -0.3)	-1.9 (-2.7, -1.0)	-0.8 (-1.2, -0.5)	-1.0 (-1.3, -0.7)	-1.3 (-1.7, -1.0)	1.6 (0.9, 2.4)	0.9 (0.4, 1.4)	0.4 (-0.2, 1.0)
Tuvalu	0.0 (-0.1, 0.1)	-0.2 (-0.2, -0.1)	-0.4 (-0.5, -0.3)	-1.9 (-2.0, -1.7)	-1.8 (-1.9, -1.7)	-2.0 (-2.1, -1.9)	-1.0 (-1.1, -0.8)	-1.0 (-1.1, -0.8)	-1.3 (-1.5, -1.1)	0.1 (0.0, 0.2)	-0.6 (-0.7, -0.5)	-0.4 (-0.5, -0.3)
Uganda	1.1 (0.7, 1.4)	0.8 (0.5, 1.1)	1.0 (0.6, 1.4)	-1.5 (-2.0, -1.1)	-1.5 (-1.8, -1.1)	-1.7 (-2.2, -1.2)	-0.1 (-0.6, 0.5)	-0.1 (-0.6, 0.3)	0.1 (-0.4, 0.7)	1.7 (1.5, 2.0)	1.5 (1.3, 1.7)	1.4 (1.2, 1.6)
Ukraine	-1.5 (-2.3, -0.7)	-1.6 (-2.4, -0.8)	-2.3 (-3.3, -1.3)	-0.3 (-1.4, 0.8)	-1.4 (-2.3, -0.5)	-1.6 (-2.8, -0.5)	-1.3 (-1.9, -0.8)	-2.6 (-3.4, -1.9)	-3.1 (-3.9, -2.3)	0.6 (0.2, 0.9)	-0.3 (-0.9, 0.3)	-0.4 (-1.0, 0.2)
United Arab Emirates	-0.1 (-0.4, 0.2)	-0.2 (-0.6, 0.1)	-0.3 (-0.5, -0.1)	-2.7 (-3.3, -2.1)	-2.8 (-3.5, -2.1)	-2.8 (-3.2, -2.3)	-0.2 (-0.7, 0.3)	-0.6 (-1.1, 0.0)	-0.8 (-1.2, -0.5)	1.3 (0.3, 2.3)	0.6 (-0.5, 1.7)	0.4 (-0.6, 1.4)
United Kingdom	0.1 (-0.1, 0.3)	0.3 (0.2, 0.4)	-0.3 (-0.4, -0.2)	0.2 (-0.2, 0.7)	-4.2 (-4.6, -3.8)	-4.3 (-4.6, -4.0)	1.0 (0.7, 1.2)	-2.0 (-2.2, -1.7)	-2.2 (-2.5, -1.8)	0.4 (0.1, 0.8)	-0.5 (-0.8, -0.2)	-0.7 (-1.0, -0.5)
United Republic of Tanzania	1.5 (1.2, 1.7)	1.2 (1.0, 1.4)	1.3 (0.9, 1.6)	-0.3 (-0.5, -0.1)	-0.2 (-0.3, -0.1)	-0.5 (-0.9, -0.2)	0.8 (0.5, 1.1)	0.8 (0.5, 1.1)	0.8 (0.5, 1.2)	1.8 (1.7, 1.9)	1.5 (1.4, 1.6)	1.5 (1.4, 1.7)

United States of America	0.6 (0.5, 0.7)	0.5 (0.4, 0.6)	0.0 (-0.2, 0.2)	-1.7 (-2.0, -1.4)	-3.8 (-4.1, -3.5)	-4.4 (-4.7, -4.0)	-0.3 (-0.6, -0.1)	-1.0 (-1.2, -0.9)	-1.4 (-1.6, -1.2)	-0.4 (-0.7, 0.0)	-0.8 (-0.9, -0.6)	-1.1 (-1.2, -1.0)
United States Virgin Islands	0.0 (-0.1, 0.1)	-0.1 (-0.3, 0.1)	-0.5 (-0.7, -0.3)	-1.6 (-1.8, -1.3)	-1.7 (-2.0, -1.5)	-2.2 (-2.6, -1.8)	-1.8 (-2.4, -1.2)	-2.2 (-2.8, -1.6)	-2.5 (-3.3, -1.8)	1.1 (0.9, 1.4)	0.4 (0.1, 0.8)	0.4 (0.0, 0.7)
Uruguay	0.1 (-0.1, 0.3)	-0.1 (-0.3, 0.1)	-0.4 (-0.8, 0.0)	-2.2 (-3.0, -1.4)	-2.6 (-3.4, -1.9)	-2.9 (-3.6, -2.2)	-0.4 (-0.8, 0.0)	-1.5 (-1.8, -1.2)	-1.8 (-2.2, -1.4)	1.2 (0.6, 1.8)	0.2 (-0.6, 1.0)	0.2 (-0.3, 0.7)
Uzbekistan	0.1 (-0.1, 0.4)	0.0 (-0.2, 0.2)	-0.2 (-0.5, 0.0)	-0.4 (-0.7, -0.1)	-0.3 (-0.7, 0.2)	-1.0 (-1.5, -0.5)	-0.6 (-0.9, -0.3)	-0.8 (-1.0, -0.6)	-1.0 (-1.2, -0.8)	1.7 (1.3, 2.0)	1.0 (0.5, 1.5)	0.4 (-0.1, 0.9)
Vanuatu	0.8 (0.6, 1.0)	0.5 (0.3, 0.7)	0.6 (0.4, 0.8)	-0.7 (-0.9, -0.4)	-0.7 (-0.9, -0.4)	-0.6 (-0.9, -0.3)	0.5 (0.1, 0.9)	0.4 (0.2, 0.7)	0.5 (0.0, 1.1)	0.2 (-0.1, 0.4)	-0.1 (-0.4, 0.1)	0.2 (-0.1, 0.5)
Venezuela (Bolivarian Republic of)	0.8 (0.4, 1.2)	0.6 (0.2, 1.1)	0.5 (0.3, 0.7)	-2.2 (-2.8, -1.5)	-2.3 (-2.9, -1.7)	-2.5 (-3.2, -1.8)	0.4 (-0.5, 1.3)	-0.3 (-1.0, 0.4)	-0.7 (-1.7, 0.2)	0.6 (-0.4, 1.6)	-0.4 (-0.9, 0.2)	-0.6 (-1.4, 0.2)
Viet Nam	0.9 (0.8, 1.0)	0.7 (0.6, 0.8)	0.5 (0.5, 0.6)	0.2 (0.1, 0.2)	0.1 (0.0, 0.1)	-0.2 (-0.3, -0.1)	0.0 (-0.1, 0.1)	-0.5 (-0.6, -0.5)	-0.6 (-0.7, -0.6)	2.3 (2.2, 2.5)	1.1 (1.1, 1.2)	0.9 (0.8, 1.0)
Yemen	1.2 (1.0, 1.4)	1.0 (0.8, 1.1)	1.1 (1.0, 1.2)	-1.0 (-1.2, -0.9)	-1.0 (-1.1, -0.8)	-1.2 (-1.4, -1.1)	0.6 (0.4, 0.8)	0.7 (0.5, 0.9)	0.6 (0.3, 0.8)	1.8 (1.6, 2.0)	1.3 (1.2, 1.4)	1.2 (1.2, 1.3)
Zambia	0.9 (0.8, 1.0)	0.7 (0.5, 0.9)	0.6 (0.3, 0.8)	-1.0 (-1.2, -0.9)	-0.8 (-1.0, -0.7)	-1.5 (-1.7, -1.3)	-0.1 (-0.3, 0.1)	0.0 (-0.2, 0.2)	-0.1 (-0.5, 0.2)	1.9 (1.7, 2.0)	1.5 (1.3, 1.6)	1.5 (1.3, 1.7)
Zimbabwe	0.6 (0.1, 1.1)	0.4 (-0.1, 0.8)	0.6 (0.0, 1.2)	0.3 (0.0, 0.6)	0.2 (-0.1, 0.6)	0.5 (0.1, 1.0)	0.7 (0.3, 1.0)	0.6 (0.3, 1.0)	0.8 (0.4, 1.3)	0.5 (0.1, 0.9)	0.6 (0.2, 0.9)	0.6 (0.2, 1.0)

Abbreviations: AAPC, average annual percentage change; DALY, disability-adjusted life year; CI, confidence interval; AML, acute myeloid leukemia; CML,

chronic myeloid leukemia; ALL, acute lymphoblastic leukemia; CLL, chronic lymphocytic leukemia.
