# Supplementary material for: The Global Burden of Leukemia and Its Attributable Factors in 204 Countries and Territories: Findings from the Global Burden of Disease 2019 Study and Projections to 2030
Source: J Oncol. 2022 Apr 25;2022:1612702. doi: 10.1155/2022/1612702 (PMC9061017; doi:10.1155/2022/1612702)
Supplement: Supplementary Materials — Supplementary Table 1: the numbers, age-standardized rates, and AAPCs in incidence, death, and DALYs for leukemia in 204 countries and territories. Supplementary Table 2: the AAPCs of ASIR, ASDR, and age-standardized DALY rate due to 4 leukemia subtypes from 1990 to 2019 in 204 countries/territories. Supplementary Figure 1: proportional distribution of incident cases, deaths, and DALYs by the age group. Supplementary Figure 2: radar maps of incident cases (a), deaths (b), and DALYs (c) among SDI quintiles due to 4 subtypes of leukemia across different age groups. Supplementary Figure 3: the correlation between AAPC of ASIR (a), ASDR (b), age-standardized DALY rate (c), and SDI for 4 leukemia subtypes in 2019. Supplementary Figure 4: chordal graphs of risk factors contributing to leukemia-related deaths (a) and DALYs (b) by sex. Supplementary Figure 5: chordal graphs of risk factors contributing to leukemia-related deaths (a) and DALYs (b) among different age groups. [file 1612702.f1.zip › k.Supplementary Figure 2.pdf]

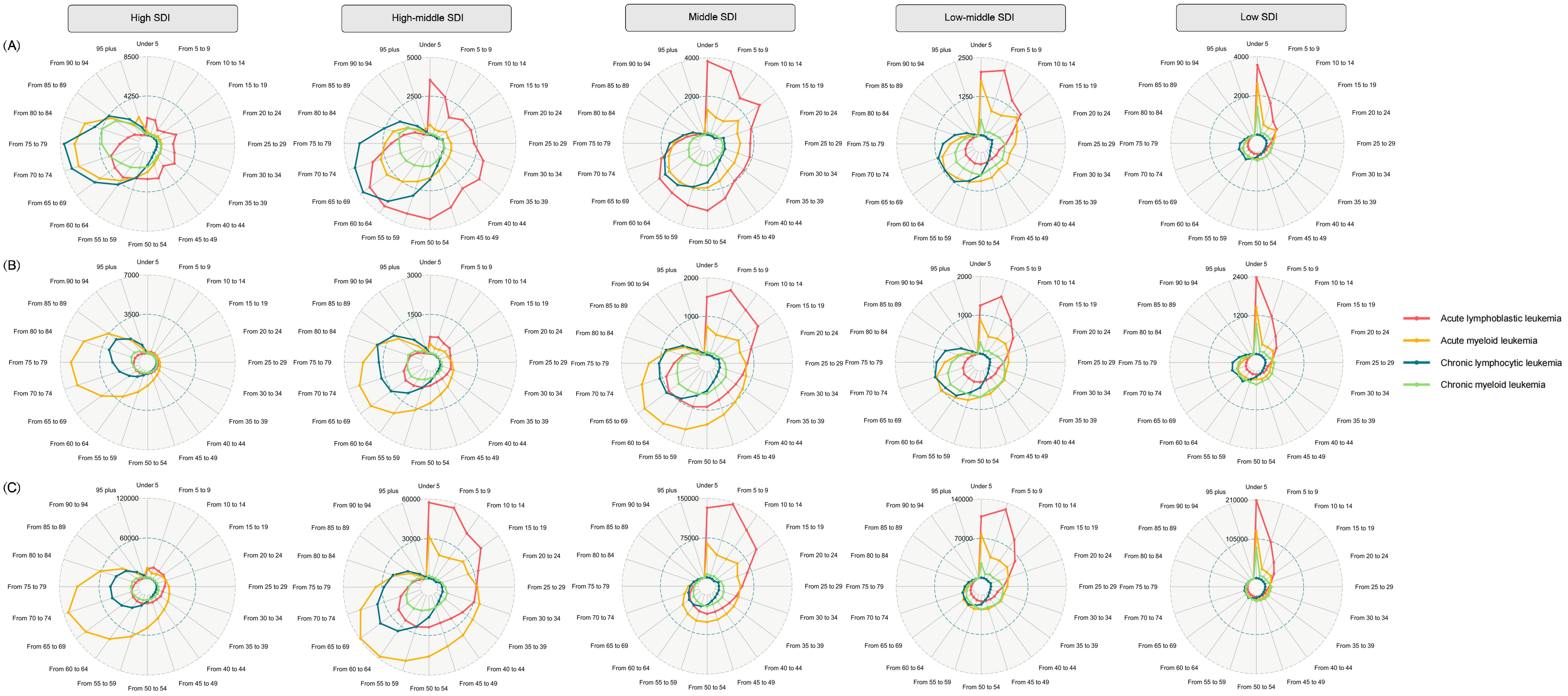

High SDI
High-middle SDI
Middle SDI
Low-middle SDI
Low SDI
(A)
(B)
(C)
Under 5
From 5 to 9
From 10 to 14
From 15 to 19
From 20 to 24
From 25 to 29
From 30 to 34
From 35 to 39
From 40 to 44
From 45 to 49
From 50 to 54
From 55 to 59
From 60 to 64
From 65 to 69
From 70 to 74
From 75 to 79
From 80 to 84
From 85 to 89
From 90 to 94
95 plus
8500
4250
5000
2500
4000
2000
2500
1250
4000
2000
7000
3500
3000
1500
2000
1000
2000
1000
2400
1200
120000
60000
60000
30000
150000
75000
140000
70000
210000
105000
Acute lymphoblastic leukemia
Acute myeloid leukemia
Chronic lymphocytic leukemia
Chronic myeloid leukemia
