# Supplementary material for: The Global Burden of Leukemia and Its Attributable Factors in 204 Countries and Territories: Findings from the Global Burden of Disease 2019 Study and Projections to 2030
Source: J Oncol. 2022 Apr 25;2022:1612702. doi: 10.1155/2022/1612702 (PMC9061017; doi:10.1155/2022/1612702)
Supplement: Supplementary Materials — Supplementary Table 1: the numbers, age-standardized rates, and AAPCs in incidence, death, and DALYs for leukemia in 204 countries and territories. Supplementary Table 2: the AAPCs of ASIR, ASDR, and age-standardized DALY rate due to 4 leukemia subtypes from 1990 to 2019 in 204 countries/territories. Supplementary Figure 1: proportional distribution of incident cases, deaths, and DALYs by the age group. Supplementary Figure 2: radar maps of incident cases (a), deaths (b), and DALYs (c) among SDI quintiles due to 4 subtypes of leukemia across different age groups. Supplementary Figure 3: the correlation between AAPC of ASIR (a), ASDR (b), age-standardized DALY rate (c), and SDI for 4 leukemia subtypes in 2019. Supplementary Figure 4: chordal graphs of risk factors contributing to leukemia-related deaths (a) and DALYs (b) by sex. Supplementary Figure 5: chordal graphs of risk factors contributing to leukemia-related deaths (a) and DALYs (b) among different age groups. [file 1612702.f1.zip › l.Supplementary Figure 3.pdf]

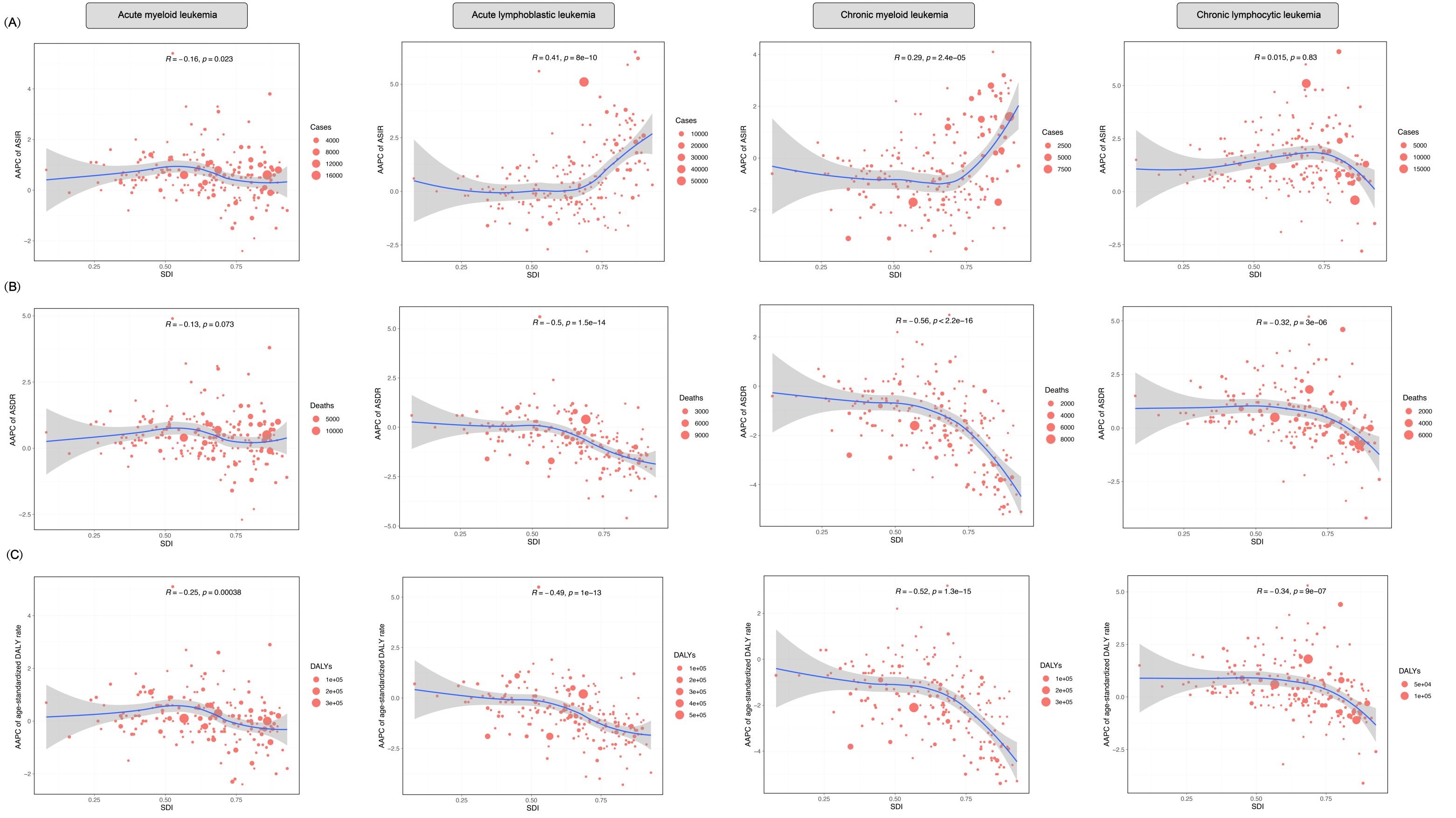

(A)
(B)
(C)
Acute myeloid leukemia
Acute lymphoblastic leukemia
Chronic myeloid leukemia
Chronic lymphocytic leukemia
R = −0.16, p = 0.023
R = 0.41, p = 8e−10
R = 0.29, p = 2.4e−05
R = 0.015, p = 0.83
R = −0.13, p = 0.073
R = −0.5, p = 1.5e−14
R = −0.56, p < 2.2e−16
R = −0.32, p = 3e−06
R = −0.25, p = 0.00038
R = −0.49, p = 1e−13
R = −0.52, p = 1.3e−15
R = −0.34, p = 9e−07
AAPC of ASIR
AAPC of ASDR
AAPC of age-standardized DALY rate
SDI
Cases
Deaths
DALYs
