# Supplementary figures and images for: The Global Burden of Leukemia and Its Attributable Factors in 204 Countries and Territories: Findings from the Global Burden of Disease 2019 Study and Projections to 2030
Source: J Oncol. 2022 Apr 25;2022:1612702. doi: 10.1155/2022/1612702 (PMC9061017; doi:10.1155/2022/1612702)

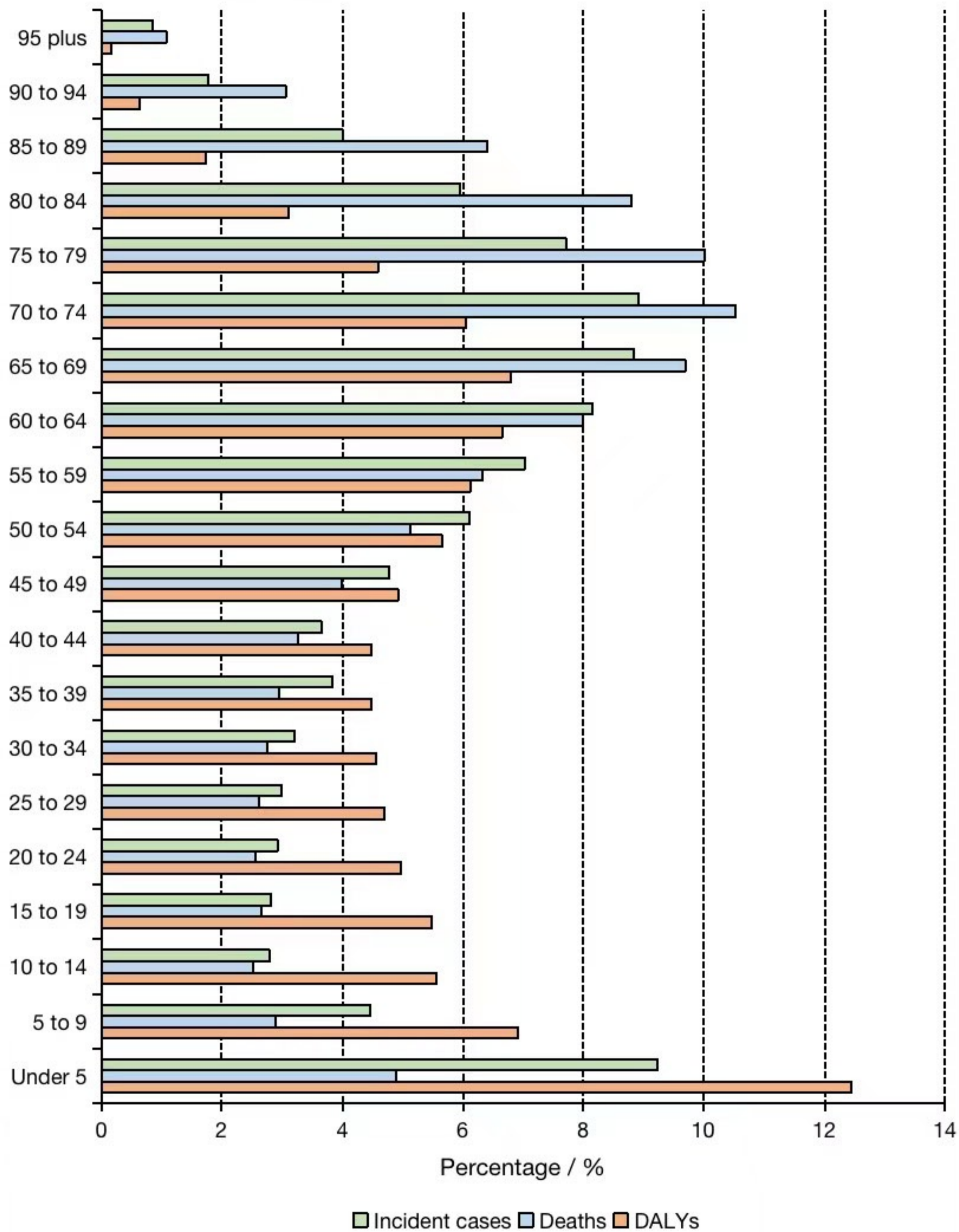

Supplement: Supplementary Materials — Supplementary Table 1: the numbers, age-standardized rates, and AAPCs in incidence, death, and DALYs for leukemia in 204 countries and territories. Supplementary Table 2: the AAPCs of ASIR, ASDR, and age-standardized DALY rate due to 4 leukemia subtypes from 1990 to 2019 in 204 countries/territories. Supplementary Figure 1: proportional distribution of incident cases, deaths, and DALYs by the age group. Supplementary Figure 2: radar maps of incident cases (a), deaths (b), and DALYs (c) among SDI quintiles due to 4 subtypes of leukemia across different age groups. Supplementary Figure 3: the correlation between AAPC of ASIR (a), ASDR (b), age-standardized DALY rate (c), and SDI for 4 leukemia subtypes in 2019. Supplementary Figure 4: chordal graphs of risk factors contributing to leukemia-related deaths (a) and DALYs (b) by sex. Supplementary Figure 5: chordal graphs of risk factors contributing to leukemia-related deaths (a) and DALYs (b) among different age groups. [file 1612702.f1.zip › j.Supplementary Figure 1.pdf]

(A)

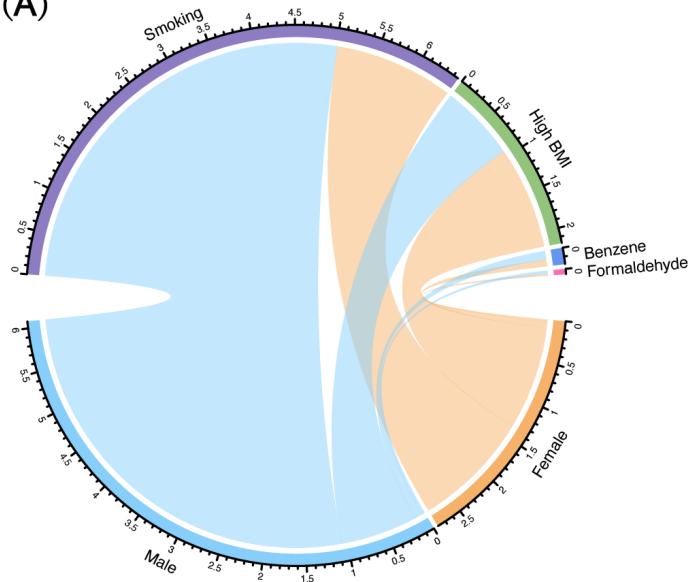

(B)

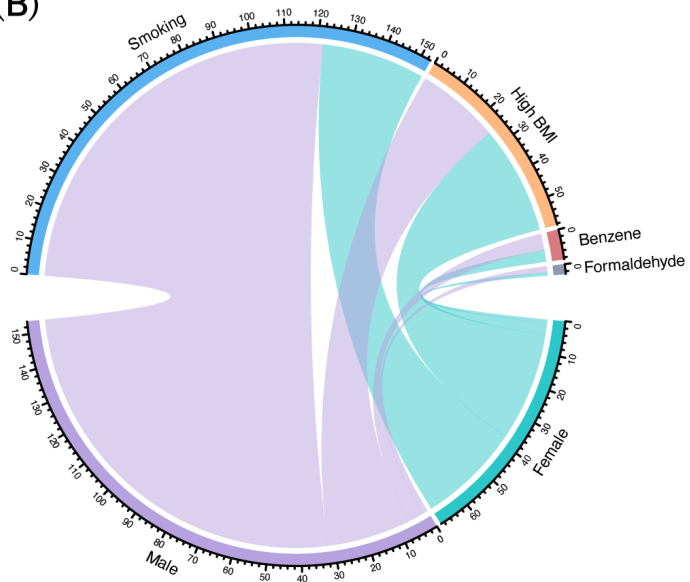

Supplement: Supplementary Materials — Supplementary Table 1: the numbers, age-standardized rates, and AAPCs in incidence, death, and DALYs for leukemia in 204 countries and territories. Supplementary Table 2: the AAPCs of ASIR, ASDR, and age-standardized DALY rate due to 4 leukemia subtypes from 1990 to 2019 in 204 countries/territories. Supplementary Figure 1: proportional distribution of incident cases, deaths, and DALYs by the age group. Supplementary Figure 2: radar maps of incident cases (a), deaths (b), and DALYs (c) among SDI quintiles due to 4 subtypes of leukemia across different age groups. Supplementary Figure 3: the correlation between AAPC of ASIR (a), ASDR (b), age-standardized DALY rate (c), and SDI for 4 leukemia subtypes in 2019. Supplementary Figure 4: chordal graphs of risk factors contributing to leukemia-related deaths (a) and DALYs (b) by sex. Supplementary Figure 5: chordal graphs of risk factors contributing to leukemia-related deaths (a) and DALYs (b) among different age groups. [file 1612702.f1.zip › m.Supplementary Figure 4.pdf]

(A)

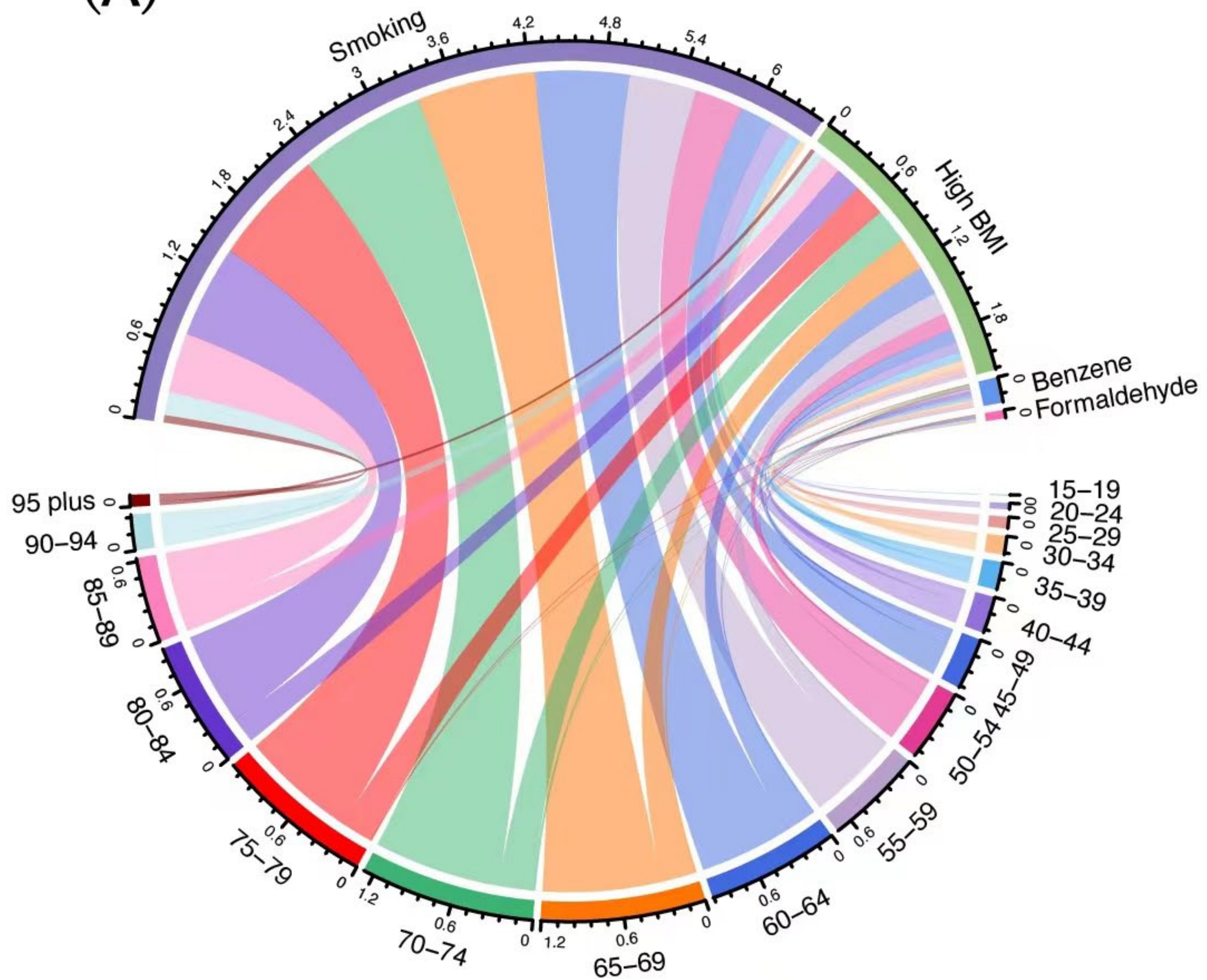

(B)

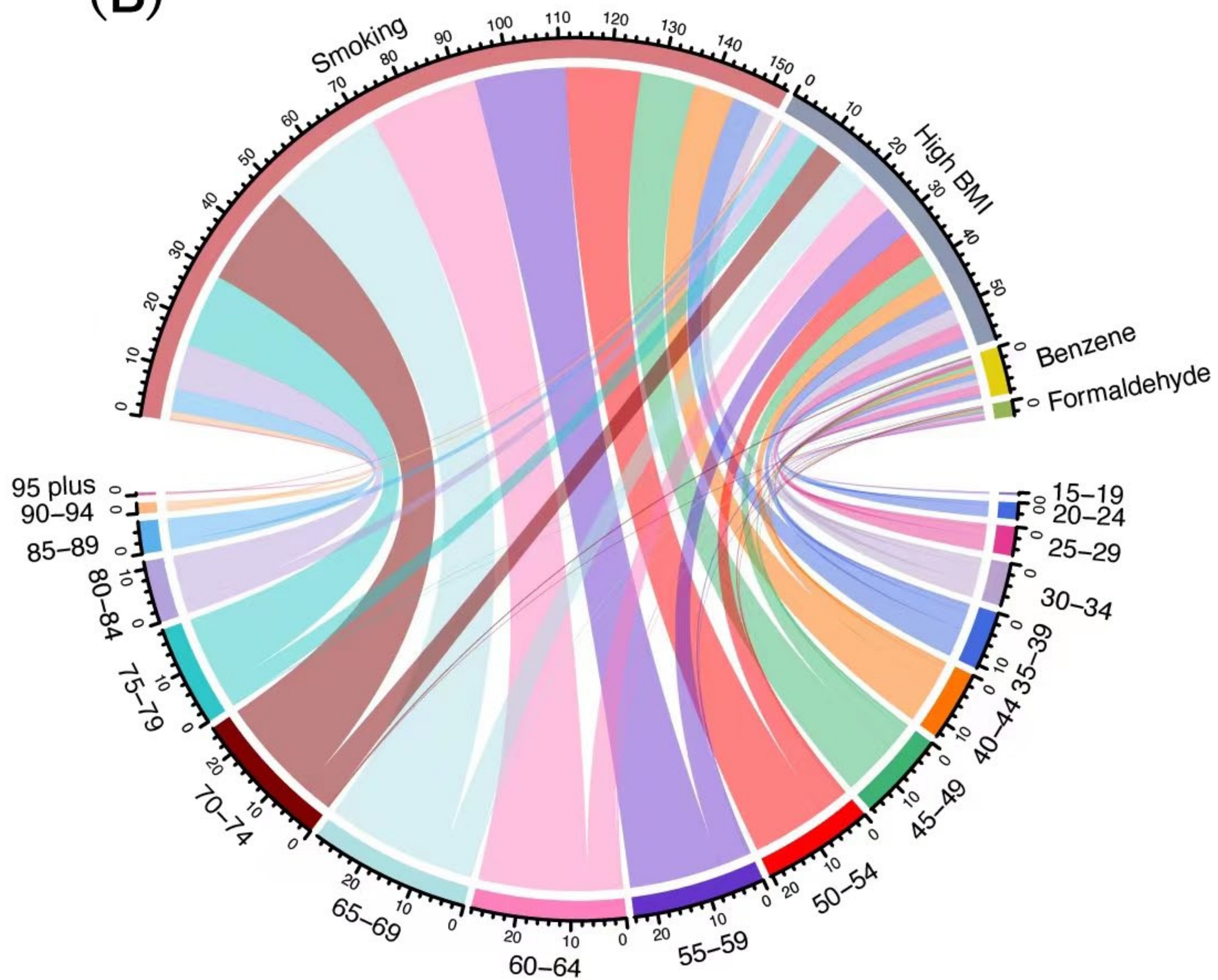

Supplement: Supplementary Materials — Supplementary Table 1: the numbers, age-standardized rates, and AAPCs in incidence, death, and DALYs for leukemia in 204 countries and territories. Supplementary Table 2: the AAPCs of ASIR, ASDR, and age-standardized DALY rate due to 4 leukemia subtypes from 1990 to 2019 in 204 countries/territories. Supplementary Figure 1: proportional distribution of incident cases, deaths, and DALYs by the age group. Supplementary Figure 2: radar maps of incident cases (a), deaths (b), and DALYs (c) among SDI quintiles due to 4 subtypes of leukemia across different age groups. Supplementary Figure 3: the correlation between AAPC of ASIR (a), ASDR (b), age-standardized DALY rate (c), and SDI for 4 leukemia subtypes in 2019. Supplementary Figure 4: chordal graphs of risk factors contributing to leukemia-related deaths (a) and DALYs (b) by sex. Supplementary Figure 5: chordal graphs of risk factors contributing to leukemia-related deaths (a) and DALYs (b) among different age groups. [file 1612702.f1.zip › n.Supplementary Figure 5.pdf]
